# Mitochondrial fission induces immunoescape in solid tumors through decreasing MHC-I surface expression

**DOI:** 10.1038/s41467-022-31417-x

**Published:** 2022-07-06

**Authors:** Xinyuan Lei, Hsinyu Lin, Jieqi Wang, Zhanpeng Ou, Yi Ruan, Ananthan Sadagopan, Weixiong Chen, Shule Xie, Baisheng Chen, Qunxing Li, Jue Wang, Huayue Lin, Xiaofeng Zhu, Xiaoqing Yuan, Tian Tian, Xiaobin Lv, Sha Fu, Xiaorui Zhu, Jian Zhou, Guokai Pan, Xin Xia, Bakhos A. Tannous, Soldano Ferrone, Song Fan, Jinsong Li

**Affiliations:** 1 Department of Oral and Maxillofacial Surgery, Sun Yat-Sen Memorial Hospital of Sun Yat-Sen University, Guangzhou, 510120 China; 2 Molecular and Cellular Biology, State University of New York at Stony Brook, Stony Brook, NY 11794 USA; 3 Guangdong Provincial Key Laboratory of Malignant Tumor Epigenetics and Gene Regulation of Sun Yat-Sen Memorial Hospital, Guangzhou, 510120 China; 4 Department of Surgery, Massachusetts General Hospital, Harvard Medical School, Boston, MA 02114 USA; 5 Massachusetts Institute of Technology, Cambridge, MA 02139 USA; 6 Department of Stomatology, Longgang District Central Hospital, Affiliated to Guangzhou University of Traditional Chinese Medicine, Shenzhen, 518116 China; 7 Department of Thoracic Surgery, Sun Yat-Sen Memorial Hospital of Sun Yat-Sen University, Guangzhou, 510120 China; 8 Cellular Molecular Diagnostics Center, Sun Yat-Sen Memorial Hospital of Sun Yat-Sen University, Guangzhou, 510120 China; 9 Breast Tumor Center, Sun Yat-Sen Memorial Hospital of Sun Yat-Sen University, Guangzhou, 510120 China; 10 Department of Neurobiology, Key Laboratory of Human Functional Genomics of Jiangsu, Nanjing Medical University, Nanjing, 211166 China; 11 Nanchang Key Laboratory of Cancer Pathogenesis and Translational Research, Center Laboratory, the Third Affiliated Hospital, Nanchang University, Nanchang, 330047 China; 12 Markey Cancer Center, the University of Kentucky, College of Medicine, Lexington, KY 40506 USA; 13 Department of Chronic Diseases Epidemiology, Yale University of Public Health, New Haven, CT 06520 USA; 14 Department of Medical Imaging, Sun Yat-Sen University Cancer Center, State Key Laboratory of Oncology in South China, Collaborative Innovation Center for Cancer Medicine, Guangzhou, 510060 China; 15 Experimental Therapeutics and Molecular Imaging Lab, Department of Neurology, Massachusetts General Hospital and Harvard Medical School, Boston, MA 02129 USA

**Keywords:** Cancer metabolism, Immunosurveillance, Energy metabolism

## Abstract

Mitochondrial dynamics can regulate Major Histocompatibility Complex (MHC)-I antigen expression by cancer cells and their immunogenicity in mice and in patients with malignancies. A crucial role in the mitochondrial fragmentation connection with immunogenicity is played by the IRE1α-XBP-1s axis. XBP-1s is a transcription factor for aminopeptidase TPP2, which inhibits MHC-I complex cell surface expression likely by degrading tumor antigen peptides. Mitochondrial fission inhibition with Mdivi-1 upregulates MHC-I expression on cancer cells and enhances the efficacy of adoptive T cell therapy in patient-derived tumor models. Therefore mitochondrial fission inhibition might provide an approach to enhance the efficacy of T cell-based immunotherapy.

## Abstract (web summary)

Cancer cells downregulate surface expression of major histocompatibility complex I (MHC-I) for immune evasion. Here, the authors show that rapid mitochondrial fission activates the ER-stress response leading to reduced MHC-I complex formation and cell surface expression in solid cancer cells; moreover inhibition of mitochondrial fission increases the immune-mediated anticancer response in murine models.

## Introduction

Major histocompatibility complex (MHC)-I antigen processing machinery (APM) component downregulation is frequently associated with malignant transformation of cells. This abnormality results in a defective synthesis and/or expression of MHC-I heavy chain-β_2_-microglobulin (β_2_m)-tumor antigen-derived peptide complexes (MHC-I in all species, HLA-I in humans), negatively impacting the recognition of cancer cells by cognate cytotoxic T lymphocytes (CTLs), and potentially providing them with an immunoescape mechanism^1^. MHC-I defects are reported to occur in >50% of malignancies across all cancer types. Fortunately, the majority of defects arise from dysregulated signaling and/or epigenetic mechanisms, and therefore may be corrected by clinically relevant targeted strategies^2^. Immunotherapy has emerged as an effective treatment for patients with certain advanced cancers. Immune checkpoint inhibitors (ICIs) are at the forefront of the immunotherapy revolution, inducing impressive clinical responses in advanced melanoma and non-small cell lung cancer (NSCLC) patients^3^. However, the failure of most patients to initially respond to therapy and the development of resistance in responders have proved significant challenges related to their application in clinical settings. MHC-I loss has been described as a mechanism of resistance to ICI therapy in lung cancer and melanoma patients^4^. These findings have stimulated interest in identifying the molecular mechanisms underlying MHC-I downregulation in malignant cells with the expectation that this information may contribute to the rational design of strategies to restore MHC-I expression by malignant cells.

The mitochondrion is one of the vital organelles in eukaryotic cells. As a dynamic organelle, it constantly undergoes fission and fusion, and has the unique ability to regulate its morphology in response to various cellular stimuli^5^. Mitochondrial hyper fragmentation has been observed in many cancer types. Notably, inhibition of mitochondrial fission leads to a decrease in cell proliferation and migration in models of thyroid, breast, lung, gastric, and colon cancer^6^. This finding highlights the potential role of a fragmented mitochondrial phenotype in the pathogenesis of many cancer types, though mitochondrial fragmentation has been linked to cancer cell immune escape to a limited extent.

Mitochondria are closely associated with the endoplasmic reticulum (ER), a central organelle with the primary function to process newly synthesized secretory and transmembrane proteins. Rapidly proliferating cancer cells require increased ER activity to facilitate the folding, assembly, and transport of membrane and secretory proteins, and are thereby subject to ER stress^7^. Adaptation to protein folding stress is mediated by the activation of an integrated signal transduction pathway named the unfolded protein response (UPR)^8^, which results in the activation of three ER transmembrane kinases, namely, protein kinase-like endoplasmic reticulum kinase (PERK), inositol requiring 1α (IRE1α), and activating transcription factor 6 (ATF6), which are otherwise maintained in an inactive state by the association of their luminal domains with the chaperone BiP/GRP78^9^.

Mitochondria are a primary source of reactive oxygen species (ROS) in cells. ROS are small molecules that are highly reactive due to the presence of unpaired electrons. Excessive mitochondrial fission may lead to changes in the structural organization and arrangement of electron transport chain (ETC) components within the mitochondrial membrane. These changes may perturb ETC activity, causing ROS overproduction^10^. The latter may mediate inflammation, and recent findings have linked ER stress to the generation and accumulation of intracellular ROS, a state commonly referred to as oxidative stress. Indeed, accumulation of misfolded proteins in the ER causes calcium leakage, which contributes to the depolarization of the inner mitochondrial membrane, disrupting the ETC, and leading to additional ROS generation^11^. ER stress has also been reported to increase mitochondrial fission^12^. Through this positive feedback loop, ROS exacerbates ER stress and enhances mitochondrial ROS generation^13^. Inhibition of mitochondrial fission effectively reduces mitochondrial ROS production and thus terminates the aforementioned cycle, also attenuating ER stress^14^.

Our present work shows that mitochondrial fission plays a key role in regulating MHC-I expression by cancer cells, as well as their immunogenicity by activating the IRE1α axis of the UPR pathway in vitro and in vivo. The current study proposes a model whereby mitochondrial fission contributes to cancer cell immunoescape, providing attractive targets for clinical therapeutics.

## Results

### Association of low pSer616 DRP-1 expression with high MHC-I expression on cancer cells and with improved prognosis in cancer patients

**Fig. 1:**
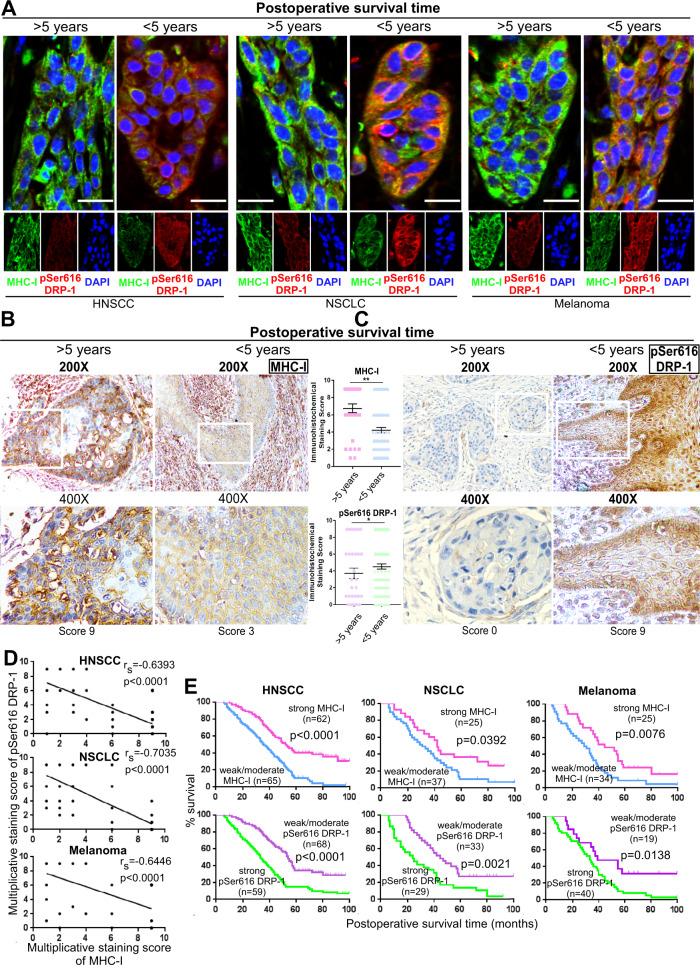
Low pSer616 DRP-1 correlates with high MHC-I and indicates a better prognosis in cancer patients. **A** Representative immunofluorescence images for MHC-I and pSer616 DRP-1 of HNSCC, NSCLC, and melanoma patients with different postoperative survival time (MHC-I: green, pSer616 DRP-1: red). DAPI, nuclear staining. Scale bars, 10 μm. **B**, **C** Representative immunochemistry images and scores for MHC-I and pSer616 DRP-1 of HNSCC patients with different postoperative survival time. Magnification, ×200 and ×400 (mean ± s.e.m; *p* < 0.0001 for MHC-I and *p* = 0.0173 for pSer616 DRP-1; **p < 0.001 by two-tailed *t*-test). **D** Spearman order correlation analysis of MHC-I and pSer616 DRP-1 association in HNSCC, NSCLC, and melanoma based on IHC score (*p* < 0.0001 for HNSCC, NSCLC and Melanoma) **E** Kaplan–Meier survival curves for HNSCC, NSCLC, and melanoma patients are plotted for pSer616 DRP-1 and MHC-I expression. Survival differences were analyzed using log-rank test (*p* < 0.0001 for HNSCC, *p* = 0.0392, *p* = 0.0021 for NSCLC and *p* = 0.0076, *p* = 0.0138 for Melanoma).

The mitochondrial fission process in mammals is mediated by dynamin-related protein 1 (DRP-1, *DNM1L*), mitochondrial fission 1 protein (FIS1), and mitochondrial fission factor; among them, the major role is played by the highly conserved DRP-1. It belongs to a family of large GTPases that self-assemble to regulate mitochondrial membrane structure^13^. DRP-1 is activated by phosphorylation at serine 616, a process mediated by regulatory kinases. Upon activation, DRP-1 migrates from the cytosol to the mitochondrion where it assembles in multimers that constrict and divide the organelle^15^. Conversely, phosphorylation of DRP-1 at serine 637 inhibits fission^16^. Tumor samples were obtained from 127 patients with head and neck squamous cell carcinoma (HNSCC), 62 patients with NSCLC and 59 patients with malignant melanoma at Sun Yat-Sen Memorial Hospital, Sun Yat-Sen University (Guangzhou, China) between January 2006 and December 2010 were used for MHC-I and phospo-Ser616 DRP-1 staining, Spearman correlation and Kaplan–Meier survival analysis. Both immunofluorescence (IF) and immunohistochemistry (IHC) staining revealed that the expression of MHC-I was much higher in tumor tissues from patients with longer survival (>5 years, post-surgery). In contrast, pSer616 DRP-1 level was significantly higher in patients who did not survive 5 years following surgery (Fig. 1A–C). Association of clinicopathological variables with MHC-I and pSer616 DRP-1 expression levels, as well as univariate and multivariate analysis of variables associated with overall survival (OS) of these cancer patients, is shown in Supplementary Tables 1 and 2. MHC-I and pSer616 DRP-1 expression levels were significantly associated with node metastasis in all the cancer types analyzed, but not consistently with gender or age (Supplementary Table 1). The expression levels were also prognostic markers in HNSCC, NSCLC, and malignant melanoma patients (Supplementary Table 2). Moreover, MHC-I expression level displayed a significant negative correlation with pSer616 DRP-1 expression level (Fig. 1D); high MHC-I expression level and low pSer616 DRP-1 expression level are both markers of significantly longer OS in the cancer patients tested (Fig. 1E). In addition to corroborating the previously reported the clinical significance of MHC-I and pSer616 DRP-1 expression levels^17,18^, our results, namely, the negative correlation between pSer616 DRP-1 and MHC-I expression levels, prompted us to hypothesize that targeting mitochondrial fission may represent a strategy to enhance MHC-I expression by cancer cells.

### Mdivi-1 enhances MHC-I expression in mouse tumor models

**Fig. 2:**
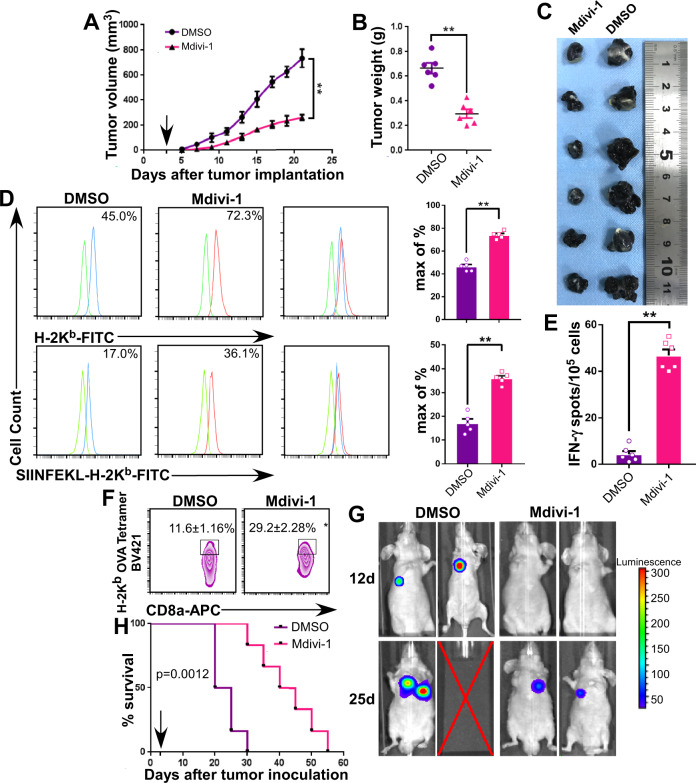
Mdivi-1 mediates MHC-I expression in syngeneic tumor models. **A** Tumor volume measurements after subcutaneous implantation, arrow indicates the time of Mdivi-1 or DMSO treatment (mean ± s.e.m; *n* = 6; *p* = 0.0124; **p < 0.001 by two-way ANOVA followed by Dunnett’s tests for multiple comparisons). **B** Weights of harvested tumors (*n* = 6; *p* < 0.001; **p < 0.001 by two-tailed *t*-test). **C** Photograph of harvested tumors. **D** Membrane expression of H-2K^b^ and SIINFEKL-H-2K^b^ complex of isolated primary cancer cells from harvested tumors by flow cytometry (mean ± s.e.m; *n* = 5; *p* < 0.0001 for H-2K^b^ and SIINFEKL-H-2K^b^ complex; **p < 0.001 by two-tailed *t*-test). **E** IFN-γ-producing CTLs as quantified by ELISpot (mean ± s.e.m; *n* = 6; *p* < 0.0001; **p < 0.001 by two-tailed *t*-test). **F** The isolated tumor-infiltrated CD8^+^ T cells were stained with anti-OVA-H-2K^b^ tetramer and anti-CD8a, and the percentages of infiltrated OVA-specific CTLs were quantified by flow cytometry (mean ± s.e.m; *n* = 3; *p* = 0.0012; *p < 0.01 by two-tailed *t*-test). **G** Biodistribution of cancer cells 12 and 25 days after inoculation (*n* = 6). Color scales represent photon intensities. **H** Kaplan–Meier survival curve was plotted for tail vein tumor inoculation model, and survival difference was analyzed using log-rank test (*n* = 6; *p* = 0.0012).

To investigate the role of DRP-1 in the regulation of MHC-I expression and its functional properties, we used mitochondrial division inhibitor 1 (Mdivi-1), a selective cell-permeable inhibitor of DRP-1. It inhibits the self-assembly of DRP-1 by blocking DRP-1’s GTPase activity^19^. By using immunocompetent C57BL/6 mice and B16F10 melanoma cells expressing the model tumor antigen, chicken-derived ovalbumin (OVA), we found that injection of Mdivi-1 via tail vein 3 days after tumor subcutaneous implantation significantly slowed down tumor growth compared to vehicle control (DMSO), as indicated by tumor volume measurement (Fig. 2A) and harvested tumor weight (Fig. 2B, C). Flow cytometric analysis of cancer cells isolated from the harvested tumors and stained with mAbs showed that H-2K^b^ antigen and immunodominant (ID) OVA epitope SIINFEKL^20^ expression was upregulated (Fig. 2D) on cancer cells isolated from tumors with reduced size. These phenotypic changes were associated with an increased number of IFN-γ-producing T cells (Fig. 2E) and intratumoral CTL infiltration (Fig. 2F). These results paralleled those obtained in mice injected with tumor cells via the tail vein. Monitoring the latter model with an IVIS Lumina imaging system showed that Mdivi-1 treatment efficiently prevented metastasis spread (Fig. 2G); furthermore, the survival of tumor-bearing mice was prolonged (Fig. 2H). Lastly, to confirm that the anti-tumor activity of Mdivi-1 depends on the immune system, B16F10 cells were injected into Rag1^-/-^ C57BL/6 mice, which lack T and B cells^21^, and the mice were treated with Mdivi-1 or DMSO. Treatment with Mdivi-1 did not inhibit tumor growth (Supplementary Fig. 1A–C), indicating that the anti-tumor effects of DRP-1 inhibition (Mdivi-1 treatment) are dependent on the presence of lymphocytes. In recent years, Mdivi-1 has emerged as a promising therapeutic agent for stroke, myocardial infarction, neurodegenerative diseases, and cancers^21^. Our results suggest that Mdivi-1’s anticancer activity may be mediated at least partially by MHC-I antigen upregulation on cancer cells, which contributes to their improved recognition and elimination by cognate CTLs. For confirming the effect of Mdivi-1 treatment of mice on the various immune cell populations within tumors. we then harvested tumors and performed IHC with monoclonal antibodies to identify the type of infiltrating immune cells (Supplementary Fig. 1D). We did not find a significant difference in the characteristics of the infiltrating immune cells in tumors isolated from Mdivi-1 and DMSO-treated mice.

### Mdivi-1 enhances the efficacy of adoptive T cell therapy (ACT) in PDX tumor models by upregulating MHC-I expression on cancer cells

**Fig. 3:**
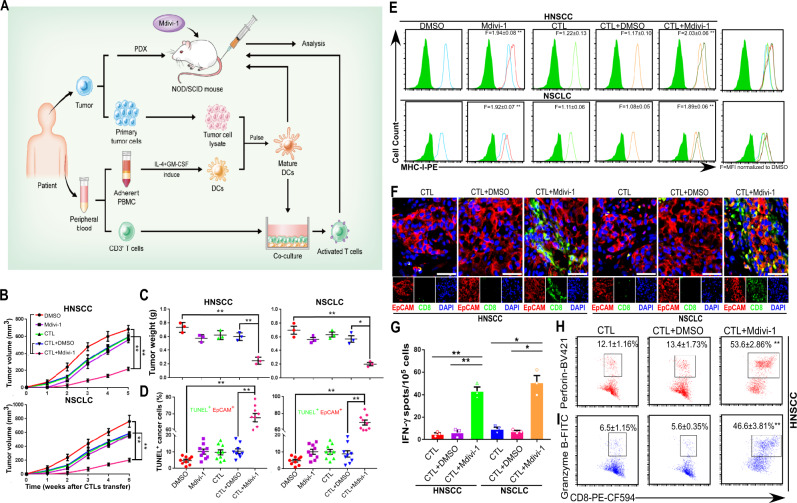
Mdivi-1 improves the adoptive T cell therapy (ACT) in PDX tumor models by upregulating MHC-I. **A** Scheme of ACT therapy through tumor-specific CTLs transferred into NOD/SCID mice transplanted with autologous HNSCC or NSCLC PDXs. **B** Tumor volume measurements were monitored weekly after ACT for five consecutive weeks (mean ± s.e.m; *n* = 3 per PDX group; *p* < 0.0001 for both HNSCC and NSCLC; **p < 0.001 by two-way ANOVA followed by Dunnett’s tests for multiple comparisons). **C** Weights of harvested HNSCC or NSCLC PDXs (mean ± s.e.m; *n* = 3 per PDX group; *p* = 0.0003, 0.0006 for HNSCC and 0.0006, 0.0011 for NSCLC; *p < 0.01, **p < 0.001 by one-way ANOVA followed by Dunnett’s tests for multiple comparisons). **D** Apoptosis of cancer cells as determined by EpCAM and TUNEL immunostaining (mean ± s.e.m; *n* = 9, 3 sections per PDX; *p* < 0.0001 for both HNSCC and NSCLC; **p < 0.001 by one-way ANOVA followed by Dunnett’s tests for multiple comparisons). **E** Flow cytometric analysis of MHC-I membrane expression in isolated HNSCC and NSCLC primary cancer cells. F indicates the fold change of MFI (mean fluorescence intensity) normalized to DMSO group (mean ± s.e.m; *n* = 3 per PDX group; *p* < 0.0001 or = 0.0792, 0.3609, 0.0002 for HNSCC and 0.0002, 0.0792, 0.3855, 0.0008 for NSCLC; **p < 0.001 compared with ACT accompanied by DMSO treatment or DMSO alone by one-way ANOVA followed by Dunnett’s tests for multiple comparisons). **F** Representative immunofluorescence images for EpCAM and CD8 of harvested PDXs (EpCAM: red; CD8: green). DAPI, nuclear staining. Scale bars, 25 μm. **G** Number of IFN-γ-producing CTLs as quantified by ELISpot (mean ± s.e.m; *n* = 3 per PDX group; *p* = 0.0002, 0.0004 for HNSCC and 0.0017, 0.0013 for NSCLC; *p < 0.01, **p < 0.001 by two-tailed *t*-test). **H**, **I** Evaluation of intracellular markers in association with cytotoxic function by flow cytometry (mean ± s.e.m; *n* = 3 per PDX group; *p* = 0.3405 or *p* < 0.0001 for perforin and 0.8773 or *p* < 0.0001 for granzyme B; **p < 0.001 compared with ACT accompanied by DMSO treatment by two-tailed *t*-test).

To further investigate the clinical relevance of our findings, we explored whether Mdivi-1 could arouse the immune dormancy and improve ACT effects in cancer patient-derived xenograft (PDX) model implanted in immunocompromised NOD/SCID mice (Fig. 3A). Successful PDX engraftments were established in 11 out of 63 (17.4%) primary HNSCC samples and 6 out of 32 (18.9%) primary NSCLC samples. Similarly, Mdivi-1 treatment effectively inhibited tumor growth, as evaluated through tumor volume measurements (Fig. 3B) and the harvested PDX tumor weights (Fig. 3C). Double immunostaining in the PDX tissues also showed consistent results as the above, indicating that adoptive transfer with CTLs and Mdivi-1 treatment resulted in massive apoptosis of cancer cells (EpCAM^+^ TUNEL^+^) (Fig. 3D and Supplementary Fig. 2A). We then dissociated the PDX and found that Mdivi-1 treatment significantly upregulated MHC-I membrane expression level irrespective of ACT (Fig. 3E) as in syngeneic models, providing a mechanism that may play a role in the observed synergy. A concomitant increase in both CD8^+^ T cell infiltration within cancer nests (Fig. 3F) and IFN-γ-producing immune cells^22^ (Fig. 3G) was also observed. Moreover, we isolated tumor-infiltrating CTLs and observed that perforin and granzyme B, markers associated with cytolytic activity^23^, were significantly upregulated after Mdivi-1 treatment (Fig. 3H, I and Supplementary Fig. 2B). Overall, these findings indicate that mitochondrial fission inhibition in PDX models can potentially reverse tumor immune evasion by upregulating MHC-I expression on cancer cells and improve the efficacy of ACT.

### Mdivi-1 improves the cytotoxic activity of T cells against autologous cancer cells in vitro

**Fig. 4:**
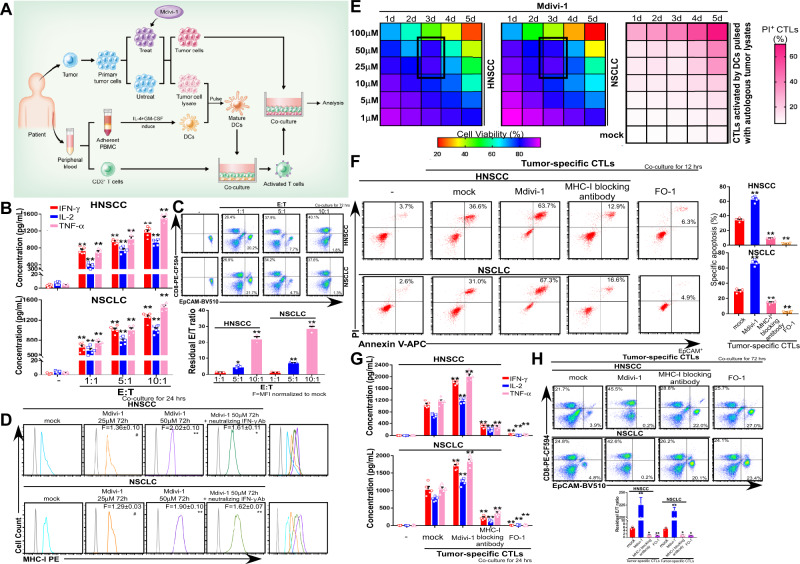
Mdivi-1 improves the cytotoxic function of CTLs targeting autologous cancer cells in vitro. **A** Scheme of tumor-specific CTLs generation and in vitro coculture. **B** Twenty hours later, Th1 cytokines production was quantified by specific ELISAs (mean ± s.e.m; *n* = 5; *p* < 0.0001 for HNSCC and NSCLC; **p < 0.001 compared with non-coculture group by two-tailed *t*-test). **C** Residual E/T ratio was evaluated by flow cytometry after 3 days based on CD8 and EpCAM expression (mean ± s.e.m; *n* = 3; *p* = 0.0015 or *p* < 0.0001 for HNSCC and *p* < 0.0001, *p* < 0.0001 for NSCLC; *p < 0.01, **p < 0.001 compared with E/T ration = 1:1 group by two-tailed *t*-test). **D** Evaluation of MHC-I membrane expression in HNSCC and NSCLC primary cancer cells by flow cytometry. F indicates the fold change of MFI normalized to mock (mean ± s.e.m; *n* = 12; *p* = 0.0484, 0.0001, 0.0005 for HNSCC and 0.0487, 0.0001, 0.0001 for NSCLC ^#^*p* < 0.05, *p < 0.01, **p < 0.001 compared with mock by two-tailed *t*-test). **E** Cell viability of primary cancer cells and PI uptake of tumor-specific CTLs after sequential treatment by MTS and flow cytometry. **F** Twelve hours after coculture, EpCAM^+^ cancer cells were harvested and cell death was examined with flow cytometry based on the uptake of PI and Annexin V. E/T ratio was 5:1 (mean ± s.e.m; *n* = 4; *p* < 0.0001 for HNSCC and *p* < 0.0001 for NSCLC; **p < 0.001 compared with mock by two-tailed *t*-test). **G** Th1 cytokines production was quantified by specific ELISAs after 24 h. E/T ratio was 5:1 (mean ± s.e.m; *n* = 5; *p* < 0.0001 for HNSCC and NSCLC; **p < 0.001 compared with mock by two-tailed *t*-test). **H** Evaluation of residual E/T ratio by flow cytometry based on CD8 and EpCAM expression respectively following 3 days. E/T ratio was 5:1 (mean ± s.e.m; *n* = 3; *p* < 0.0001, *p* = 0.0011, 0.0009 for HNSCC and *p* < 0.0001, *p* = 0.0015, 0.0012 for NSCLC; *p < 0.01, **p < 0.001 compared with mock by two-tailed *t*-test). E indicates effector cells, namely T cells. T indicates targeted cells, namely cancer cells.

We next assessed the effects of Mdivi-1 on the in vitro cytotoxic activity of T cells (Fig. 4A). T cells were primed by tumor cell-lysate-pulsed dendritic cells (DCs) for 5 days^24^ and co-cultured with autologous cancer cells at different E/T (effector/target) ratios. After 12 h, death of cancer cell (indicated as EpCAM^+^) was examined by PI and Annexin V uptake using flow cytometry (Supplementary Fig. 2C). The production of Th1 cytokines^25^ and residual E/T ratio were quantified using an enzyme-linked immunosorbent assay (ELISA) following a 24-h incubation (Fig. 4B) and flow cytometry following a 3-day incubation (Fig. 4C) based on CD8 and EpCAM expression. The results indicate that DCs (Supplementary Fig. 2D) and CTLs can be generated and the cytotoxic activity of tumor antigen-specific CTLs is enhanced with increasing E/T ratio. To confirm Mdivi-1-mediated MHC-I upregulation was independent of secreted IFN-γ, we treated primary HNSCC and NSCLC cells with Mdivi-1 (25–50 μM, 72 h) and incubated the cells with an IFN-γ neutralizing antibody (10 μg/mL, 24 h)^26^. As we expected, treatment with Mdivi-1 upregulated MHC-I membrane expression level (Fig. 4D), which was only partially inhibited by IFN-γ neutralizing antibodies (Fig. 4D). These findings indicate that Mdivi-1 can enhance MHC-I expression level on cancer cells independent of IFN-γ. Next, in order to determine the optimal concentration and duration for Mdivi-1 in vitro treatment, we performed sequential treatment^27^ using an MTS Assay Kit and flow cytometry on primary HNSCC/NSCLC cells and primed T cells to evaluate cell viability (Fig. 4E). By pretreating cancer cells with Mdivi-1 at the aforementioned dose/duration, the cytotoxic function of primed T cells co-cultured with primary HNSCC or NSCLC cells was dramatically enhanced. This was quantified by significant increases in cancer cell apoptosis (PI/Annexin V uptake) (Fig. 4F), Th1 cytokine production (Fig. 4G), and residual E/T ratio (Fig. 4H) relative to DMSO-treated cells. In addition, preincubation of cancer cells with anti-MHC-I mAb W6/32^28^ abrogated nearly all cytotoxicity against cancer cells (Fig. 4H). Similar results were replicated using the human melanoma cell line FO-1, which does not express MHC-I because of a structural B2M mutation^10^, further confirming the restriction and crucial role of MHC-I in T cell cytotoxic activity (Fig. 4H). Together, the above data suggest that mitochondrial fission inhibition greatly enhances the in vitro cytotoxic activity of T cells.

### Inhibition of mitochondrial fission restores MHC-I trimolecular complex expression on cancer cells, but does not affect MHC-I subunit expression

We next sought to delineate the mechanism(s) underlying MHC-I upregulation by mitochondrial fission inhibitors. Given that chemical inhibitor may have off-target effects, the results obtained by Mdivi-1 were further confirmed using a DRP-1 siRNA (small interfering RNA)^11^. First, we investigated the effect of DRP-1 siRNA on the tongue squamous cell carcinoma (TSCC) cell line viability; the results showed that there was no significant difference in cell viability and apoptosis as compared to cells treated with Mdivi-1 (Fig. 4E and Supplementary Fig. 3A). Mitochondrial fission inhibition by Mdivi-1 or DRP-1 siRNA upregulated MHC-I membrane expression on TSCC cell lines SCC-9 and CAL-27, on primary HNSCC cancer cells and on melanoma cell line B16F10 (Supplementary Fig. 3B, C). The results obtained with the TSCC cell lines were further verified by IF staining (Supplementary Fig. 3D). We then extended the analysis to additional solid cancer types, including NSCLC, osteosarcoma, melanoma, and mouse melanoma (Supplementary Fig. 3E). As significant MHC-I upregulation was also observed upon DRP-1 siRNA treatment of these cell lines, the role of mitochondrial dynamics in the regulation of MHC-I expression may be broadly applicable to many cancer types. In addition, to verify our hypothesis, we used antioxidants N-acetylcysteine (NAC)^12^ and mitoTEMPO^12^, as well as pro-oxidant BAY-87-2243^14,29^ to treat SCC-9 and CAL-27 cells. Neutralizing ROS by NAC or mitoTEMPO upregulated MHC-I membrane expression on SCC-9 and CAL-27 cells (Supplementary Fig. 3F), a finding consistent with the effects of Mdivi-1. Conversely, activation of ROS by BAY-87-2243 downregulated MHC-I membrane expression (Supplementary Fig. 3F). Surprisingly, despite MHC-I upregulation on the cell surface, no significant change in MHC-I subunit expression was detected both at the protein (Supplementary Fig. 4A, B) and the mRNA level in siDRP-1-treated TSCC cells (Supplementary Fig. 4C, D). Furthermore, DRP-1 overexpression significantly downregulated MHC-I membrane expression on TSCC cells (Supplementary Fig. 4E) without affecting their viability (Supplementary Fig. 4F), whereas no significant change in MHC-I subunit protein and mRNA expression levels was detected (Supplementary Fig. 4G, H). These findings imply that mitochondrial fission regulates MHC-I trimolecular complex cell membrane expression on cancer cells at the post-translational level.

### Mitochondrial fission inhibition alleviates oxidative stress and UPR in cancer cells

**Fig. 5:**
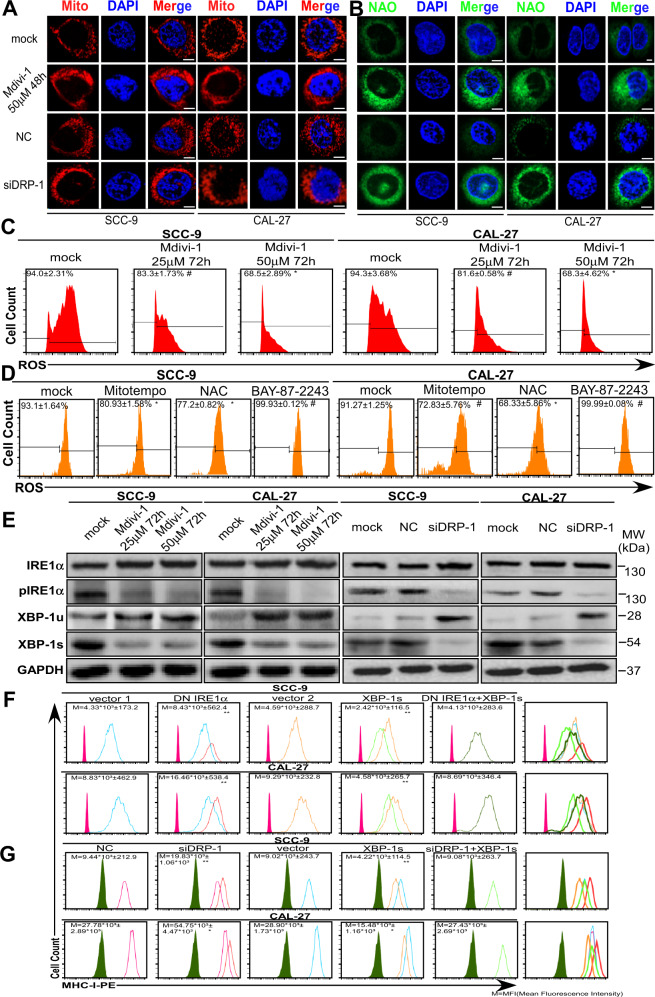
Inhibition of mitochondrial fission alleviates oxidative stress and UPR in cancer cells. **A** Mitochondrial morphology of TSCCs was visualized by fluorescent dye MitoTracker Red CMXRos (Mito: red). **B** Oxidation of cardiolipin in TSCC was assessed by fluorescent dye NAO (NAO: green). DAPI, nuclear staining. Scale bars, 2 μm. **C**, **D** Assessment of intracellular ROS by flow cytometry in TSCCs (mean ± s.e.m; *n* = 9; *p* = 0.0104, 0.0012 for SCC-9 Mdivi-1 treatment group and 0.0136, 0.0059 for CAL-27 Mdivi-1 treatment group; *p* < 0.0001, *p* < 0.0001 and *p* = 0.0004 for SCC-9; *p* = 0.0008, 0.0002 and 0.049 for CAL-27; ^#^*p* < 0.05, *p < 0.01, **p < 0.001). **E** Immunoblotting for associated ER stress markers in TSCCs. MW molecular weight. GAPDH, loading control. **F**, **G** Flow cytometric analysis of MHC-I membrane expression after depicted treatments in TSCCs (mean ± s.e.m; *n* = 4; the upper panel, *p* = 0.0001, 0.0001 and 0.4341 for SCC-9; *p* < 0.0001, <0.0001 and 0.0944 for CAL-27; the lower panel, *p* < 0.0001, *p* < 0.0001 and *p* = 0.8971 for SCC-9; *p* = 0.0004, <0.0001 and 0.6033 for CAL-27; *p < 0.01, **p < 0.001, DN IRE1α compared with vector 1 and IRE1α+XBP-1s, XBP-1s compared with vector 2 and IRE1α+XBP-1s; siDRP-1 compared with NC and siDRP-1+XBP-1s, XBP-1s compared with vector and siDRP-1+XBP-1s). M indicates mean fluorescence intensity. For experiments that were conducted at different time points, the isotype tubes were adjusted to ensure that their fluorescence intensity was at similar levels.

SCC-9 and CAL-27’s fragmented mitochondria were found to be distinctly transformed into a filamentous phenotype upon treatment with Mdivi-1 or siDRP-1 (Fig. 5A). This transformation was accompanied by changes in the staining of TSCC cells by dyes 10-N-nonyl acridine orange, which binds specifically to non-oxidized cardiolipin (Fig. 5B)^30^, and JC-1 (Supplementary Fig. 5A)^31^, an indicator of mitochondrial membrane potential. The significant decrease of intracellular ROS further validates the strategy of terminating the “stress cycle” through mitochondrial fission inhibition or antioxidant and pro-oxidant treatments (Fig. 5C, D and Supplementary Fig. 5B). ER stress and UPR were inhibited, as well, as evidenced by the inactivation of the IRE1α-XBP-1s pathway (Fig. 5E). In addition, DRP-1 overexpression significantly increased intracellular ROS levels, as expected (Supplementary Fig. 5C). These results all together indicate that mitochondrial fission inhibition is an effective method to attenuate oxidative stress within cancer cells.

### IRE1α-XBP-1s is the most significant regulator of MHC-I expression in UPR

Growing evidence suggests a connection between ER stress and MHC-I expression: the overexpression of an ER stress-inducing misfolded protein or the constitutive expression of nuclear ATF6 or XBP-1s downregulates MHC-I expression on 293T cells^32^. Similarly, ER stress induced by palmitate or glucose deprivation downregulates MHC-I expression by mouse thymoma cells. We used dominant negative (DN) plasmids and siRNA (GenePharma) to interfere with the three branches of the UPR: IRE1α, PERK, and ATF6. We found that IRE1α, the most conserved arm of the UPR^3^, is the most significant regulator of MHC-I expression by TSCC cells (Supplementary Fig. 6A). Within minutes of unfolded protein accumulation, BiP dissociates from PERK, IRE1α, and ATF6 and preferentially binds to the unfolded proteins, resulting in the activation of PERK and IRE1α via luminal domain homodimerization and autophosphorylation of ATF6 via proteolytic cleavage^9^. Active IRE1α then excises a 26-base intron in X-box binding protein 1 (XBP-1) mRNA. Re-ligation of spliced XBP-1 shifts the open reading frame, and its translation produces the homeostatic transcription factor spliced XBP-1 (XBP-1s). XBP-1s translocates to the nucleus where it binds to UPR elements (UPRE) and activates UPR target genes. On the other hand, unspliced XBP-1 (XBP-1u) gets degraded rapidly^33^. Knockdown of XBP-1s by siRNA (GenePharma) significantly upregulates MHC-I expression to a similar level as TSCC cells transfected with DN IRE1α (Supplementary Fig. 6B). While transfection of XBP-1s alone significantly downregulated MHC-I expression, co-transfection of XBP-1s and DN IRE1α had no impact on MHC-I expression, as expected (Fig. 5F). Despite inducing changes in the expression of MHC-I and ER stress markers GRP78, CHOP^34^, and XBP-1s (Fig. 5E and Supplementary Fig. 6C–F), HLA-A/B/C and B2M protein and mRNA expression levels were not significantly altered upon DN IRE1α transfection, XBP-1s knockdown, or XBP-1s transfection (Supplementary Fig. 6G–I). The results we obtained with TSCC cell lines following DRP-1 knockdown and DRP-1 overexpression were consistent (Fig. 5G).

### TPP2 is a direct target of XBP-1s

**Fig. 6:**
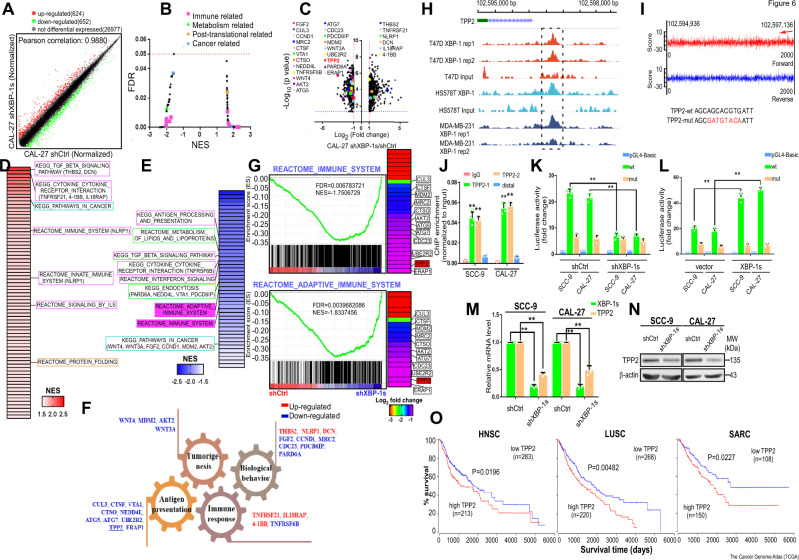
Bioinformatic analysis based on mRNA profile indicated TPP2 as the potential target of XBP-1s. **A** Scatter plot of relative mRNA expression in CAL-27 treated with shXBP-1s compared with negative control. Genes in green and red are differentially expressed at significant levels (*n* = 3 per group). **B** GSEA (KEGG, REACTOME, and BIOCARTA) pathway distribution for shXBP-1s versus negative control of CAL-27. Horizontal line denotes FDR significance cutoff of 0.05. Immune-, metabolism-, post-translation- and cancer-related gene sets were demarcated by dots in indicated colors, respectively. **C** Volcano plot of differentially expressed genes on the basis of fold change and *p* value. Genes to be focused on were labeled. **D** Gene sets upregulated in shXBP-1s CAL-27 compared to the negative control (FDR < 0.05 and NES > 1.5). Color gradation is based on GSEA NES. Gene sets demarcated in panel (**C**) were specified. **E** Gene sets downregulated in shXBP-1s compared to negative control of CAL-27 (FDR < 0.05 and NES < −1.5). Color gradation is based on GSEA NES. Gene sets demarcated in panel (**C**) were specified. Gene sets containing TPP2 were highlighted. NES indicates normalized enrichment score. **F** Diagrammatic drawing of associated genes divided into four groups based on respective functions that are recognized in cancer biology. **G** Downregulated genes from the two gene sets. Color gradation is representative of log2 fold change over negative control. Relevant genes were labeled and TPP2 was labeled in red. shCtrl indicates negative control. **H** Distribution of XBP-1 occupancy frequencies in TPP2 promoter in three different cancer cell lines based on ChIP-seq database, respectively. The most enriched peaks are highlighted. **I** Motif analysis (motif-counter) showed the enriched XBP-1 motif in TPP2 promoter, the arrow indicates that the highest score binding sites consistently located in the forward strand, which was highlighted in panel (**H**). **J** ChIP-qPCR analysis of the XBP-1 genomic occupancy in the TPP2 promoter in SCC-9 and CAL-27 as indicated. Immunoprecipitated DNA was measured by qRT-PCR with primers to amplify the TPP2 promoter region, including the distal site (mean ± s.e.m; *n* = 4; *p* < 0.0001, *p* < 0.0001, *p* = 0.3961 for SCC-9 and *p* < 0.0001, *p* < 0.0001, *p* = 0.408 for CAL-27; **p < 0.001 by one-way ANOVA followed by Dunnett’s tests for multiple comparisons). **K** Luciferase assay demonstrated that knockdown of XBP-1 inhibited TPP2 promoter’s activity in SCC-9 and CAL-27. SCC-9 and CAL-27 cells with stable expression of wild-type (wt) or mutant (mut) TPP2 promoter delivered pGL4.20-Basic vectors were co-transfected with shXBP-1 or shCtrl (mean ± s.e.m; *n* = 5; *p* < 0.0001 for SCC-9 and CAL-27; **p < 0.001 by one-way ANOVA followed by Dunnett’s tests for multiple comparisons). **L** Luciferase reporter assay demonstrated that XBP-1 activated TPP2 promoter (mean ± s.e.m; *n* = 3; *p* < 0.0001 for SCC-9 and CAL-27; **p < 0.001 by one-way ANOVA followed by Dunnett’s tests for multiple comparisons). **M** qRT-PCR verified the downregulation of TPP2 from microarrays in TSCCs after XBP-1s knockdown (mean ± s.e.m; *n* = 4; *p* < 0.0001 for SCC-9 and CAL-27; **p < 0.001 by two-tailed *t*-test). **N** Immunoblotting further verified the downregulation of TPP2 in TSCCs after XBP-1s knockdown. MW molecular weight. β-actin, loading control. **O** Overall survival of cancer patients with different levels of TPP2 using The Cancer Genome Atlas (TCGA) database (*p* = 0.0196, 0.00482 and 0.0227) HNSC head and neck squamous cell carcinoma, LUSC lung squamous cell carcinoma, SARC sarcoma.

Many potential mechanisms have been proposed regarding the regulation of MHC-I cell surface expression by ER stress signaling, including (i) removal or reduced synthesis of some unfolded proteins, possibly MHC-I subunits, to enable folding of essential or misfolded proteins^35^, (ii) ER stress blocking protein synthesis via PERK-mediated eIF2α phosphorylation, contributing to diminished peptide generation^36^, and/or (iii) downregulation of other APM components, such as the transporter associated with antigen presentation (TAP) involved in translocation of peptides from the cytosol to the ER lumen where they are loaded onto HLA class I heavy chain- β_2_m dimers^36,37^. To determine whether any of these proposed mechanisms are involved, we performed a microarray analysis following XBP-1s knockdown in CAL-27 TSCC cells to identify 1276 differentially expressed mRNAs (Fig. 6A–C). Gene set enrichment analysis (GSEA) revealed significant enrichment (*q* < 0.05) of several immune-, metabolism-, post-translational modification- and cancer-related gene sets (Fig. 6C–E). Notably, KEGG antigen processing/presentation genes and REACTOME adaptive immune system genes displayed negative enrichment (Fig. 6E–G). Among the most negatively enriched genes in the latter set, tripeptidyl peptidase 2 (TPP2), attracted our attention, as it is a well-recognized mammalian aminopeptidase that removes tripeptides from the N-terminus of longer peptides at neutral pH. TPP2 inhibition upregulates MHC-I expression on phytohemagglutinin-treated CD4+ and CD8+ T cells^38^. To determine whether XBP-1s directly binds to the *TPP2* gene, we reviewed the ENCODE chromatin immunoprecipitation-sequencing (ChIP-seq) database and found that the *TPP2* promoter displayed XBP-1 ChIP peaks in the breast cancer cell lines HS578T, MDA-MB-231, and T47D (Fig. 6H). We next performed de novo motif discovery on XBP-1 peaks and observed that the 5’-AGCAGCACGTGATT-3’ (reverse complement: 5’-TCGTCGTGCACTAA-3’) motif was highly enriched (Fig. 6I). ChIP and luciferase reporter assays identified the transcriptional functionality and binding of XBP-1s to the *TPP2* promoter (Fig. 6J–L). The results obtained from the microarray were corroborated by quantitative real-time PCR (qRT-PCR; Fig. 6M and Supplementary Fig. 7A) and immunoblotting (Fig. 6N), suggesting that TPP2 is a direct XBP-1s target. Moreover, a review of The Cancer Genome Atlas datasets showed that high TPP2 expression is associated with a significantly worse prognosis in many solid cancer types (Fig. 6O and Supplementary Fig. 7B).

### TPP2 inhibits MHC-I trimolecular complex expression by destroying antigenic peptides

**Fig. 7:**
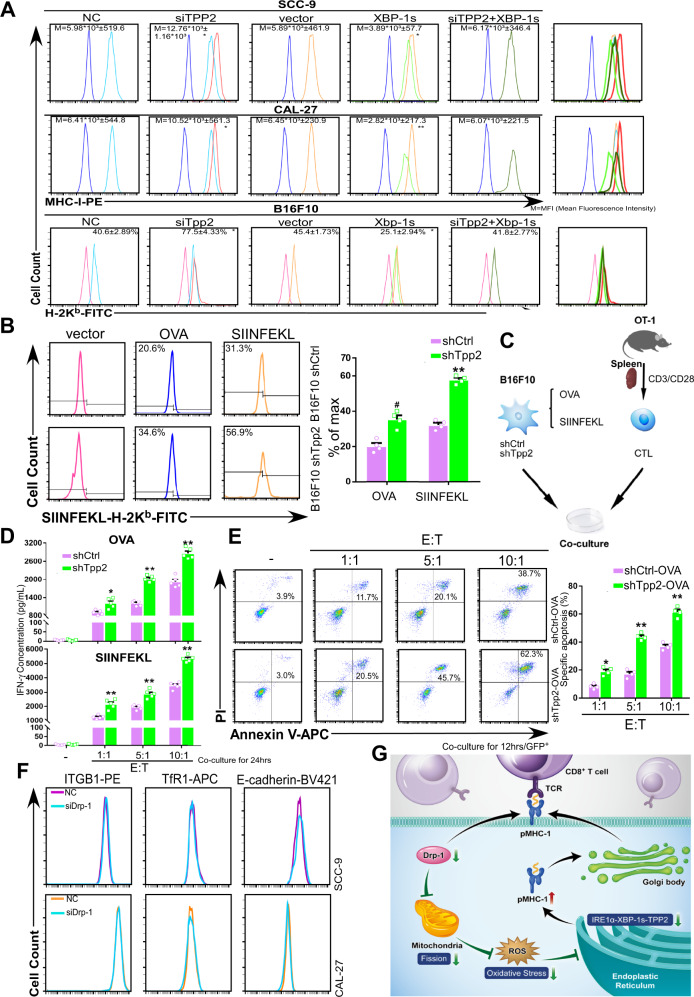
Destruction of antigenic peptides prevents MHC-I from maturation by TPP2. **A** Membrane expression of MHC-I in TSCCs and B16F10 was assessed by flow cytometry with indicated treatments (mean ± s.e.m; *n* = 6; *p* = 0.0029, 0.0063 for SCC-9, 0.0037, 0.0002 for CAL-27, 0.001, 0.002 for B16F10; *p < 0.01, **p < 0.001, siTPP2 compared with NC and siTPP2+XBP-1s, XBP-1s compared with vector and siTPP2+XBP-1s by two-way ANOVA followed by Dunnett’s tests for multiple comparisons). M indicates the mean fluorescence intensity. **B** SIINFEKL-H-2K^b^ complex expression was evaluated by flow cytometry of B16F10 (mean ± s.e.m; *n* = 4; *p* = 0.0113 and *p* < 0.0001; *p < 0.01, **p < 0.001 compared with shCtrl by two-tailed *t*-test). **C** Scheme of in vitro antigen presentation assays. **D** Quantification of mouse IFN-γ production by ELISA after 24 h (mean ± s.e.m; *n* = 5; *p* = 0.001, *p* < 0.0001, *p* < 0.001 for OVA and *p* < 0.0001 for SIINFEKEL; *p < 0.01, **p < 0.001 compared with shCtrl by two-tailed *t*-test). **E** Twelve hours after coculture, GFP^+^ cancer cells were harvested and cell death was examined by flow cytometry based on the uptake of PI and Annexin V (mean ± s.e.m; *n* = 4; *p* = 0.0003, *p* < 0.0001, *p* < 0.0001; *p < 0.01, **p < 0.001 compared with shCtrl by two-tailed *t*-test). E indicates effector cells namely T cells. T indicates targeted cells namely cancer cells. **F** Additional transmembrane molecules expression after DRP-1 knockdown was assessed by flow cytometry (*n* = 3). Both shCtrl and NC indicate negative control. **G** Graphic abstract of this study.

Antigen presentation is a multi-step process consisting of: (i) HLA class I heavy chain- β_2_m heterodimer formation, (ii) peptide generation, (iii) shuttling of peptides into the ER, (iv) peptide processing (both before and after shuttling), (v) loading of the peptide onto HLA class I heavy chain-β_2_m heterodimers, (vi) trafficking of the resulting trimolecular complex to the cell surface, and (vii) recognition of the trimolecular complex by cognate CD8^+^ T lymphocytes. During this process, small alterations can lead to the generation of suboptimally bound HLA class I heavy chain-β_2_m-peptide complexes that dissociate prematurely during their migration towards the cell surface^35^. siRNA-mediated TPP2 knockdown significantly upregulated MHC-I expression on TSCC and B16F10 melanoma cells, showing that TPP2 inhibits MHC-I expression (Fig. 7A). To further investigate its specific role in antigen processing and presentation, we used the B16F10 cell line that was stably transfected with either shTpp2 or shCtrl. When these cells were transiently transfected with OVA or SIINFEKL plasmids, Tpp2 knockdown significantly enhances SIINFEKL-H-2K^b^ complex expression (Fig. 7B). Stably transfected cells were then co-cultured with isolated OVA-specific T cells derived from OT-1 transgenic mice (Fig. 7C)^39^. Following Tpp2 knockdown, mouse IFN-γ concentration (Fig. 7D) and specific lysis of cancer cells (Fig. 7E) significantly increased at every E/T ratio tested. These results are consistent with the ability of Tpp2 to cleave antigenic peptides to sizes smaller than those required to bind to a histocompatibility antigen (<8–11 amino acids)^20^. Since TPP2 regulates cancer cell immunogenicity by degrading antigenic peptides, it can be inferred and experimentally verified that mitochondrial fission inhibition would not affect the expression of other immunological-related or unrelated transmembrane proteins (Fig. 7F). Therefore, degradation of antigenic peptides is a possible mechanism by which mitochondrial dynamics regulate cancer cell immunogenicity (Fig. 7G).

## Discussion

Immune escape of cancer cells is an important mechanism enabling unrestricted proliferation and tumor metastasis. Membrane MHC-I loss or downregulation is an important cause of escape from immunological surveillance^2^. It is found with high frequency in many solid cancer types, including malignant melanoma, breast cancer, stomach cancer, colon cancer, and bladder cancer^1^. In this study, we identified a low MHC-I membrane expression level on cancer cells as a biomarker of poor prognosis in HNSCC, NSCLC, and melanoma patients, corroborating prior findings^1,2,4^. However, only a few studies have attempted to restore MHC-I expression on cancer cells, reversing the immunoescape phenotype and promoting their CTL-mediated elimination.

Mitochondria are the indispensable regulatory centers shared by all eukaryotic cells and have always been a topic of basic research. In recent years, mitochondrial dynamics have received more attention, in addition to mitochondrial metabolism and apoptosis. Mitochondrial fission and fusion are both closely related to important physiological processes. For example, mitochondrial fission produces smaller, fragmented mitochondria, which are important for the mitochondrial movement to regions of high energy demand or to allow for equal mitochondrial distribution in daughter cells following mitosis^5^. Mitochondrial fission is also implicated in the release of cytochrome C into the cytosol to trigger apoptosis, and in the destruction of damaged cellular organelles^40^. More importantly, impaired fusion and enhanced fission have been frequently observed in many solid cancer types^41^. Mitochondrial fission is required to maintain the metastatic potential and high proliferation rate of glioblastoma, thyroid cancer, and breast cancer cells^18^. In addition, cancer cell growth may be blocked by mitochondrial fission inhibition through DRP-1 knockdown^6^. In the present study, we have shown that high pSer616 DRP-1 expression is a marker of poor prognosis in HNSCC, NSCLC, and melanoma patients. We have also shown that mitochondrial fission can be utilized as a promising target to regulate the immunogenicity of cancer cells, which can eventually influence the biological behavior and clinical outcome of several types of cancer. However, it is difficult to attribute the immunological effects of Mdivi-1 solely to MHC-I upregulation; impacts on immune cells cannot be excluded^42,43^.

Interestingly, fusion of mitochondria into linear or tubular networks limits deleterious mutations in mitochondrial DNA, induces the formation of ETC supercomplexes enhancing oxidative phosphorylation activity, promotes ER interactions important for Ca^2+^ flux, and protects mitochondria from autophagic degradation^44^. In addition, mitochondria elongate as a survival mechanism in response to nutrient starvation and stress, linking fusion to cell longevity and persistence^45^. These data indicate that inhibiting mitochondrial fission and promoting fusion in the adjuvant setting (in combination with T cell-based immunotherapy), may dramatically mitigate the deleterious side effects on normal cells that limit the clinical application of traditional anticancer therapies (chemotherapy, radiotherapy) at high doses.

ER stress caused by the abnormal accumulation of unfolded proteins in the ER is a hallmark of secretory cells and many diseases, including diabetes, neurodegeneration, and cancer^8^. The UPR can promote survival under conditions of transient and mild ER stress. For example, ER stress can promote angiogenesis by stimulating VEGF expression and secretion. It can also induce cancer cell dormancy through G1 arrest due to decreased cyclin D1 protein synthesis downstream of PERK activation. The UPR-induced upregulation of GRP78 and other ER chaperones can enhance the ER protein folding capacity and reestablish ER homeostasis, which protects cancer cells from apoptosis and allows for recurrence once favorable growth conditions return^46^. However, if ER stress is prolonged and severe, coupled with failure of compensatory mechanisms, apoptosis will be initiated through activation of the transcription factor CHOP downstream of PERK-eIF2α-ATF4 signaling. Although the molecular mechanisms underlying this switch remain poorly understood, each apical UPR sensor holds a dualistic role in propagating adaptive as well as pro-apoptotic signals^33^. Thus, from a simplistic viewpoint one would like to promote severe ER stress in cancer cells by therapeutics that either block the pro-survival pathways and/or promote the pro-apoptotic signals triggered by UPR, such as proteasome inhibitors^47^ to further increase the protein burden in the already challenged ER, or HSP90 inhibitors that can activate all three UPR branches^48^. The above methods are all aiming to further aggravate ER stress, and although they are cytotoxic to cancer cells, they will also damage normal cells. In recent years, several small-molecule inhibitors targeting the UPR, including the IRE1α-specific inhibitor 4μ8c^3^, ER stress inhibitor TUDCA^36^, and PERK kinase domain inhibitor GSK2606414 have been described^49^. However, their successful application is limited due to off-target effects and pancreatic toxicity^50^. Moreover, the molecular insights on apoptosis/survival decisions during ER stress are still too limited and the risk exists that the anticancer drug in question may block ER stress-mediated apoptosis, thereby promoting tumor progression. It is therefore not surprising that the data in the literature about the impact of PERK or IRE1α inhibition on cancer therapy are conflicting^47^. These drawbacks have provided us with the rationale to design a therapeutic strategy to target mitochondrial fission rather than ER stress.

Restoring MHC-I expression on cancer cells is an attractive strategy to trigger a CTL-mediated immune response against malignant cells. The previously described^32^ linkage between UPR and the MHC-I antigen presentation pathway has been confirmed in the present study. However, the broad impact of UPR on distinct cellular pathways, combined with the complexity of the antigen presentation pathway, makes the establishment of a mechanistic link between ER stress signaling and MHC-I downregulation a difficult task^35^. By using an expression microarray, bioinformatic analysis, ChIP-qPCR, and luciferase reporter assays, TPP2 has emerged as a plausible link between MHC-I expression and the XBP-1s branch of the UPR. Peptides that bind to MHC-I are produced from intracellular proteins as a byproduct of protein catabolism. The major proteins responsible for the initial cleavage of ubiquitinated cellular proteins into oligopeptides are the catalytic proteasome subunits. The majority of peptides produced by proteasomes are rapidly hydrolyzed into amino acids by the concerted action of aminopeptidases and endopeptidases in the cytosol. However, a small fraction of peptides escape destruction and are transported by TAP into ER where those with the right size and amino acid sequence bind to newly assembled HLA class I heavy chain -β_2_m heterodimeric complexes. Proteasomes frequently generate peptides that are too long to bind to MHC-I alleles but can serve as potential antigenic precursors. These long precursors can be converted to peptides that bind to HLA class I alleles by aminopeptidases or may be completely degraded to amino acids by aminopeptidases and endopeptidases^51^. Most aminopeptidases preferentially degrade relatively short peptides. They have little or no activity on peptides that are longer than about 16 amino acids when tested in vitro; in contrast, TPP2 has the ability to degrade longer peptides^52^. The aminopeptidase TPP2 is reported to be involved in the generation of many epitopes recognized by CD8^+^ T lymphocytes; however, it also destroys epitope-containing peptides. Since very few peptides have the correct amino acid sequence and length to bind to HLA class I alleles, the cleavage process does not always increase the number of HLA class I allele-binding peptides^53^. Although the exact effect of TPP2 on MHC class I antigen processing and presentation is still controversial, our data suggest a moderate, yet predominantly destructive role of TPP2.

## Methods

### Patients and tissue samples

HNSCC (127 samples), NSCLC (62 samples), and melanoma (59 samples) collected at Sun Yat-Sen Memorial Hospital, Sun Yat-Sen University (Guangzhou, China) between January 2006 and December 2010 were used for MHC-I, pSer616 DRP-1 staining, Spearman correlation, and Kaplan–Meier survival plotting. The date of death was obtained from patient records or through follow-up telephone calls. Survival time was calculated from the date of surgery to the date of death or to the last follow-up. Each patient has been followed up for at least 60 months. In addition, tumor samples and peripheral blood samples obtained from 109 patients with HNSCC and 60 patients with NSCLC at Sun Yat-Sen Memorial Hospital, Sun Yat-Sen University between April 2015 and October 2018 were used for primary cancer cell and T cell isolation and analysis. Peripheral blood (15–20 mL) was obtained from each patient. The clinical features of these patients are provided in Supplementary Tables 3 and 4. All samples were collected from patients who had provided informed consent. All related procedures were performed with the approval of the internal review and ethics board of Sun Yat-Sen Memorial Hospital and the patients gave full informed consent and signed the informed consent form.

### Cell lines

Human cancer cell lines SCC-9 (TSCC), CAL-27 (TSCC), A549 (NSCLC), Saos-2 (osteosarcoma), and mouse cancer cell line B16F10 (melanoma) were purchased from American Type Culture Collection. Human melanoma cell lines Colo38, FO-1, and M21 were provided by Dr Soldano Ferrone (Massachusetts General Hospital, Harvard Medical School). Cells were cultured in DMEM, DMEM/F12, or RPMI 1640 (Thermo Scientific) supplemented with 10% fetal bovine serum (FBS) (Gibco).

### Syngeneic models

All mouse experiments were reviewed and approved by the ethics boards and the Clinical Research Committee of Sun Yat-Sen Memorial Hospital (No. 2018000285). Female C57BL/6 mice of 5–6 weeks that were purchased from Laboratory Animal Center, Sun Yat-Sen University were used as syngeneic models, with 6 mice per experimental group. B16F10 cells stably expressing OVA were constructed by transfecting with OVA lentiviral (Cat. No. 113030, Addgene). In all, 2 × 10^5^ B16F10-OVA cells were resuspended in 150 μL PBS and then injected subcutaneously into the flanks of the C57BL/6 mice. Three days after tumor implantation, Mdivi-1 (Cat. No. HY-15886, MedChemExpress, 2.5 mg/kg) or the vehicle control (DMSO) was given by tail vein injection for 5 days in a 7-day cycle daily^22^. Tumor growth was monitored and recorded with calipers every 2 days after tumor implantation. The survival endpoint was when the tumor reached a diameter of 15 mm. Tumor volume was calculated using the formula: 0.5 × length × (width)^2^. To further investigate the effect of Mdivi-1 on tumor metastasis, 2 × 10^5^ cells were resuspended in 200 μL PBS and inoculated via tail vein, Mdivi-1 was then given the same way as described above. Male Rag1^-/-^ C57BL/6 mice (Cat. No.T004753) of 5–6 weeks were obtained from GemPharmatech, Jiangsu, China. All animals were housed with a 12-h light/dark cycle (light 6 a.m. to 6 p.m.), temperature nominally 25 °C, and humidity 50%.

### IVIS Lumina imaging

B16F10-OVA cells injected to C57BL/6 mice via tail vein were pre-transfected with lentivirus carrying luciferase reporter gene to monitor the metastatic spread of malignant cells utilizing IVIS Lumina imaging. Mice were given D-Luciferin (150 mg/kg i.p.,15 min before imaging), anesthetized (3% pentobarbital), and imaged with Xenogen IVIS Lumina system (Caliper Life Sciences). Bioluminescent flux (photons/s/cm^2^/steradian) was determined to observe tumor.

### Isolation of primary cells from tumors and peripheral blood of patients with cancer

Tumors were cut into small fragments (approximately 1 mm^3^) and incubated for 30 min with collagenase type I and III (Worthington Biochemical), in RPMI 1640 medium containing 2% FBS (5 mL/g tumor tissue) at 37 °C. The tumor pieces were transferred to a tissue digestion C-tube (Miltenyi Biotec) and further dissociated enzymatically and mechanically on a gentleMACS Dissociator (Miltenyi Biotec) to obtain a single-cell suspension. Primary cancer cells were purified with EpCAM^+^ microbeads (Cat. No. 130-061-101, Miltenyi Biotec)^24^. CD3^+^ T cells were isolated from peripheral blood with EasySep™ Human T Cell Isolation Kit (Cat. No. 17951, Stemcell) according to the manufacturer’s instructions. The isolated cancer cells were discarded after ten passages.

### Preparation of cancer cells lysate

The isolated autologous cancer cells were incubated with 0.01% EDTA-solution for 5 min, carefully detached with a cell scraper, washed twice in PBS, and resuspended at a density of 5 × 10^6^/mL in serum-free medium. The cell suspensions were frozen at −80 °C and disrupted by four freeze-thaw cycles. For the removal of crude debris, the lysate was centrifuged for 10 min at 300 g. The supernatant was collected and passed through a 0.2-μm filter. The protein concentration of the lysate was determined by commercial assay (Cat. No. 5000002, BioRad)^28^.

### Generation of DCs and tumor antigen-specific T cells

DCs were generated as previously described with slight modifications^27,54^. In brief, peripheral blood mononuclear cells were isolated from the peripheral blood of patients with HNSCC or NSCLC by SepMate™ tube for density gradient centrifugation (Cat. No. 86450, Stemcell) and were subsequently allowed to adhere in culture flasks for 1 h. The initial adherent cell fraction was harvested and cultured in DMEM containing 50 ng/mL GM-CSF, 20 ng/mL IL-4 (PeproTech), and 10% heat-inactivated FBS for 6 days. The cultures were replaced with fresh medium and cytokines every 3 days, and cell differentiation was monitored through light microscopy. DCs were matured through incubation with 100 ng/mL LPS (Sigma) and 500 U/mL IFN-γ (PeproTech) for 48 h and then pulsed for 24 h with cancer cell lysates (200 μg protein/ 1 × 10^6^cells/ mL) as prepared above. DC maturation was confirmed to be >90% pure by flow cytometric analysis for specific functional markers CD80, CD83, and CD86 (Supplementary Fig. 2D). To generate tumor-specific CTLs, we incubated the isolated CD3^+^ T cells with antigen-specific DCs (5:1) in RPMI 1640 medium supplemented with 25 U/mL IL-2 (PeproTech) and 10% heat-inactivated FBS for 5 days. The generated CTLs’ ability to kill autologous cancer cells was determined by PI and Annexin V uptake of cancer cells co-cultured, Th1 cytokine production, and residual E/T ratio. For experiments involving the IFN-γ neutralizing antibody (Cat. No. 86450, Thermo Fisher Scientific), cells were incubated for 24 h with 10 μg/mL antibody.

### Patient-derived xenograft (PDX) implantation

Primary specimens were collected from patients with HNSCC and NSCLC who underwent tumor resection at Sun Yat-Sen Memorial Hospital, Sun Yat-Sen University. Four-week-old female NOD/SCID mice purchased from Beijing Vital River Laboratory Animal Technology Co., Ltd. (Beijing, China) and maintained under pathogen-free conditions were used for PDX transplantation, with three mice per experimental group. The PDX procedure was performed as previously described^27^.

Mice were housed with a 12-h light/dark cycle (light 6 a.m. to 6 p.m.), temperature nominally 25 °C, and humidity 50%. Briefly, a small incision was made on the flank of anaesthetized NOD/SCID mice. The primary HNSCC and NSCLC samples were then minced into fragments of 2–3 mm^3^ and implanted into the subcutaneous tissue. The incision was then closed with sutures. The time from patient collection to mouse implantation ranged from 1 to 3 h. Tumor formation was monitored with calipers per week following implantation.

### Adoptive cell therapy (ACT)

CD3^+^ T cells were isolated from peripheral blood of the same patients with HNSCC or NSCLC. Mature DCs were generated and verified as described above, then incubated with autologous CD3^+^ T cells (DC/T cell ratio 1:5) for 5 days. Then, 2.5 × 10^6^ T cells and 0.5 × 10^6^ DCs were intravenously transfused into each PDX-bearing mouse via tail vein after palpable tumor formation. Mdivi-1 (2.5 mg/kg) or the vehicle control (DMSO) was given on the same day as CTL transfer by tail vein injection for 5 days daily in a 7-day cycle. Tumor growth was monitored and recorded with calipers weekly after ACT. The survival endpoint was when tumors reached a diameter of 15 mm. The dissociation of harvested PDXs was the same as primary cell isolation as described above. The tumor-infiltrated T cells (TILs) were purified with CD8 microbeads (Cat. No. 130-045-201, Miltenyi Biotech). Tumor volumes were calculated with the formula: 0.5 × length × (width)^2^.

### EpCAM and TUNEL co-staining

Frozen biopsy samples of harvested PDXs were routinely cut into 4-μm-thick sections. The sections were then mounted on glass slides and fixed with 4% paraformaldehyde for 3 min at room temperature. TUNEL staining was performed with an In Situ Cell Death Detection Kit (Cat. No. 11684817910, Roche) according to the manufacturer’s instructions. The sections were then washed twice with 0.01 mM PBS at pH 7.4, then stained with rabbit anti-human EpCAM (Cat. No. ab213500, Abcam, 1:100) overnight at 4 °C and subsequently by Alexa Fluor 594-conjugated secondary antibody (Cat. No. A-11012, Thermo Fisher Scientific) for 1 h at room temperature. DAPI was then used to counterstain the nuclei, and the images were acquired with upright fluorescence microscope (Axio Imager A2, ZEISS).

### Enzyme-linked immunospot (ELISpot)

To analyze the TILs, single-cell suspensions were generated as previously described from harvested tumors or PDXs and were rested overnight to get rid of living tumor cells via plastic adherence. Viable cells were separated via density gradient centrifugation and added to the ELISpot plate^23^. The number of IFN-γ-producing T cells was determined with IFN-γ ELISpot kit (Cat. No. 2210001 and 211001, Dakewe) according to the manufacturer’s protocol.

### Cytotoxic and coculture assays

Primary specimens were collected as described above. In all, 46 HNSCC and 28 NSCLC samples with autologous peripheral blood were used for coculture analysis. Tumor-specific CTLs, generated by incubation with tumor antigen pulsed DCs as described above, were co-cultured with the autologous cancer cells at indicated E/T ratio. Cancer cells were pretreated with Mdivi-1 at 50 μM for 72 h or left untreated before coculture. After 12 h, EpCAM^+^ cells were harvested and death was assessed by flow cytometry with apoptosis detection kit (Cat. No. 88-8007-74, eBioscience) that stains for Annexin V and PI, according to the manufacturer’s instructions. The percentages of apoptotic cells include the percentages of early (Annexin V^+^ P^–^) and late apoptotic cells (Annexin V^+^ PI^+^). Specific apoptosis was calculated as: percentage of induced apoptosis-percentage of spontaneous apoptosis)/(100%–percentage of spontaneous apoptosis) × 100% as previously described^50^. We mainly focused on late apoptosis. Supernatant was collected 24 h after coculture to measure IFN-γ, IL-2, and TNF-α release using specific ELISAs (Cat. No. ELH-IFNg-1, ELH-IL-2-1, and ELH-TNFa-1, Raybiotech). After 72 h of culture at 37 °C, adherent cancer cells and T cells were collected altogether and residual cancer cells and T cells were assessed by flow cytometric analysis based on EpCAM and CD8 expression, respectively. To determine MHC-I restriction of cancer cells lysis, the target cells were preincubated with MHC-I blocking antibody w6/32 (Cat. No. sc-32235, Santa Cruz, 10 μg/mL) for 2 h at 37 °C before coculture^28^. The result was further verified by using immunodeficient FO-1 melanoma cell line^10^.

### Mitochondrial staining

To visualize the changes in mitochondrial morphology, we planted cells onto coverslips and treated as described. Then, the cells were stained for 30 min with 0.02 μM MitoTracker Red CMXRos (Cat. No. M7512, Thermo Fisher) at 37 °C protected from light. The images were acquired with laser scanning confocal microscopy (LSM 800 with Airyscan, Zeiss).

### Measurements of oxidative stress

To evaluate the oxidative stress within cancer cells, we tested several indicators as follow: oxidation of cardiolipin, intracellular ROS, and mitochondrial membrane potential (ΔΨm)^30^. We assessed cardiolipin oxidation by using fluorescent dye NAO (Cat. No. A1372, Thermo Fisher), which binds to non-oxidized cardiolipin, but not oxidized cardiolipin. We planted cells onto coverslips and treated as described, then incubated cells with 100 nM NAO for 15 min at 37 °C protected from light. The images were acquired with laser scanning confocal microscopy (LSM 800 with Airyscan, Zeiss). Intracellular ROS production was measured by flow cytometry using the oxidation-sensitive dye DCFH-DA (Cat. No. S0033, Beyotime Biotechnology). The cells were seeded in 6-well plates and treated as described. After washing with preheated PBS for three times, the cells were incubated with DCFH-DA at 37 °C for 25 min and detached, suspended to be tested. The green fluorescence was measured using FITC channel. Similarly, mitochondrial membrane potential (ΔΨm) was measured by flow cytometry using MitoScreen (JC-1) Kit (Cat. No. 551302, BD Pharmingen) according to the manufacturer’s protocol. To investigate the effects of antioxidants and pro-oxidants on TSCCs, we used antioxidants NAC and mitoTEMPO as well as pro-oxidant BAY-87-2243 to treat cells. When adherent cells were fully attached in six-well plates, culture media was removed and replaced with assay media containing BAY-87-2243 (Bayer Pharma AG) 10 nM^14,29^; NAC (Cat. No. A7250, Sigma) 5 mM; or mitoTEMPO (Cat. No. SML0737, Sigma)^14^ 10 μM.

### In vitro antigen presentation assays

5-6 weeks old female OT-1 transgenic mice were purchased from Model Animal Research Center, Nanjing University (Nanjing, China). The OVA-specific CD8^+^ T cells were isolated from spleens of OT-1 mice by negative selection using CD8a^+^ T Cell Isolation Kit (Cat. No. 130-104-075, Miltenyi Biotec). The isolated CD8^+^ T cells were cultured in RPMI 1640 medium containing 10% FBS and 100 U/mL IL-2 with activation by anti-CD3/CD28 beads (Cat. No. 11452D, Thermo Fisher)^33^. B16F10 was stably transfected with shTpp2 provided by Genechem (Shanghai, China) and transiently transfected with OVA (Generay) or SIINFEKL (Cat. No. 102944, Addgene) with the same backbone which was GFP labeled. The activated CTLs described above were co-cultured with pretreated B16F10 cells at the indicated E/T ratio. Following a 12-h incubation, GFP^+^ cells were harvested, and cell death was assessed by flow cytometry using an apoptosis detection kit (Cat. No. 88-8007-74, eBioscience) that stains for annexin V and PI, according to the manufacturer’s instructions. Specific cell death was calculated as described above. Supernatant was harvested following a 24-h incubation to measure IFN-γ using an ELISA (Cat. No. ELM-IFNg-1, Raybiotech).

### Bioinformatic analysis

We obtained XBP-1 ChIP-seqs of T47D, HS578T, and MDA-MB-231 cells from ENCODE, then processed them by ENCODE processing pipeline. To predict the potential XBP-1 binding sites at TPP2 promoter region, we used motif-counter (https://bio.tools/motifcounter) to scan TPP2 promoter region from both strands with XBP-1 motif obtained from JASPAR database.

### Chromatin immunoprecipitation assays (ChIP)

ChIP assays were performed as previously described^55^. In short, at room temperature, the 5 × 10^6^ cells were washed with PBS and then incubated with 1% formaldehyde for 10 min. 0.1 M glycine was used to stop cross-linking for 5 min. The cells were washed twice with PBS before lysing in a lysis buffer for 1 h at 4 °C, and then sonicated into chromatin fragments. The average length of the fragments (500–800 bp) was assessed by agarose gel electrophoresis. The samples were precleared with Protein-A agarose (Roche) at 4 °C on a rocking platform for 1 h. 5 µg of specific antibodies were added and the samples were put on a rocking platform 4 °C overnight. According to the manufacturer’s protocol, the QIAquick PCR purification kit (Qiagen) was used to purify immunoprecipitated DNA. The final ChIP DNA was then used as a template in qPCR with the primers in Supplementary Table 3. ChIP-grade anti-XBP-1 antibody (Santa Cruz, sc-8015, 200 µg/0.1 mL) and anti-RNA polymerase II antibody (Abcam, ab5131, 2 µg for 25 µg of chromatin) were used in this study.

### Luciferase reporter assay

A luciferase assay was carried out as previously described with modifications^56^. Briefly, a 2000-bp DNA fragment (from TPP2 transcriptional starting site) was cloned into the pGL4.20-Basic vector upstream of the luciferase reporter gene to construct the pGL4-TPP2-wide type (-wt) or mutant (mut). The pGL4.20 derivated reporter vectors were transfected into cells to establish a stable cell line through puromycin selection for two weeks. The pRL-TK plasmid involving Renilla Luciferase was co-transfected as a control. Dual Luciferase Reporter Assay Kit (Promega) was used to measure the luciferase activities and the target effect was displayed as the luciferase activity of the reporter vector with the target sequence relative to that of the reporter vector without the target sequence.

### Flow cytometry

Cells were stained with H-2Kb-FITC (Cat. No. MHC2163, JPT), SIINFEKL-H-2Kb-FITC (Cat. No. MA5-17999, Invitrogen), anti-Mouse CD8a-APC (Cat. No. 553035, BD Pharmingen), anti-OVA-H-2K^b^ tetramer-BV421 (Cat. No. TB-5001-4, MBL), HLA-ABC-PE (Cat. No. 560168, BD Pharmingen), Perforin-BV421 (Cat. No. 563393, BD Pharmingen), Granzyme B-FITC (Cat. No. 560211, BD Pharmingen), CD8-PE-CF594 (Cat. No. 562282, BD Pharmingen), EpCAM-BV510 (Cat. No. 563181, BD Pharmingen), ITGB1-PE (Cat. No. 555443, BD Pharmingen), TfR1-APC (Cat. No. 561940, BD Pharmingen), E-Cadherin-BV421 (Cat. No. 743712, BD Pharmingen), CD80-PE (Cat. No. 560925, BD Pharmingen), CD83-BV421 (Cat. No. 562630, BD Pharmingen), and CD86-FITC (Cat. No. 560958, BD Pharmingen). For intracellular staining, cells were pretreated with the Intracellular Fixation and Permeabilization Kit (Cat. No. 88-8824, eBioscience) according to the manufacturer’s instructions. For detection of apoptosis, cells were collected and fixed in 500 μL of Annexin V binding buffer containing 5 μL Annexin V-APC and 5 μL 7-AAD (Cat. No.640930, BioLegend). Following a 20-min incubation at room temperature in the dark, 20,000 cells were analyzed using fluorescence-activated cell sorter analysis. The experiments were repeated three times. Cells were subsequently acquired using multicolor flow cytometry (BD, FACSVerse) with BD FACSDiva software (BD Biosciences). For each sample, a minimum of 10,000 events were acquired, and data were analyzed using FlowJo v10.4.0 software.

### Quantitative real-time PCR (qRT-PCR)

qRT-PCR was performed using SYBR Green Real-time PCR Master Mix (ReverTra Ace, Toyobo) and LightCycler 480 (Roche, Basel, Switzerland) according to the manufacturer’s instructions. The sequences of all the involved primers are listed in Supplementary Table 5. The relative expression levels of control were set to 1.

### Immunoblotting

Protein extracts were resolved through 8% SDS–polyacrylamide gel electrophoresis, transferred to polyvinylidene difluoride membranes (BioRad), probed with an antibody directed against MHC-I (Cat. No. sc-32235, Santa Cruz, 1:200), IRE1α (Cat. No. 3294, Cell Signaling Technology, 1:1000, pIRE1α (Cat. No. NB100-2323, Novus Biologicals, 1:1000), XBP-1 (Cat. No. ab37151, Abcam, 1:200), XBP-1s (Cat. No. 619502, BioLegend, 1:500), GRP78 (Cat. No. 3177, Cell Signaling Technology, 1:1000), CHOP (Cat. No. 2895, Cell Signaling Technology, 1:1000), DRP-1 (Cat. No. 14647, Cell Signaling Technology, 1:1000), TPP2 (Cat. No. 66017-1-Ig, Proteintech, 1:1000), GAPDH (Cat. No. 60004-1-Ig, Proteintech, 1:20,000), β-actin (Cat. No.20536-1-AP, Proteintech, 1:2000), then with a peroxidase-conjugated secondary antibody (Cat. No. SA00001-1, SA00001-2, Proteintech,1:1000). and visualized by chemiluminescence (GE).

### Immunofluorescence and immunohistochemistry staining

Antigen retrieval was performed by incubation of the slides in a pressure cooker for 5 min in 0.01 M citrate buffer, pH 6.0, and subsequent treatment with 3% hydrogen peroxide for 5 min. Slides were incubated overnight at 4 °C with antibodies as follow: HC-10+HC-A2 (targeting MHC-I, a generous gift from Dr Koichi Sakakura, Massachusetts General Hospital, Harvard Medical School), pSer616 DRP-1 (Cat. No. bs-12702R-HRP, Bioss, 1:300) and EpCAM (Cat. No. ab213500, Abcam, 1:16,000). Immunohistochemical staining was performed according to the manufacturer’s instructions. In order to simplify the analysis, expression of MHC-I and pSer616 DRP-1 were scored based on both intensity (0–3) and extent of staining (1–3). A multiplicative staining score was calculated by multiplying the intensity and extent scores to yield scores on a 9-point scale from 1 to 9. When dealing with multiple scores per patient, the individual scores were averaged to obtain a final score. Cases were divided based on staining scores into three groups: weak (0–2), moderate (3–5), and strong (6–9)^19^. For confirming the effect of Mdivi-1 treatment of mice on the various immune cell populations within tumors by immunohistochemical. The antibodies as follow: CD4 (Cat. No. 25229s, Cell Signaling Technology, 1:400), NK1.1 (Cat. No. WLH4257, Wanleibio, 1:200), CD25 (Cat. No. AF7675, Affinity, 1:200), CD206 (Cat. No. 60143-1-Ig, Proteintech, 1:400), CD20 (Cat. No.bs-0080R, BIOSS, 1:100). The following analytical components were assessed: percent immune cell stained (membrane) was calculated for each slide assessed. Immune cell staining intensity was scored as negative (0), weak (1), moderate (2), and strong positive (3). The frequency of positive cells was defined as less than 5% (0), 5–25% (1), 26–50% (2), 51–75% (3), >75% (4). The immunohistochemical score is then multiplied by the staining intensity and the staining area. For statistical analysis, scores of 0–7 were considered low expression, and scores of 8–12 were considered high expression. For IF, specimens were incubated with MHC-I (Cat. No. sc-32235, Santa Cruz, 1:200), pSer616 DRP-1 (Cat. No. 4494, Cell Signaling Technology, 1:3200), EpCAM (Cat. No. ab213500, Abcam, 1:100) and CD8 (Cat. No. ab93278, Abcam, 1:500). The accuracy of automated measurements was confirmed through independent evaluation by two pathologists. Cells stained with the indicated antibodies were counted at ×400 magnification at least ten fields per section. The stained slides were imaged using an upright fluorescence microscope (Axio Imager A2, ZEISS).

### mRNA profiles

CAL-27 stably transduced with shRNA targeting XBP-1s for mRNA profiling. The microarray utilized in this study represents a refined version of the Agilent-026652 Whole Human Genome Microarray (4 × 44 K v2).

### Statistics

All data are expressed as the mean ± standard error of mean (s.e.m.). All statistical analyses were performed in SPSS Windows version 13.0. Spearman correlation analysis was used to assess the relationship between MHC-I and pSer616 DRP-1 expression. Kaplan–Meier survival curves were plotted and log-rank tests were performed. All experiments were performed at least in triplicates and the exact numbers of independent experiments with similar results are indicated in the figure legends. All statistical analyses of experiments were performed with two-tailed Student’s *t*-tests unless otherwise stated. *p* < 0.05 was considered statistically significant.

### Reporting summary

Further information on research design is available in the Nature Research Reporting Summary linked to this article.

## Supplementary information

Supplementary Information

Description of Additional Supplementary Files

Supplementary Data 1

Supplementary Data 2

Reporting Summary

## Data Availability

The mRNA profile data generated in this study have been deposited in the Gene Expression Omnibus (GEO) database under accession code GSE135380. The immunoblotting data generated in this study are provided in the Supplementary information file. The statistical raw data are provided in Source data file. Source data are provided with this paper.

## Source data

Source Data
