# Supplementary material for: Mitochondrial fission induces immunoescape in solid tumors through decreasing MHC-I surface expression
Source: Nat Commun. 2022 Jul 6;13:3882. doi: 10.1038/s41467-022-31417-x (PMC9259736; doi:10.1038/s41467-022-31417-x)

Supplementary Information
for
Lei et al., “Mitochondrial Fission Induces Immunoescape in Solid Tumors Through Decreasing MHC-I Surface Expression”

Supplementary Table 1

Correlation among Clinicopathological Status and the Expression of MHC-I or pSer616 DRP-1 in Cancer Patients

Table S1 HNSCC (n=127, one-way ANOVA)

**Characteristics**	MHC-I (multiplicative score)		*P*	pSer616 DRP-1 (multiplicative score)		*P*
	No. of score <5	No. of score >5		No. of score <5	No. of score >5	
**Sex**			0.102			0.326
Male	36 (45.0)	44 (55.0)		46 (57.5)	34 (42.5)	
Female	29 (61.7)	18 (38.3)		22 (46.8)	25 (53.2)	
**Age**			0.741			0.921
＜50	27 (54.0)	23 (46.0)		26 (52.0)	24 (48.0)	
≥50	38 (49.4)	39 (50.6)		42 (54.5)	35 (45.5)	
**Node metastasis**			0.000			0.030
N0	13 (23.6)	42 (76.4)		36 (65.5)	19 (34.5)	
N＋	52 (72.2)	20 (27.8)		32 (44.4)	40 (55.6)	
**Clinical stage**			0.778			0.001
Ⅲ	32 (53.3)	28 (46.7)		22 (36.7)	38 (63.3)	
Ⅳ	33 (49.3)	34 (50.7)		46 (68.7)	21 (31.3)	

NSCLC (n=62, one-way ANOVA)

**Characteristics**	MHC-I (multiplicative score)		*P*	pSer616 DRP-1 (multiplicative score)		*P*
	No. of score <5	No. of score >5		No. of score <5	No. of score >5	
**Sex**			0.665			0.964
Male	25 (56.8)	19 (43.2)		23 (52.3)	21 (47.7)	

Female	12 (66.7)	6 (33.3)		10 (55.6)	8 (44.4)	
**Age**			0.809			0.937
＜50	12 (60.0)	8 (40.0)		10 (50.0)	10 (50.0)	
≥50	25 (59.5)	17 (40.5)		23 (54.8)	19 (45.2)	
**Node metastasis**			0.000			0.019
N0	8 (28.6)	20 (71.4)		20 (71.4)	8 (28.6)	
N＋	29 (85.3)	5 (14.7)		13 (38.2)	21 (61.8)	
**Clinical stage**			0.834			0.021
Ⅲ	20 (62.5)	12 (37.5)		12 (37.5)	20 (62.5)	
Ⅳ	17 (56.7)	13 (43.3)		21 (70.0)	9 (30.0)	

Melanoma (n=59, one-way ANOVA)

**Characteristics**	MHC-I (multiplicative score)		*P*	pSer616 DRP-1 (multiplicative score)		*P*
	No. of score <5	No. of score >5		No. of score <5	No. of score >5	
**Sex**			0.243			0.305
Male	20 (66.7)	10 (33.3)		12 (40.0)	18 (60.0)	
Female	14 (48.3)	15 (51.7)		7 (24.1)	22 (75.9)	
**Age**			0.152			0.878
＜50	25 (49.1)	13 (50.9)		12 (31.6)	26 (68.4)	
≥50	9 (40.0)	12 (60.0)		7 (33.3)	14 (66.7)	
**Node metastasis**			0.000			0.000
N0	15 (38.5)	24 (61.5)		6 (15.4)	33 (84.6)	
N＋	19 (95.0)	1 (5.0)		13 (65.0)	7 (35.0)	
**Clinical stage**			0.276			0.653
Ⅲ	21 (65.6)	11 (34.4)		9 (28.1)	23 (71.9)	
Ⅳ	13 (48.1)	14 (51.9)		10 (37.0)	17 (63.0)	

Supplementary Table 2

Univariate and Multivariate Analysis of Factors Associated with Overall Survival of Cancer Patients

Table S2 HNSCC (n=127, by univariate logistic regression or Cox regression analysis)

**Variables**	**No. of cases**	***HR* (95%*CI*)**	***P***
**Univariate analysis**			
**Sex**			
Male vs. Female	80/47	0.885 (0.593-1.320)	0.549
**Age (years)**			
＜50 vs. ≥50	50/77	0.916 (0.618-1.360)	0.665
**Node metastasis**			
N0 vs. N+	55/72	17.351 (9.112-33.040)	<0.001
**Clinical stage**			
Ⅲ vs. Ⅳ	60/67	11.817 (6.866-20.338)	<0.001
**MHC-I score**			
<5 vs. >5	65/62	0.398 (0.265-0.598)	<0.001
**pSer616 DRP-1 score**			
<5 vs. >5	68/59	1.617 (1.182-2.213)	0.016
**Multivariate analysis**			
**Node metastasis**			
N0 vs. N+	55/72	73.271 (22.298-240.769)	<0.001
**Clinical stage**			
Ⅲ vs. Ⅳ	60/67	115.805 (36.014-372.379)	<0.001
**MHC-I score**			
<5 vs. >5	65/62	0.024 (0.007-0.081)	<0.001
**pSer616 DRP-1 score**			
<5 vs. >5	68/59	0.055 (0.017-0.179)	<0.001

NSCLC (n=62, by univariate logistic regression or Cox regression analysis)

Variables	No. of cases	HR (95%CI)	P
**Univariate analysis**			
**Sex**			
Male vs. Female	44/18	0.767 (0.425-1.385)	0.379
**Age (years)**			
＜50 vs. ≥50	20/42	1.401 (0.775-2.534)	0.264
**Node metastasis**			
N0 vs. N+	28/34	8.029 (3.833-16.822)	<0.001
**Clinical stage**			
Ⅲ vs. Ⅳ	32/30	14.254 (6.261-32.453)	<0.001
**MHC-I score**			
<5 vs. >5	37/25	0.554 (0.311-0.986)	0.044
**pSer616 DRP-1 score**			
<5 vs. >5	33/29	1.420 (1.018-1.982)	0.021
**Multivariate analysis**			
**Node metastasis**			
N0 vs. N+	28/34	42.813 (11.631-157.592)	<0.001
**Clinical stage**			
Ⅲ vs. Ⅳ	32/30	51.703 (14.969-178.580)	<0.001
**MHC-I score**			
<5 vs. >5	37/25	0.057 (0.009-0.361)	0.001
**pSer616 DRP-1 score**			
<5 vs. >5	33/29	0.216 (0.075-0.618)	0.004

Melanoma (n=59, by univariate logistic regression or Cox regression analysis)

Variables	No. of cases	HR (95%CI)	P
**Univariate analysis**			
**Sex**			
Male vs. Female	30/29	0.875 (0.507-1.511)	0.632
**Age (years)**			
＜50 vs. ≥50	38/21	0.850 (0.479-1.508)	0.578
**Node metastasis**			
N0 vs. N+	39/20	1.919 (1.019-3.614)	0.043
**Clinical stage**			
Ⅲ vs. Ⅳ	32/27	3.173 (1.807-5.572)	<0.001
**MHC-I score**			
<5 vs. >5	34/25	0.472 (0.267-0.833)	0.010
**pSer616 DRP-1 score**			
<5 vs. >5	19/40	2.522 (1.519-4.187)	0.026
**Multivariate analysis**			
**Node metastasis**			
N0 vs. N+	39/20	14.294 (4.790-42.654)	<0.001
**Clinical stage**			
Ⅲ vs. Ⅳ	32/27	9.997 (4.354-22.957)	<0.001
**MHC-I score**			
<5 vs. >5	34/25	0.025 (0.008-0.078)	<0.001
**pSer616 DRP-1 score**			
<5 vs. >5	19/40	4.157 (2.519-6.860)	0.008

Supplementary Table 3

Primers Used in Present Study

Gene	Direction	Primer Sequence
**HLA-A**	Forward	5’-ACCCTCGTCCTGCTACTCTC-3’
	Reverse	5’-CTGTCTCCTCGTCCCAATACT-3’
**HLA-B**	Forward	5’-CAGTTCGTGAGGTTCGACAG-3’
	Reverse	5’-CAGCCGTACATGCTCTGGA-3’
**HLA-C**	Forward	5’-CCATGAGGTATTTGTGGACCG-3’
	Reverse	5’-TCTCGGACTCTCGTCGTCG-3’
**B2M**	Forward	5’-GAGGCTATCCAGCGTACTCCA-3’
	Reverse	5’-CGGCAGGCATACTCATCTTTT-3’
**XBP-1s**	Forward	5’-CTGAGTCCGCAGCAGGTG-3’
	Reverse	5’-GGCTGGTAAGGAACTGGGTC-3’
**DRP-1**	Forward	5’-CTGCCTCAAATCGTCGTAGTG-3’
	Reverse	5’-GAGGTCTCCGGGTGACAATTC-3’
**TPP2**	Forward	5’-CCGCTACCCGGAGTATGATG-3’
	Reverse	5’-GCCTGAGGGATTTGTCCAGC-3’
**THBS2**	Forward	5’-GACACGCTGGATCTCACCTAC-3’
	Reverse	5’-GAAGCTGTCTATGAGGTCGCA-3’
**TNFRSF21**	Forward	5’-ATTGGCACATACCGCCATGTT-3’
	Reverse	5’-GGCTTGTGTTGGTACAATGCTC-3’
**NLRP1**	Forward	5’-GCAGTGCTAATGCCCTGGAT-3’
	Reverse	5’-GAGCTTGGTAGAGGAGTGAGG-3’
**DCN**	Forward	5’-ATGAAGGCCACTATCATCCTCC-3’
	Reverse	5’-GTCGCGGTCATCAGGAACTT-3’
**IL18RAP**	Forward	5’-ATGCTCTGTTTGGGCTGGATA-3’
	Reverse	5’-GTGAGAGTCGATTTCTGTGGC-3’
**4-1BB**	Forward	5’-AGCTGTTACAACATAGTAGCCAC-3’
	Reverse	5’-GGACAGGGACTGCAAATCTGAT-3’
**FGF2**	Forward	5’-AGAAGAGCGACCCTCACATCA-3’
	Reverse	5’-CGGTTAGCACACACTCCTTTG-3’
**CUL3**	Forward	5’-TGTGGAGAACGTCTACAATTTGG-3’
	Reverse	5’-GCGCCTCTGTCTACGACTT-3’
**CCND1**	Forward	5’-GCTGCGAAGTGGAAACCATC-3’
	Reverse	5’-CCTCCTTCTGCACACATTTGAA-3’
**MRC2**	Forward	5’-CCGAAACCGGCTATTCAACCT-3’
	Reverse	5’-CGGTCACACTCATACATGCCC-3’
**CTSF**	Forward	5’-AGCCCAAGTCAGCCTTCAC-3’
	Reverse	5’-CGCACCATGTTATTGACAAAGAC-3’
**VTA1**	Forward	5’-CTCCCCGCACAGTTCAAGAG-3’
	Reverse	5’-AACGACAGTAATAAGCCACCAC-3’

**CTSO**	Forward	5’-GCCTTCCGGGAAAGTCTTAATAG-3’
	Reverse	5’-TCCAGTCAAATCTTAACGGCAAA-3’
**NEDD4L**	Forward	5’-GACATGGAGCATGGATGGGAA-3’
	Reverse	5’-GTTCGGCCTAAATTGTCCACT-3’
**TNFRSF6B**	Forward	5’-GTACGCGGAGTGGCAGAAA-3’
	Reverse	5’-CAGAGGACGTTGCAGTAGC-3’
**WNT4**	Forward	5’-AGGAGGAGACGTGCGAGAAA-3’
	Reverse	5’-CGAGTCCATGACTTCCAGGT-3’
**AKT2**	Forward	5’-ACCACAGTCATCGAGAGGACC-3’
	Reverse	5’-GGAGCCACACTTGTAGTCCA-3’
**ATG5**	Forward	5’-AAAGATGTGCTTCGAGATGTGT-3’
	Reverse	5’-CACTTTGTCAGTTACCAACGTCA-3’
**ATG7**	Forward	5’-CAGTTTGCCCCTTTTAGTAGTGC-3’
	Reverse	5’-CCAGCCGATACTCGTTCAGC-3’
**CDC23**	Forward	5’-CTGTCCTGTCCATAAACAGCG-3’
	Reverse	5’-GTAAGGCCCGCAATAAGCAG-3’
**PDCD6IP**	Forward	5’-ATGGCGACATTCATCTCGGTG-3’
	Reverse	5’-CGCTTGGGTAAGTCTGCTGG-3’
**MDM2**	Forward	5’-GAATCATCGGACTCAGGTACATC-3’
	Reverse	5’-TCTGTCTCACTAATTGCTCTCCT-3’
**WNT3A**	Forward	5’-AGCTACCCGATCTGGTGGTC-3’
	Reverse	5’-CAAACTCGATGTCCTCGCTAC-3’
**UBE2R2**	Forward	5’-GGACGAGTCCGACCTCTACAA-3’
	Reverse	5’-GGTGGTGAATAGGGGTAGTCAA-3’
**PARD6A**	Forward	5’-AGCATCGTCGAGGTGAAGAG-3’
	Reverse	5’-GTATAGCCAAGTAGCACGTCC-3’
**ERAP1**	Forward	5’-CCCCTCAAATGGTCCCTTGC-3’
	Reverse	5’-GAGATGCTTCAGTGCTCTGAC-3’
**GAPDH**	Forward	5’-CGCTGAGTACGTCGTGGAGTC-3’
	Reverse	5’-GCTGATGATCTTGAGGCTGTTGTC-3’
**ChIP-qPCR (TPP2-1)**	Forward	5’- TTCGAGATCCGAACCCTCCA-3’
	Reverse	5’- CTAGCAGCGCGAGGACAC-3’
**ChIP-qPCR (TPP2-2)**	Forward	5’- ATGTCCTCAGACTCCTCCCC-3’
	Reverse	5’- CAGGATGGAGGACGAGGAAG-3’
**ChIP-qPCR (TPP2 distal)**	Forward	5’-AAAGTACATCTCCCCATCTCTGG-3’
	Reverse	5’-CCCAAAAGCTATATCGAAGTTGAA-3’
**pGL4-TPP2**	Forward	5’-AACGGTACCCACTCCAGCCTGGGCCACA-3’
	Reverse	5’-ATCTCGAGTACTCCGGGTAGCGGCAGA-3’

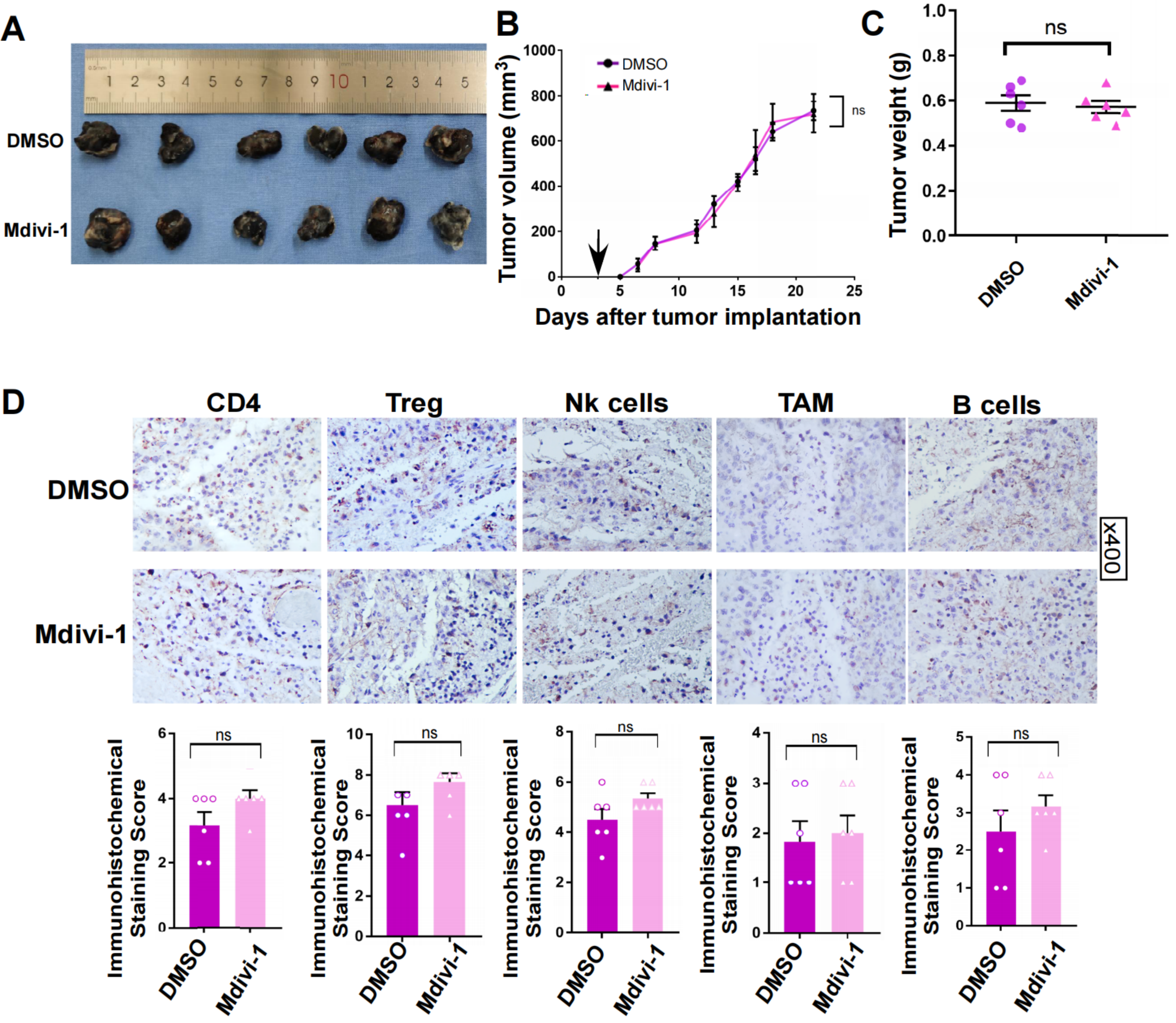
A
DMSO
Mdivi-1
B
Tumor volume (mm³)
DMSO
Mdivi-1
ns
Days after tumor implantation
C
ns
Tumor weight (g)
DMSO
Mdivi-1
D
CD4
Treg
Nk cells
TAM
B cells
DMSO
Mdivi-1
x400
Immunohistochemical Staining Score
ns

Supplementary Figure 1 **A**. Photograph of harvested tumors. **B**. Tumor volume measurements after subcutaneous implantation, arrow indicates the time of Mdivi-1 or DMSO treatment (mean ± s.e.m; n = 6, ns, $p > 0.05$ by two-way ANOVA followed by Dunnett's tests for multiple comparisons). **C**. Weights of harvested tumors (n = 6, ns, $p > 0.05$ by two-tailed t test).**D**. Representative immunochemistry images and scores for CD4+ T cells, Treg cells,

TAM, NK cells and B cells. Magnification, 400×, (n = 6, ns, $p > 0.05$ by two-tailed t test).

Supplementary Figure 2

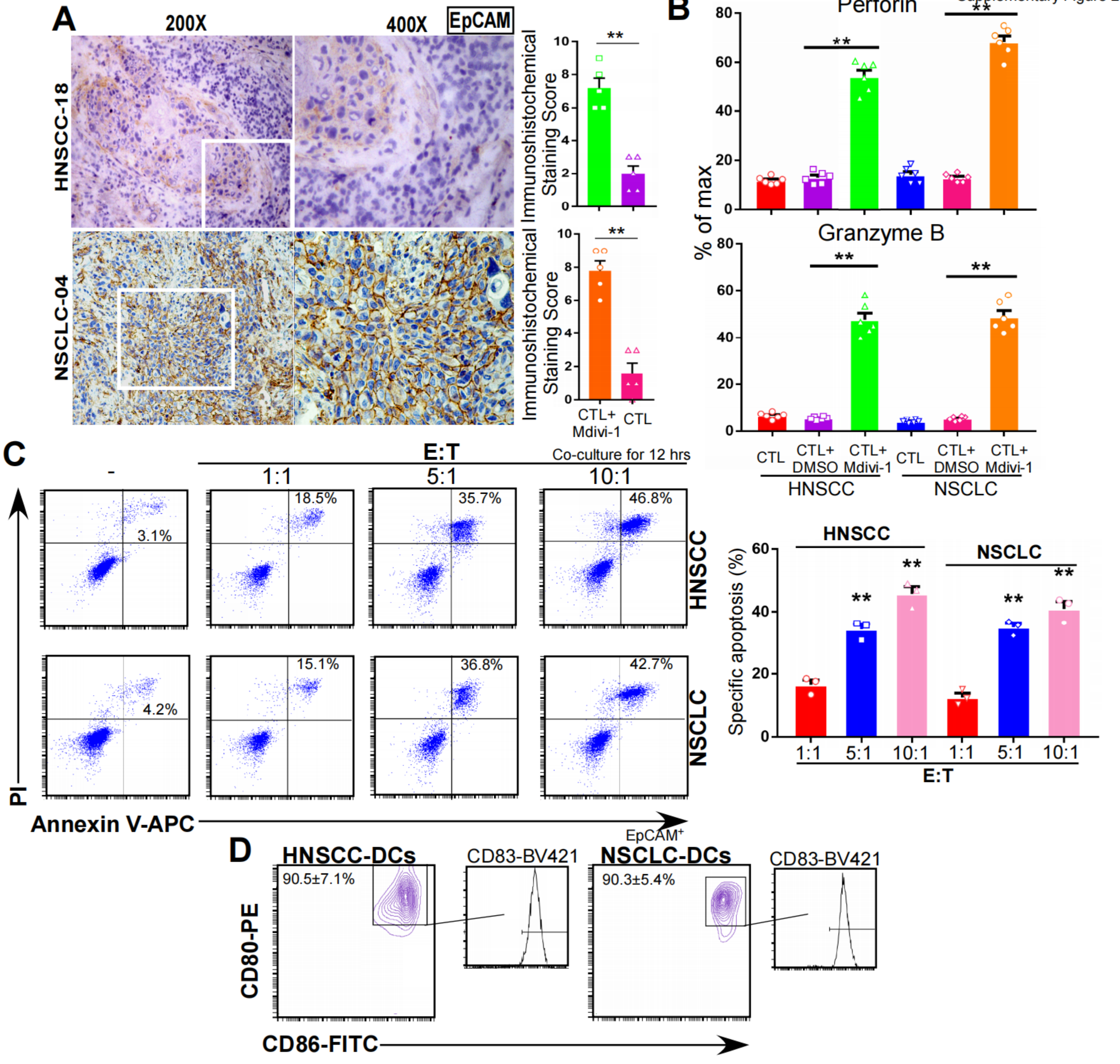

Supplementary Figure 2 A. Representative immunochemistry images for EpCAM of HNSCC and NSCLC patients. Magnification, 200× and 400×. (n = 5, $p = 0.0001$ for HNSCC-18, $p < 0.0001$ for NSCLC-04, **, $p<0.001$ by two-tailed t test). **B**. Statistical diagram of Fig 3H and I, intracellular markers staining assessed by flow cytometry (mean ± s.e.m; n = 6; $p<0.0001$ for perforin and $p<0.0001$ for granzyme B; **, $p<0.001$ by one-way ANOVA followed by Dunnett's tests for multiple comparisons). **C**. Tumor-specific CTLs were co-cultured with autologous HNSCC or NSCLC cancer cells for 12 hours at indicated E/T ratio. Death of $EpCAM^+$ cells was evaluated based on flow cytometry (mean ± s.e.m; n = 3; p = 0.0007, 0.0002, 0.0001, 0.0004; **, $p<0.001$ compared with E/T ration=1:1 group by two-tailed t test). *E* indicates effector cells namely T cells. *T* indicates targeted cells namely cancer cells. **D**. Functional markers of generated DCs assessed by flow cytometry (mean ± s.e.m; n = 7).

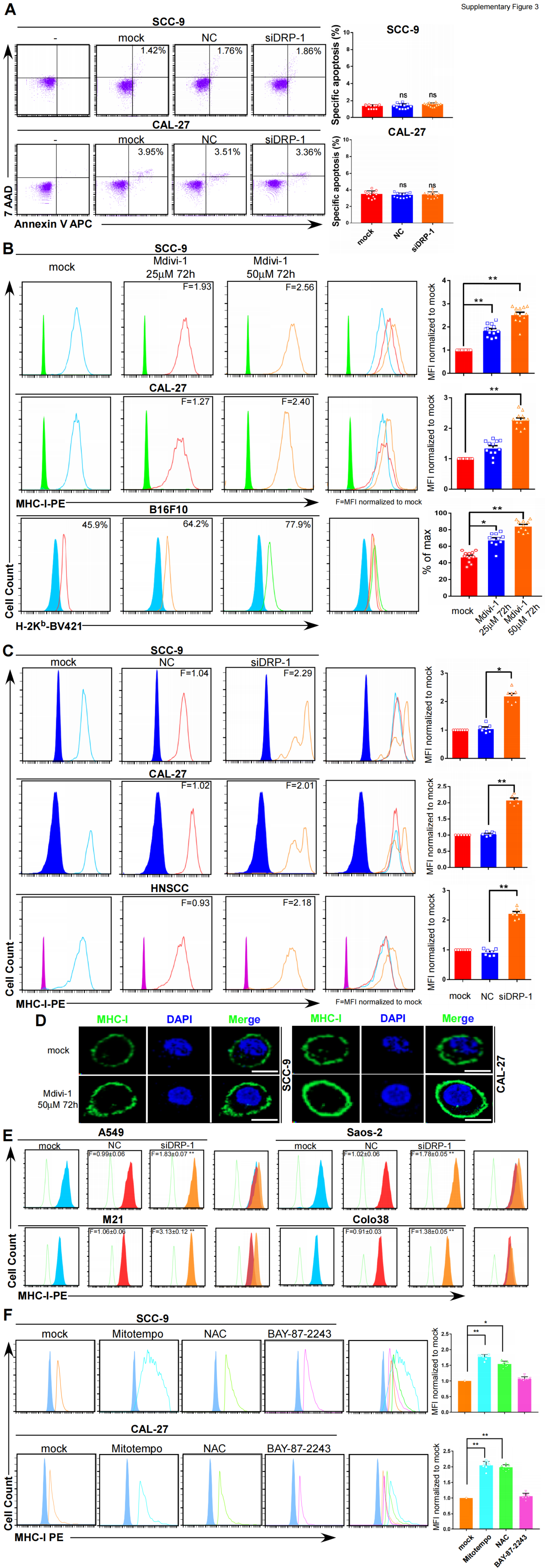
A
SCC-9
- mock NC siDRP-1
1.42% 1.76% 1.86%
CAL-27
- mock NC siDRP-1
3.95% 3.51% 3.36%
7 AAD
Annexin V APC
Specific apoptosis (%)
ns ns
mock NC siDRP-1
B
SCC-9
mock
Mdivi-1 25μM 72h
Mdivi-1 50μM 72h
F=1.93 F=2.56
MFI normalized to mock
CAL-27
F=1.27 F=2.40
MHC-I-PE
F=MFI normalized to mock
B16F10
45.9% 64.2% 77.9%
% of max
Cell Count
H-2K^b-BV421
mock Mdivi-1 25μM 72h Mdivi-1 50μM 72h
C
SCC-9
mock NC siDRP-1
F=1.04 F=2.29
CAL-27
F=1.02 F=2.01
HNSCC
F=0.93 F=2.18
Cell Count
MHC-I-PE
F=MFI normalized to mock
mock NC siDRP-1
D
MHC-I DAPI Merge
mock
Mdivi-1 50μM 72h
SCC-9
CAL-27
E
A549
mock NC siDRP-1
F=0.99±0.06 F=1.83±0.07 **
Saos-2
F=1.02±0.06 F=1.78±0.05 **
M21
F=1.06±0.06 F=3.13±0.12 **
Colo38
F=0.91±0.03 F=1.38±0.05 **
Cell Count
MHC-I-PE
F
SCC-9
mock Mitotempo NAC BAY-87-2243
CAL-27
mock Mitotempo NAC BAY-87-2243
Cell Count
MHC-I PE
MFI normalized to mock
mock Mitotempo NAC BAY-87-2243

Supplementary Figure 3 A. After silencing DRP-1 in TSCCs were harvested , cell viability and apoptosis was examined by flow cytometry based on the uptake of 7-AAD and Annexin V (mean ± s.e.m; n = 12; p=0.0509 for SCC-9, p=0.9137 for CAL-27; compared with NC by two-tailed t test). **B**. MHC-I membrane expression in TSCC and mouse melanoma cell lines was significantly upregulated through Mdivi-1 treatment as evaluated by flow cytometry. *F* indicates the fold change of MFI normalized to mock (mean ± s.e.m; n = 11; p <0.0001 for SCC-9, CAL-27 and B16F10; **, p<0.001; *, p<0.01, **, p<0.001 by two-tailed t test). **C**. MHC-I membrane expression in TSCCs and HNSCC primary cancer cells evaluated by flow cytometry. *F* indicates the fold change of MFI normalized to mock (mean ± s.e.m; n = 7; p <0.0001 for SCC-9, CAL-27 and HNSCC; **, p<0.001 by two-tailed t test). **D.** Further assessment of MHC-I expression on TSCCs was done through immunofluorescence staining (MHC-I: green). DAPI, nuclear staining. Scale bars, 5 μm. **E**. Analysis of MHC-I by flow cytometry was extended by using additional solid tumor cell lines: A549, Saos-2, M21 and Colo38 (mean ± s.e.m; n = 4; p = 0.7067, p<0.0001 for A549, p = 0.1651, p<0.0001 for Saos-2, p = 0.5322, p<0.0001 for M21, p = 0.1789, p<0.0001 for Colo38; **, p<0.001 compared with NC). NC indicates negative control. **F**. MHC-I membrane expression in TSCC cell lines was significantly changed through antioxidant and pro-oxidant treatment as evaluated by flow cytometry. *F indicates the fold change of MFI normalized to mock* (mean ± s.e.m; n = 7; p = 0.0001, 0.0001, 0.7053 for SCC-9, p = 0.0001, 0.0001, 0.9395 for CAL-27; *, p<0.05, **, p<0.001 by two-tailed t test).

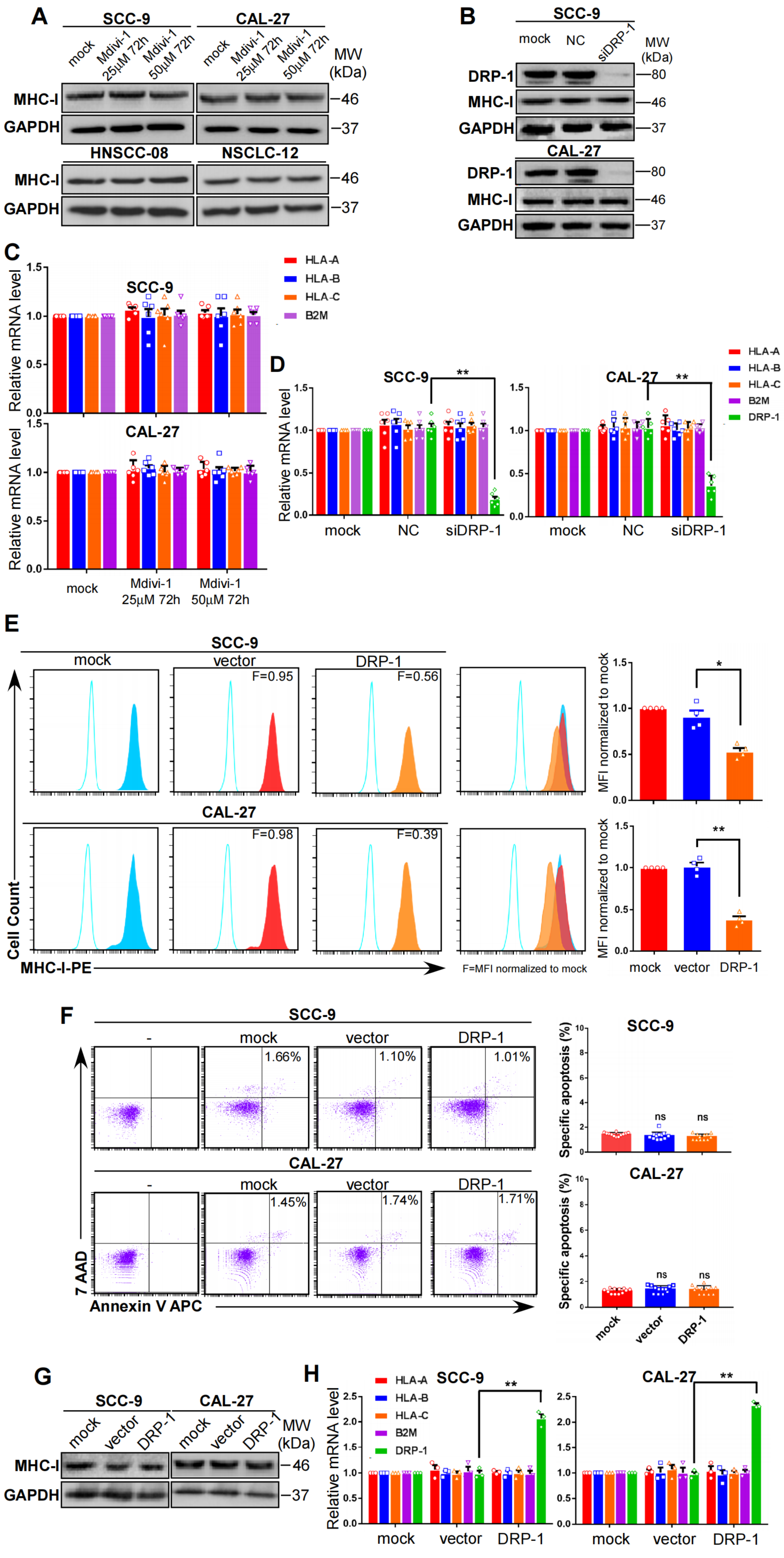
A
SCC-9
CAL-27
mock
Mdivi-1 25μM 72h
Mdivi-1 50μM 72h
MW (kDa)
MHC-I
GAPDH
46
37
HNSCC-08
NSCLC-12
B
SCC-9
mock
NC
siDRP-1
DRP-1
80
CAL-27
C
Relative mRNA level
HLA-A
HLA-B
HLA-C
B2M
D
DRP-1
E
vector
F=0.95
F=0.56
F=0.98
F=0.39
Cell Count
MHC-I-PE
F=MFI normalized to mock
MFI normalized to mock
F
1.66%
1.10%
1.01%
1.45%
1.74%
1.71%
7 AAD
Annexin V APC
Specific apoptosis (%)
ns
G
H

Supplementary Figure 4 A, B. Immunoblotting results revealed that Mdivi-1 treatment or DRP-1 knockdown did not affect MHC-I translation in TSCCs, HNSCC and NSCLC primary cancer cells. MW, molecular weight; GAPDH, loading control. **C**. qRT-PCR evaluated the relative mRNA levels of HLA-A/B/C and B2M with Mdivi-1 treatments in TSCCs (mean ± s.e.m; n = 6; p = 0.4291, 0.7805, 0.9954, 0.9979, 0.9872, 0.9041, 0.9498, 0.9676 for SCC-9 and 0.7856, 0.5845, 0.4163, 0.9238, 0.9988, 0.9238, 0.9238, 0.9988 for CAL-27 by two-tailed t test compared with mock). **D**. The relative levels of HLA-A/B/C and B2M mRNA with indicated siDRP-1 treatments in TSCCs are evaluated by qRT-PCR (mean ± s.e.m; n = 6; p <0.0001 for SCC-9 and p <0.0001 for CAL-27; *, p<0.01, **, p<0.001 by two-tailed t test compared with mock). **E**. Flow cytometry of membrane MHC-I with overexpression of MHC-I in TSCCs. *F* indicates MFI normalized to mock (mean ± s.e.m; n = 4; p = 0.0013 for SCC-9 and p <0.0001 for CAL-27; *, p<0.01, **, p<0.001 by two-tailed t test compared with mock).**F**. After overexpression of DRP-1 in TSCCs were harvested cell viability and apoptosis was examined by flow cytometry based on the uptake of 7-AAD and Annexin V (mean ± s.e.m; n= 12; p = 0.2505, 0.4749 for SCC-9 and 0.5201, 0.7437 for CAL-27 compared with vector by two-tailed t test compared with mock). **G**. Immunoblotting results revealed that overexpression of DRP-1 didn't affect MHC-I translation in TSCCs. MW indicates molecular weight. GAPDH, loading control. **H** qRT-PCR evaluated the relative mRNA levels of HLA-A/B/C and B2M with indicated treatments in TSCCs (mean ± s.e.m; n = 3; p <0.0001 for SCC-9 and CAL-27; **,p<0.001 by two-tailed t test compared with mock).

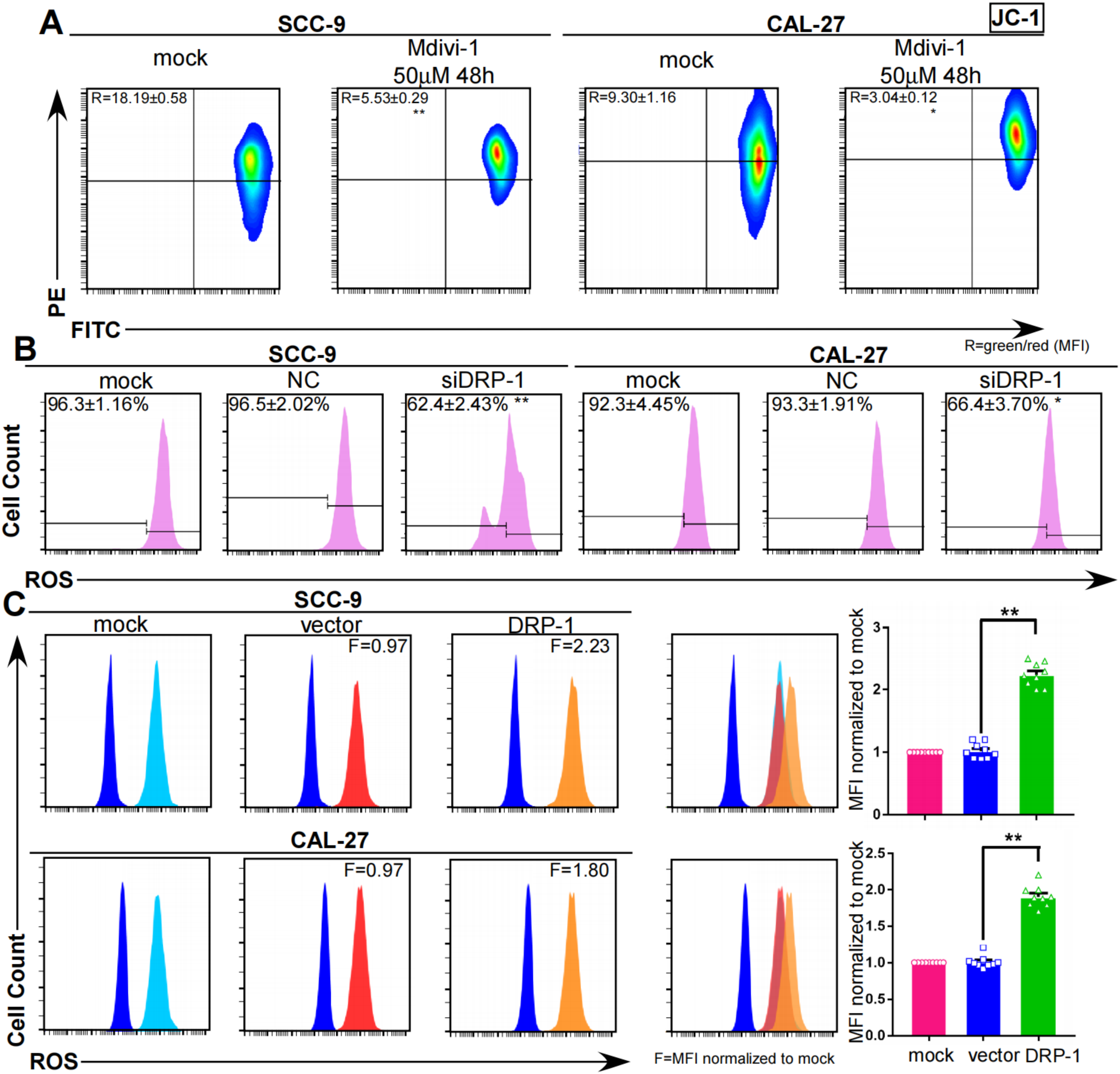
A
SCC-9
CAL-27
JC-1
mock
Mdivi-1
50μM 48h
R=18.19±0.58
R=5.53±0.29
**
R=9.30±1.16
R=3.04±0.12
*
PE
FITC
R=green/red (MFI)
B
SCC-9
CAL-27
mock
NC
siDRP-1
96.3±1.16%
96.5±2.02%
62.4±2.43% **
92.3±4.45%
93.3±1.91%
66.4±3.70% *
Cell Count
ROS
C
SCC-9
mock
vector
DRP-1
F=0.97
F=2.23
CAL-27
F=0.97
F=1.80
Cell Count
ROS
F=MFI normalized to mock
MFI normalized to mock
**
mock
vector
DRP-1

Supplementary Figure 5 A. Mitochondrial membrane potential (ΔΨm) was assessed by flow cytometry using JC-1 dye in TSCCs. R was calculated as: FITC MFI/PE MFI (mean ± s.e.m; n = 5; *p*<0.0001 for SCC-9 and *p*=0.0029 for CAL-27; *, *p*<0.01, **, *p*<0.001). **B-C**. Intracellular ROS measured by flow cytometry with indicated treatments in TSCCs (mean ± s.e.m; n = 9 ; the upper panel, *p*=0.0002 for SCC-9, 0.0015 for CAL-27, the lower panel *p*<0.0001 for SCC-9 and CAL-27 ; *, *p*<0.01, **, *p*<0.001 compared with NC or vector). F indicates MFI normalized to mock. NC indicates negative control.

Supplementary Figure 6

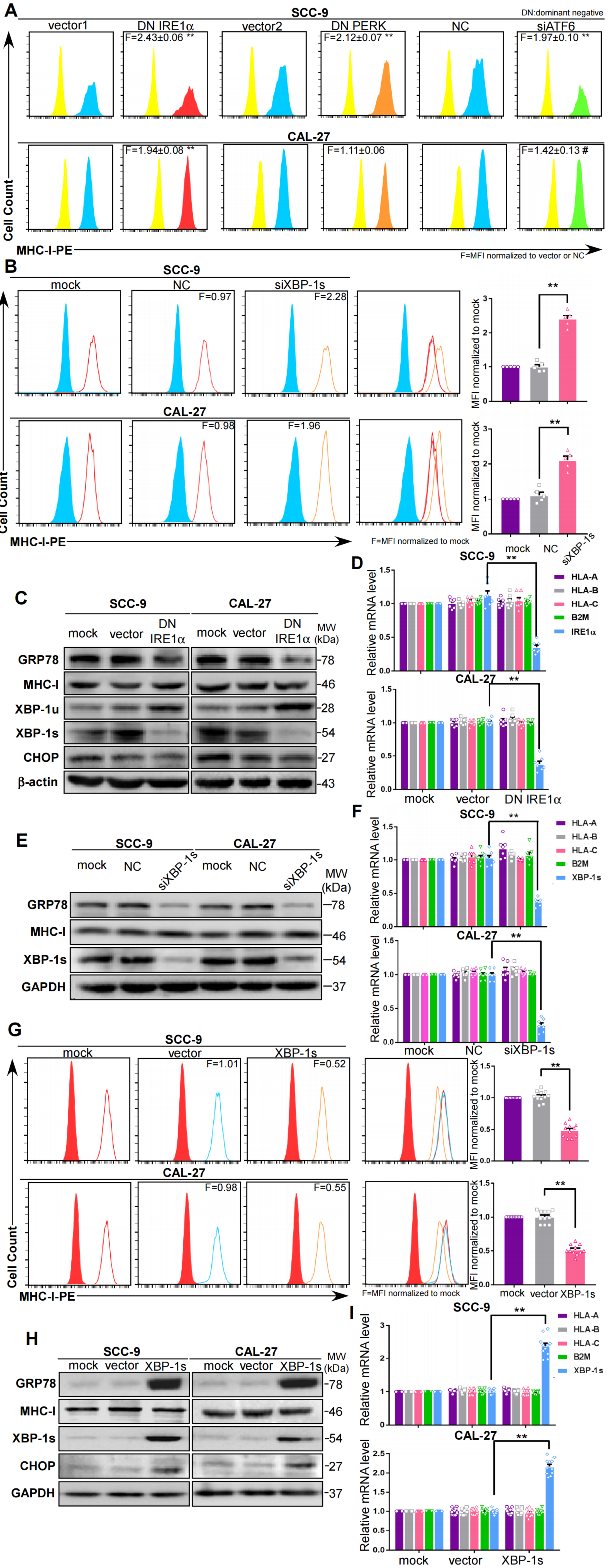

Supplementary Figure 6 A. Assessment of MHC-I membrane expression by flow cytometry in TSCCs with indicated treatments (mean ± s.e.m; n = 3; *p*<0.0001, *p*<0.0001, 0.0006 for SCC-9 and p=0.0002, 0.1583 ,0.0297 for CAL-27; #, *p*<0.05, **, *p*<0.001 compared with corresponding vector or NC). F indicates MFI normalized to vector or NC. NC indicates negative control. **B**. Assessment of MHC-I membrane expression in TSCCs by flow cytometry after XBP-1s knockdown. F indicates the fold change of MFI normalized to mock (mean ± s.e.m; n = *5; p*<0.0001 for SCC-9 and CAL-27; **, *p*<0.001 by two-tailed t test compared with mock). **C**. Immunoblotting results revealed that ER stress was attenuated while MHC-I translation was unaffected when TSCCs were transfected with DN IRE1α. MW indicates molecular weight. β-actin, loading control. **D**. qRT-PCR showed unaffected HLA-A/B/C and B2M transcription in TSCCs with DN IRE1α (mean ± s.e.m; n = 7; *p*<0.0001 for SCC-9 and CAL-27; **, *p*<0.001 by two-tailed t test compared with mock). **E**. Immunoblotting showed that the knockdown of XBP-1s attenuated ER Stress in TSCCs as indicated by associated makers GRP78 and XBP-1s, whereas MHC-I was not affected. MW indicates molecular weight. GAPDH, loading control. **F**. qRT-PCR demonstrated that the transcription of HLA-A/B/C and B2M were not affected by the knockdown of XBP-1s in TSCCs (mean ± s.e.m; n = 7; *p*<0.0001 for SCC-9 and CAL-27; **, *p*<0.001 by two-tailed t test compared with mock). **G**. Assessment of MHC-I membrane expression by flow cytometry in TSCCs when XBP-1s was overexpressed (mean ± s.e.m; n = 11; *p*<0.0001 for SCC-9 and *p*=0.005 for CAL-27 *, *p*<0.01, **, *p*<0.001 by two-tailed t test compared with mock). F indicates MFI normalized to mock. **H**. Immunoblotting results showed that ER stress was exacerbated while MHC-I translation remained unaffected when XBP-1s was overexpressed in TSCCs. MW indicates molecular weight. GAPDH, loading control. **I**. qRT-PCR revealed that HLA-A/B/C and B2M transcription were unaffected in TSCCs with indicated treatment (mean ± s.e.m; n = 11; *p*<0.0001 for SCC-9 and CAL-27; **, *p*<0.001 by two-tailed t test compared with mock).

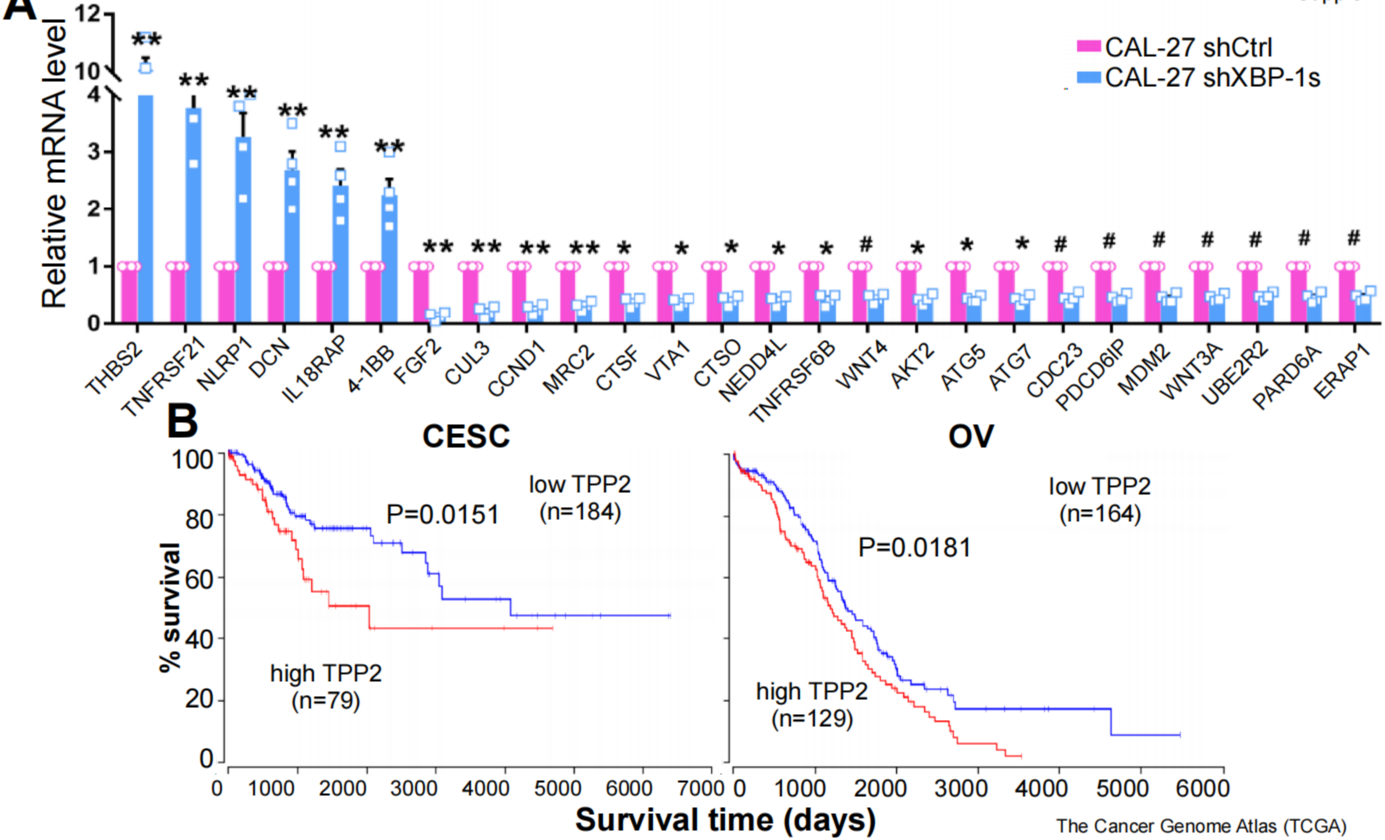
A
Relative mRNA level
CAL-27 shCtrl
CAL-27 shXBP-1s
THBS2
TNFRSF21
NLRP1
DCN
IL18RAP
4-1BB
FGF2
CUL3
CCND1
MRC2
CTSF
VTA1
CTSO
NEDD4L
TNFRSF6B
WNT4
AKT2
ATG5
ATG7
CDC23
PDCD6IP
MDM2
WNT3A
UBE2R2
PARD6A
ERAP1
B
CESC
OV
% survival
P=0.0151
low TPP2
(n=184)
high TPP2
(n=79)
P=0.0181
low TPP2
(n=164)
high TPP2
(n=129)
Survival time (days)
The Cancer Genome Atlas (TCGA)

Supplementary Figure 7 A. qRT-PCR verified the downregulation of genes from microarrays in CAL-27 after XBP-1s knockdown (mean ± s.e.m; n = 4; p<0.0001, p<0.0001, p<0.0001, p<0.0001, p<0.0001, p<0.0001, p<0.0001, p<0.0001, p=0.0001, 0.0006, 0.0028, 0.0028, 0.0051, 0.0047, 0.0073, 0.0121, 0.0081, 0.0085, 0.0072, 0.0133, 0.0125, 0.0128, 0.0154, 0.024, 0.0159, 0.0211; #, p<0.05, *, p<0.01, **, p<0.001 by two-tailed t test compared with shCtrl). **B**. Overall survival of cancer patients with different levels of TPP2 using The Cancer Genome Atlas (TCGA) database, in addition to the cancer types that were experimented in this project. *CESC: Cervical squamous cell carcinoma and endocervical adenocarcinoma. OV: Ovarian serous cystadenocarcinoma.*

Raw Data Related to Figure 5E

Same membranes are indicated by a black dashed box, and the cropped area is indicated by a red box. Membrane was cut.

anti-IRE1a

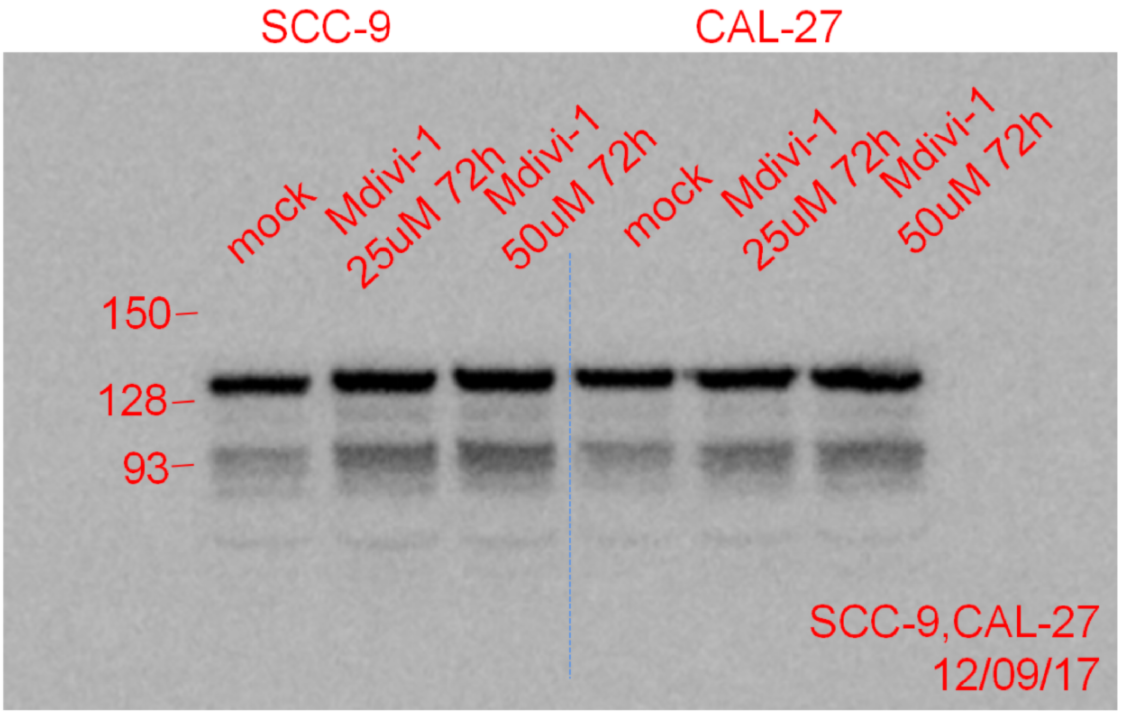

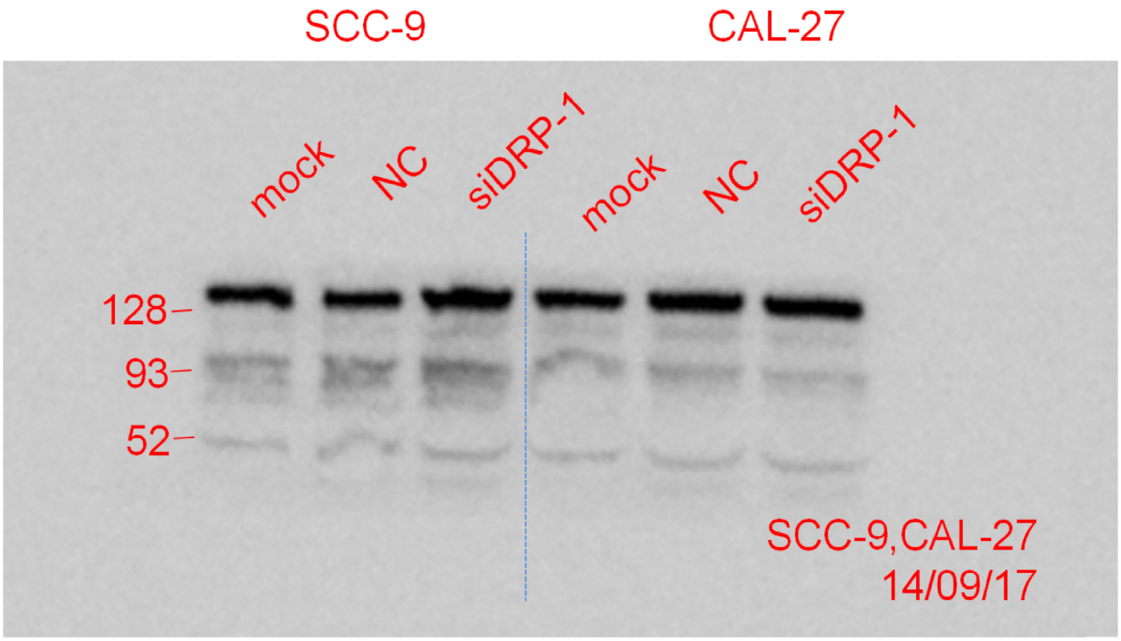

Raw Data Related to Figure 5E

Same membranes are indicated by a black dashed box, and the cropped area is indicated by a red box. Membrane was cut.

anti-pIRE1a

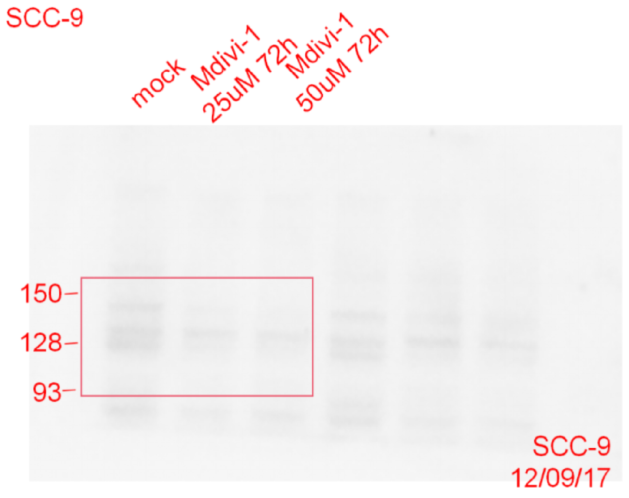

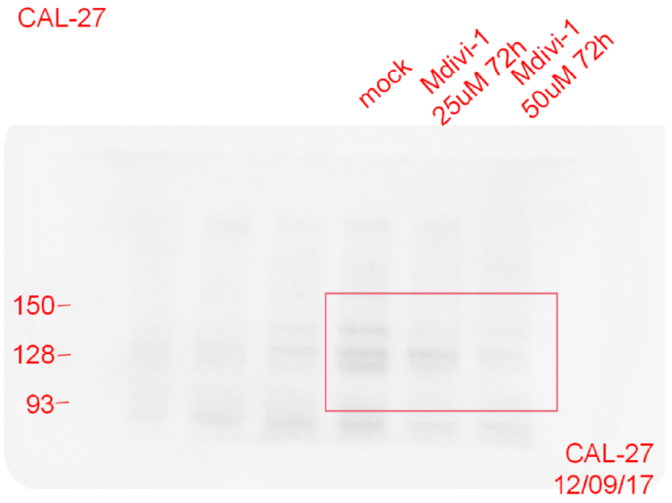

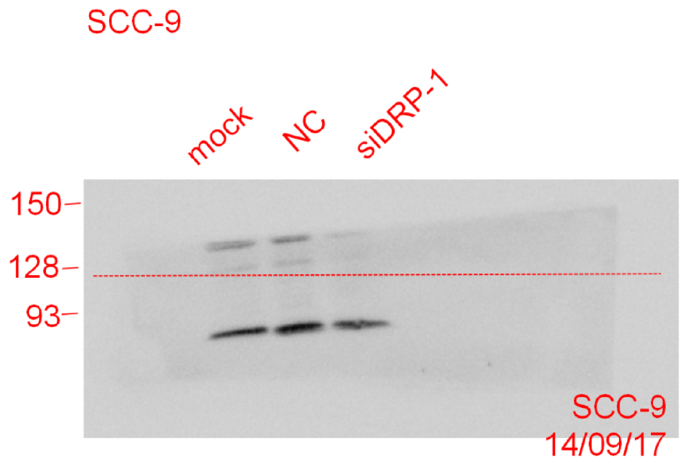

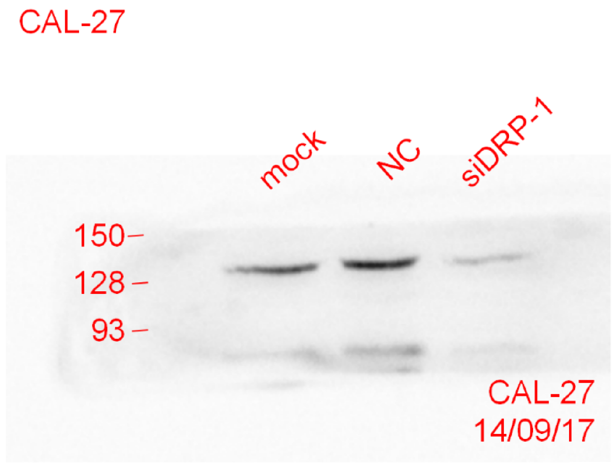

Raw Data Related to Figure 5E

Same membranes are indicated by a black dashed box, and the cropped area is indicated by a red box. Membrane was cut.

anti-XBP-1u

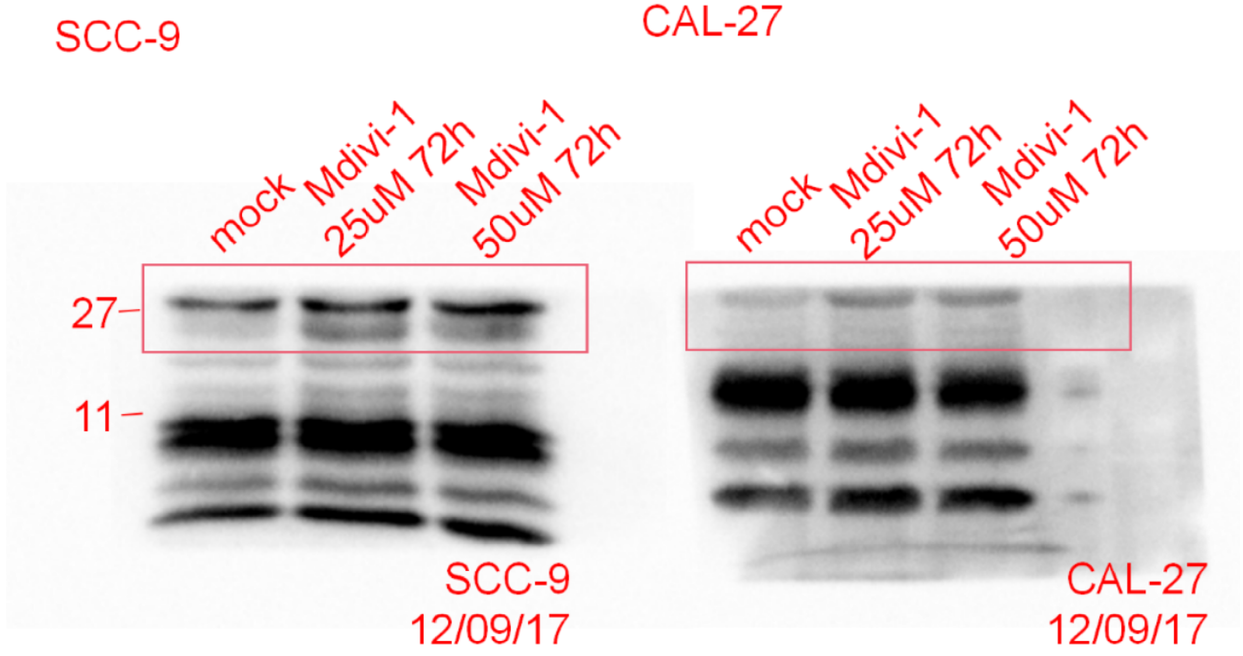

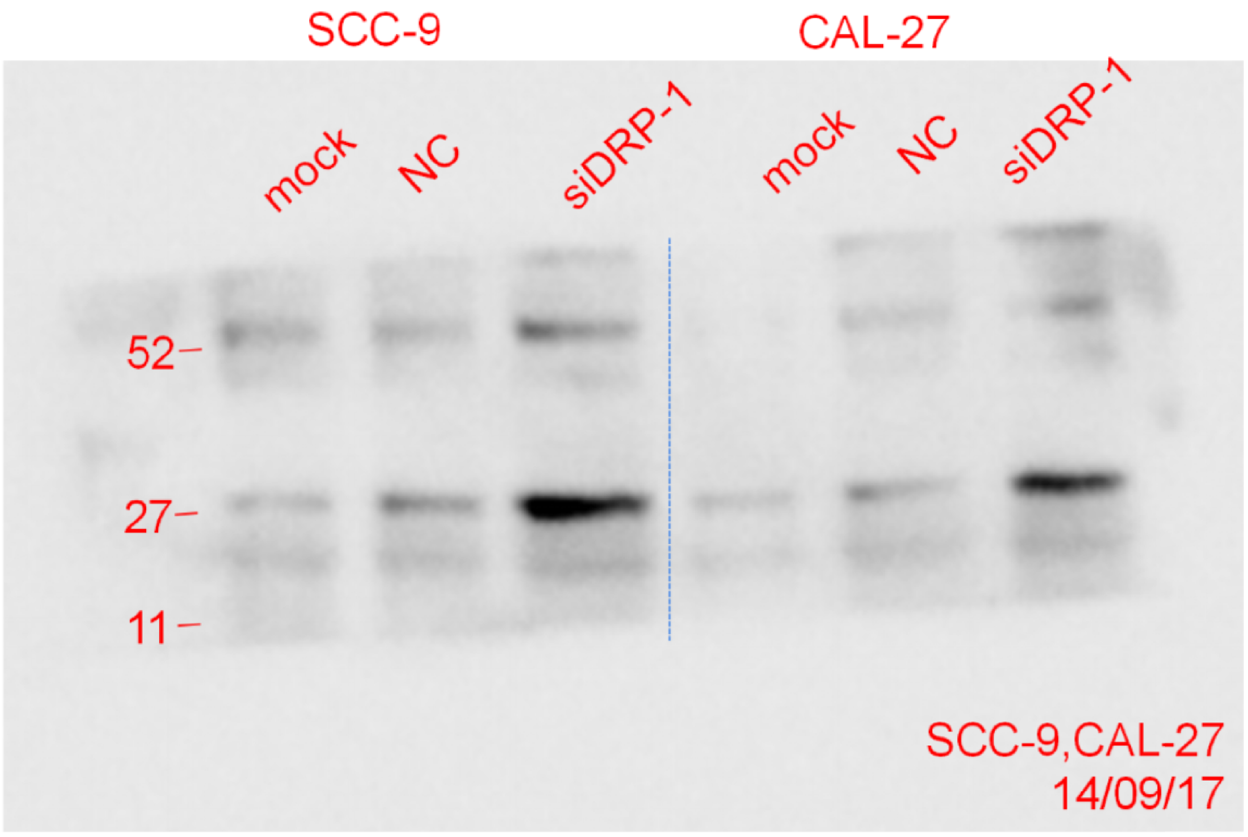

Raw Data Related to Figure 5E

Same membranes are indicated by a black dashed box, and the cropped area is indicated by a red box. Membrane was cut.

anti-XBP-1s

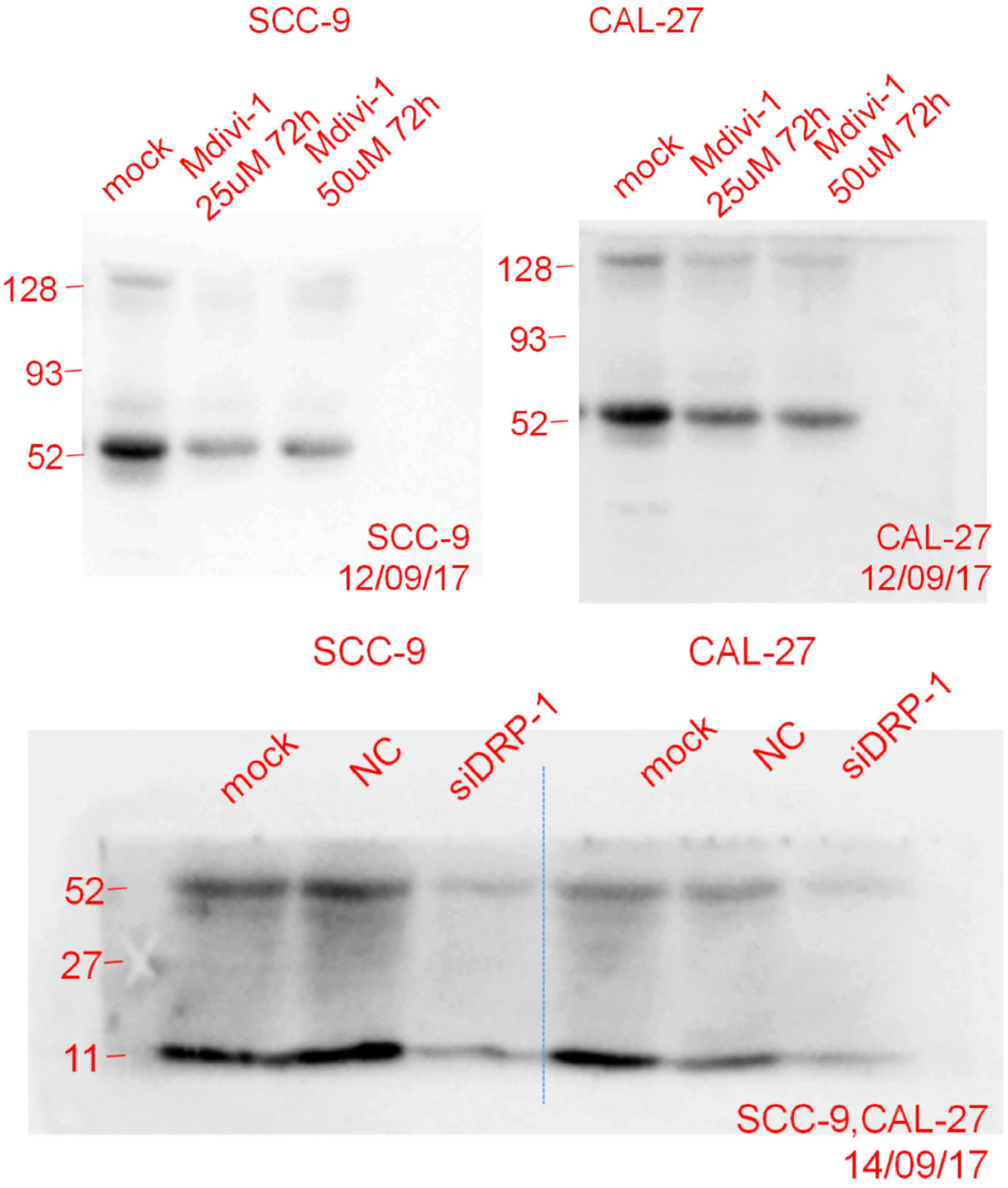

Raw Data Related to Figure 5E

Same membranes are indicated by a black dashed box, and the cropped area is indicated by a red box. Membrane was cut.

anti-GAPDH

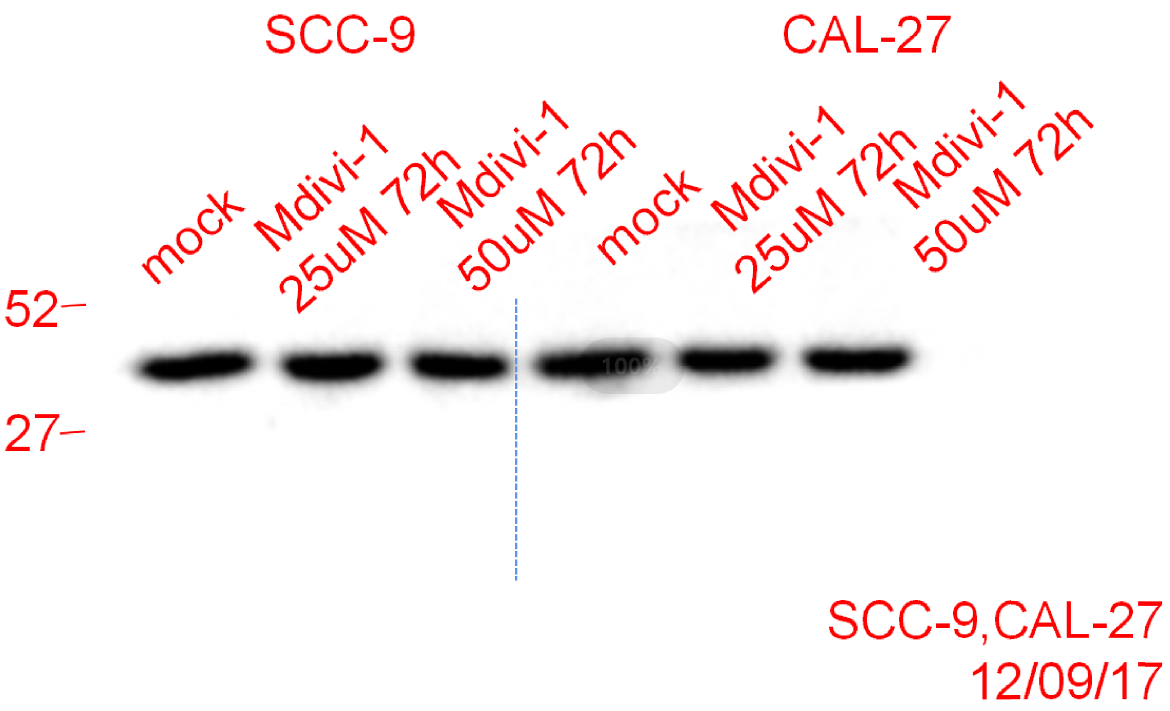

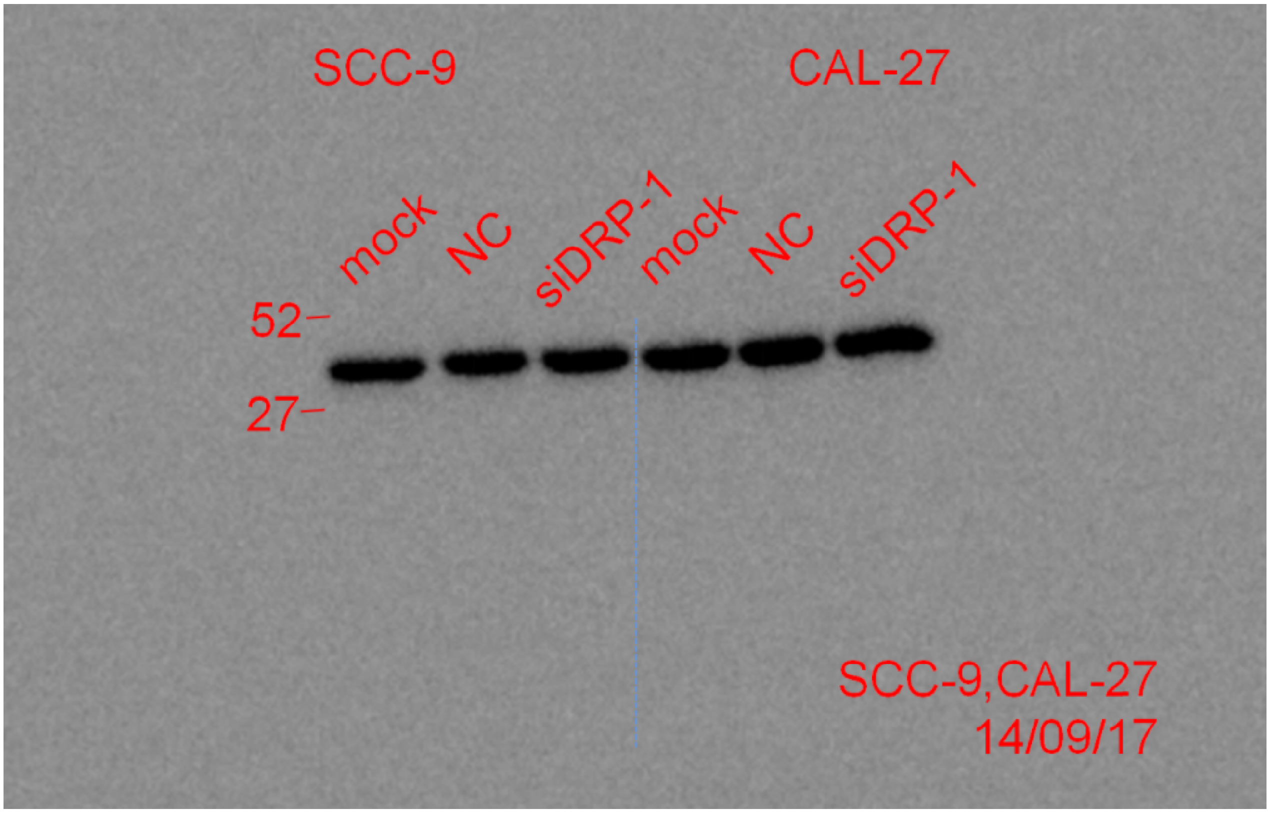

Raw Data Related to Figure 6N

Same membranes are indicated by a black dashed box, and the cropped area is indicated by a red box. Membrane was cut.

anti-TPP2

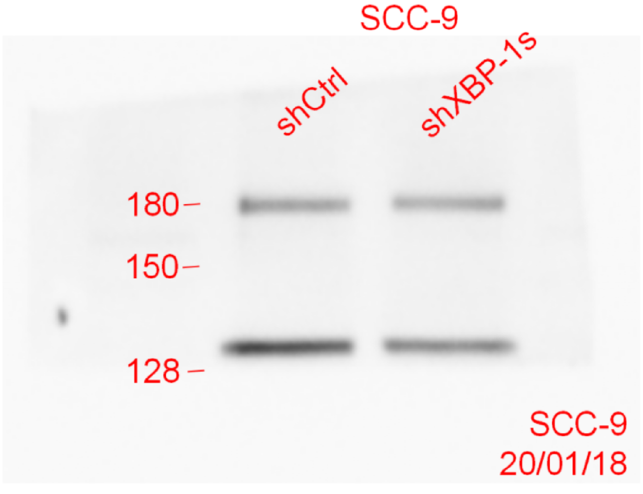

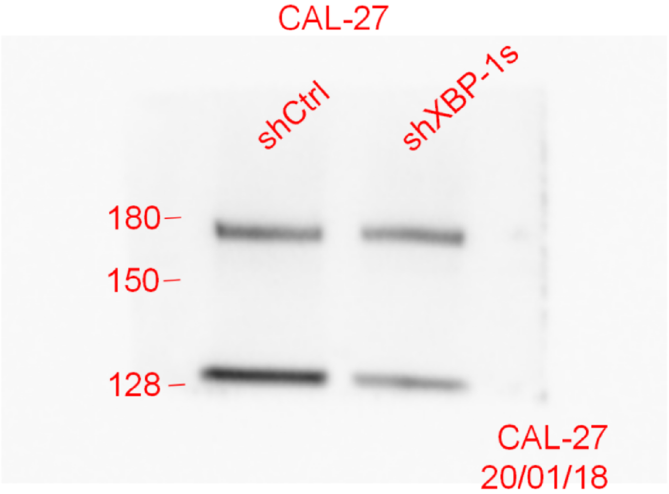

anti-beta-actin

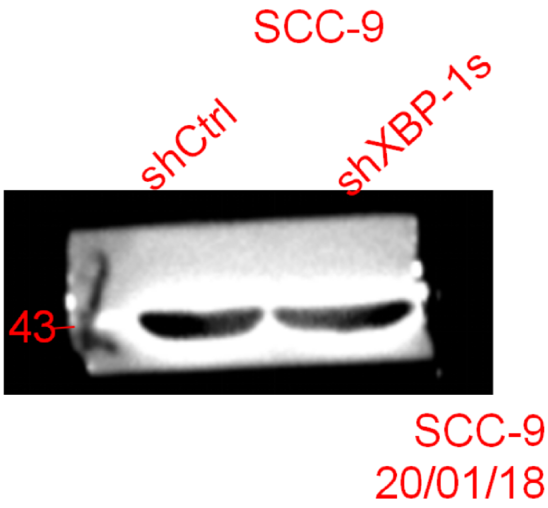

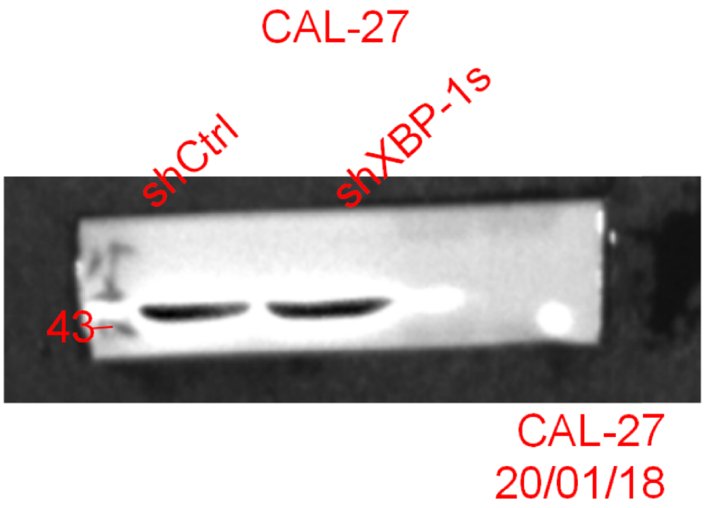

Raw Data Related to Supplementary Figure 4A

Same membranes are indicated by a black dashed box, and the cropped area is indicated by a red box. Membrane was cut.

anti-MHC-I

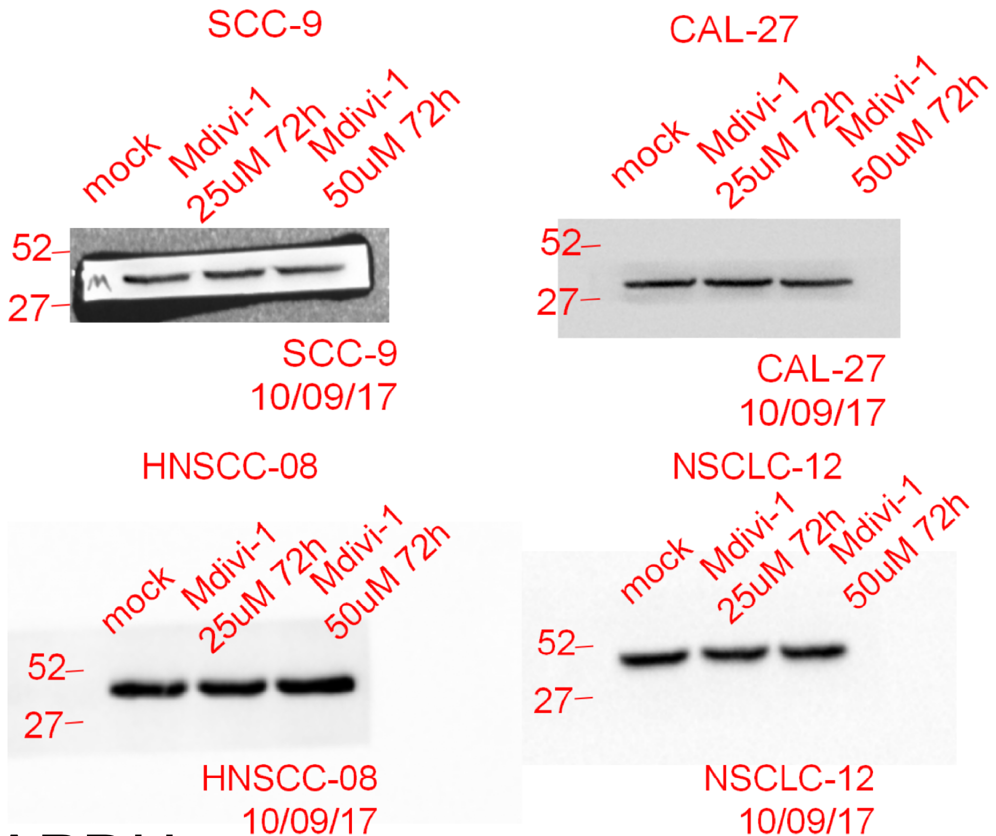

anti-GAPDH

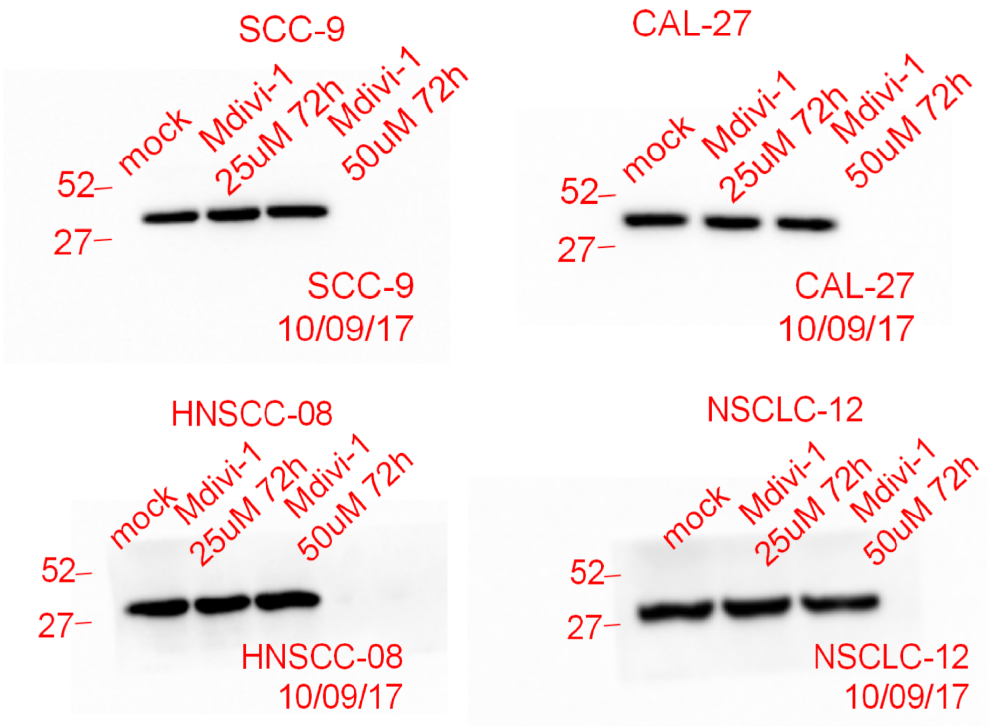

Raw Data Related to Supplementary Figure 4B

Same membranes are indicated by a black dashed box, and the cropped area is indicated by a red box. Membrane was cut.

anti-MHC-I

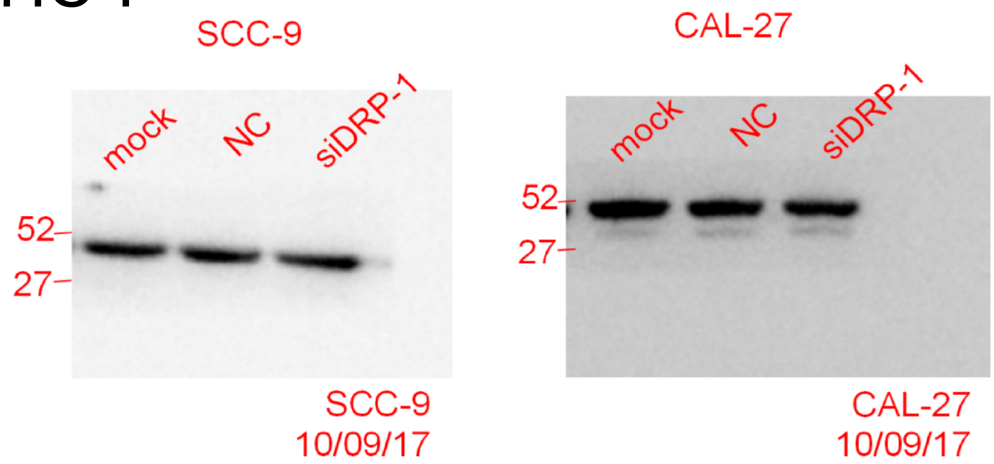

anti-DRP-1

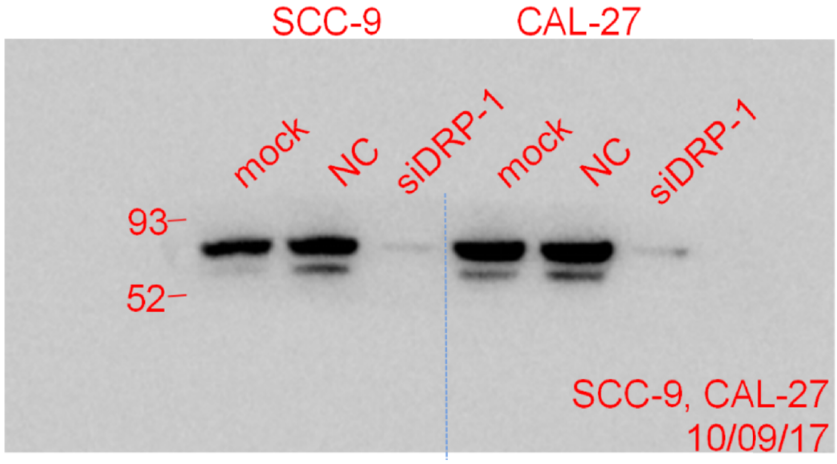

anti-GAPDH

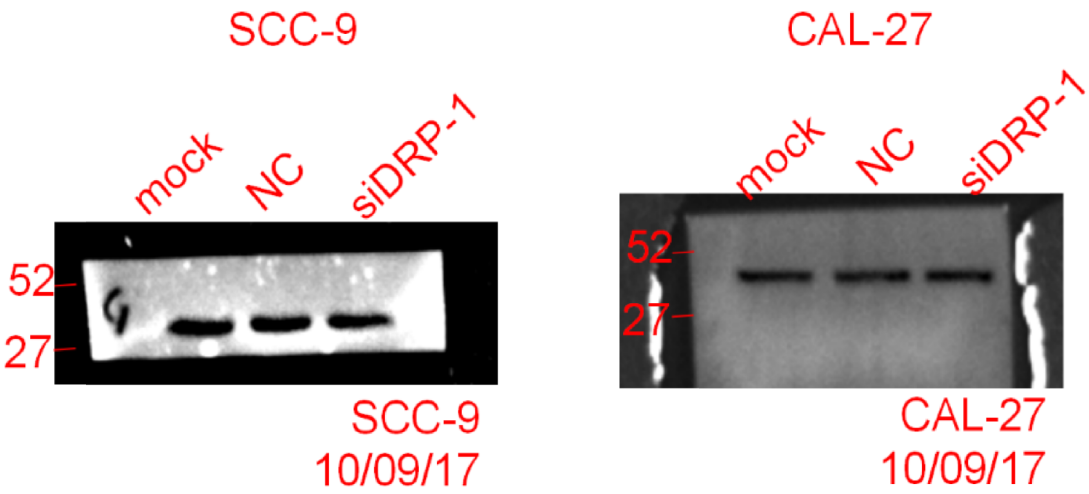

Raw Data Related to Supplementary Figure 4G

Same membranes are indicated by a black dashed box, and the cropped area is indicated by a red box. Membrane was cut.

anti-MHC-I

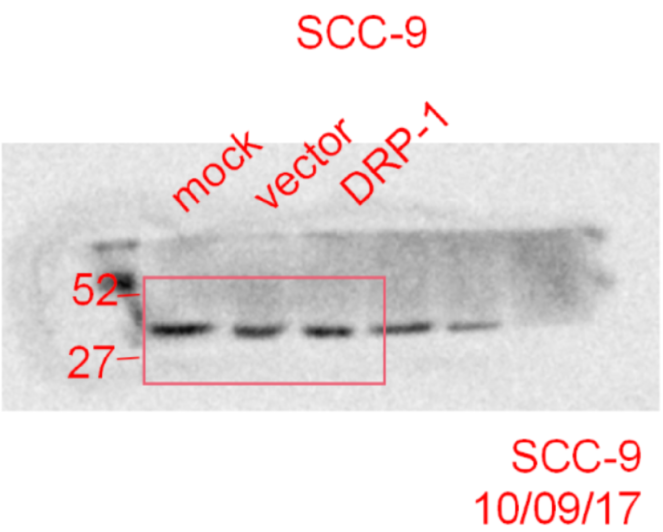

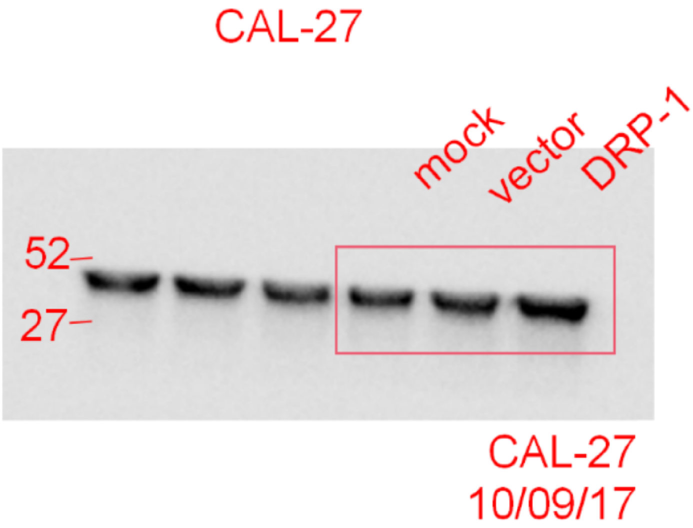

anti-GAPDH

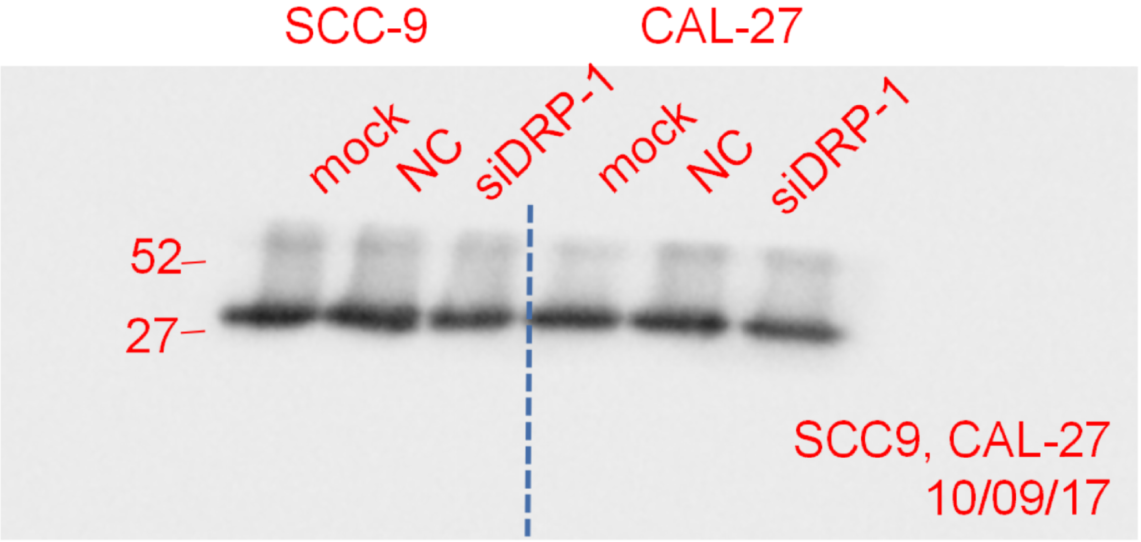

Raw Data Related to Supplementary Figure 6C

Same membranes are indicated by a black dashed box, and the cropped area is indicated by a red box. Membrane was cut.

anti-GRP78

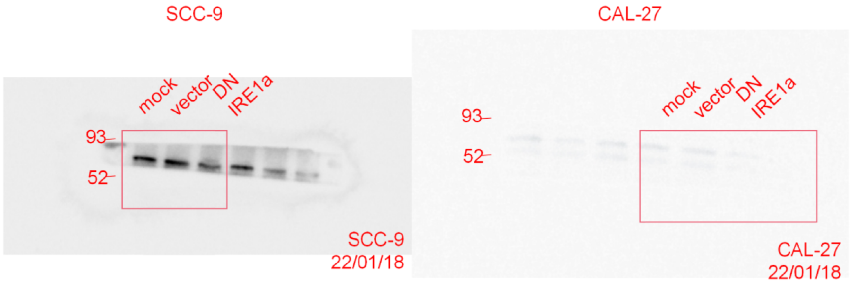

anti-MHC-I

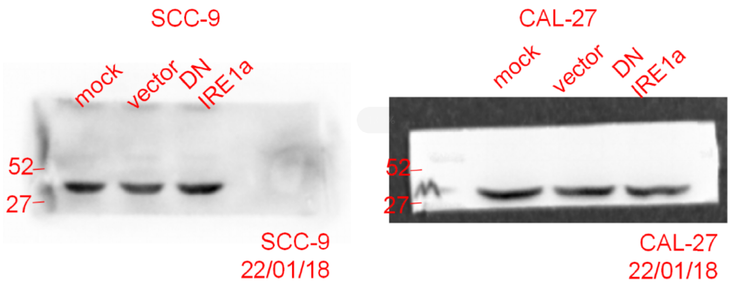

anti-XBP-1u

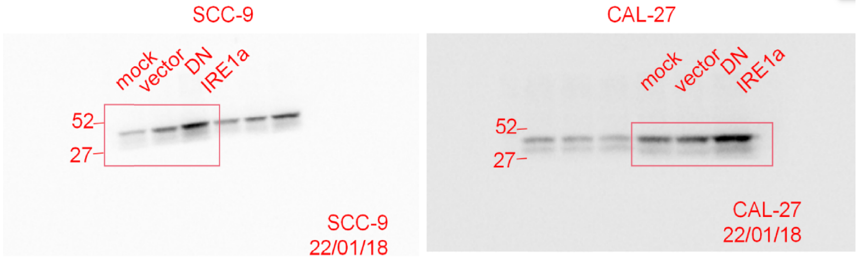

anti-XBP-1s

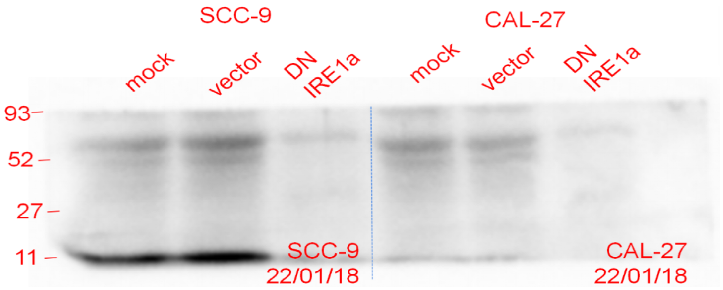

anti-CHOP

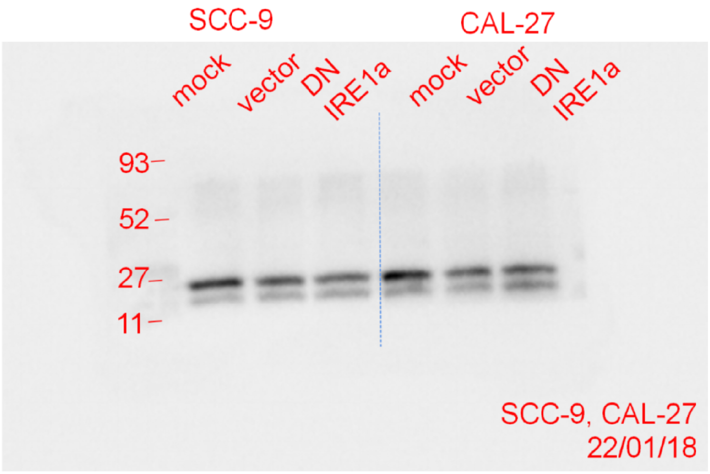

anti-beta-actin

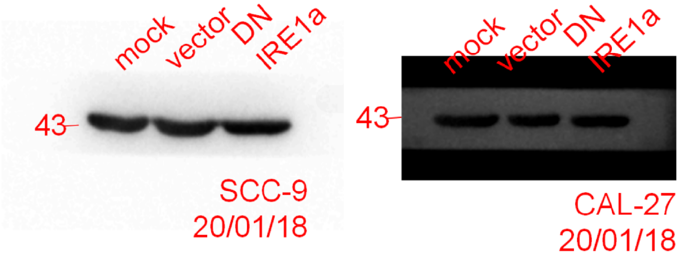

Raw Data Related to Supplementary Figure 6E

Same membranes are indicated by a black dashed box, and the cropped area is indicated by a red box. Membrane was cut.

anti-GRP78

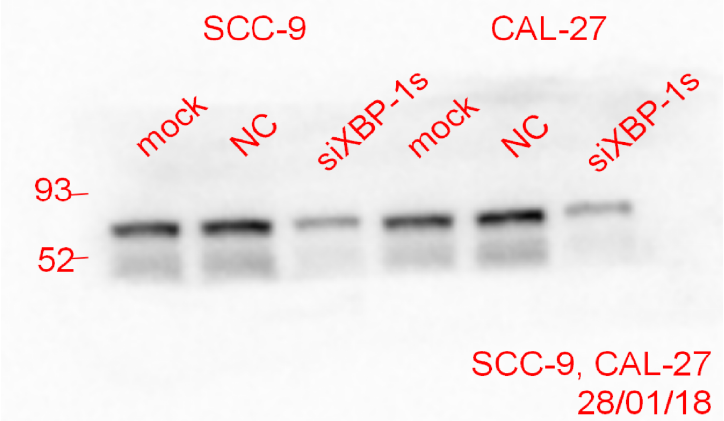

anti-MHC-I

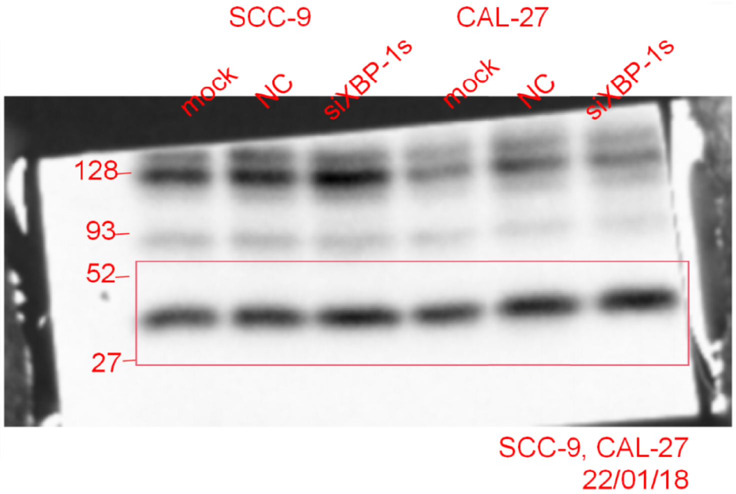

anti-XBP-1s

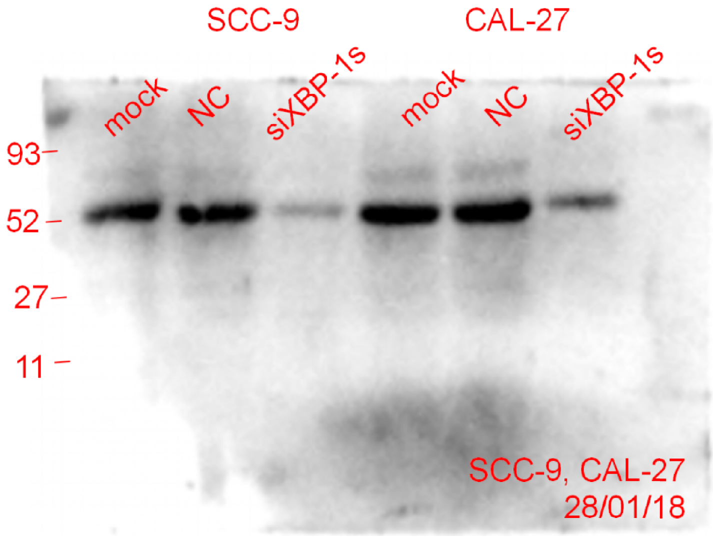

anti-GAPDH

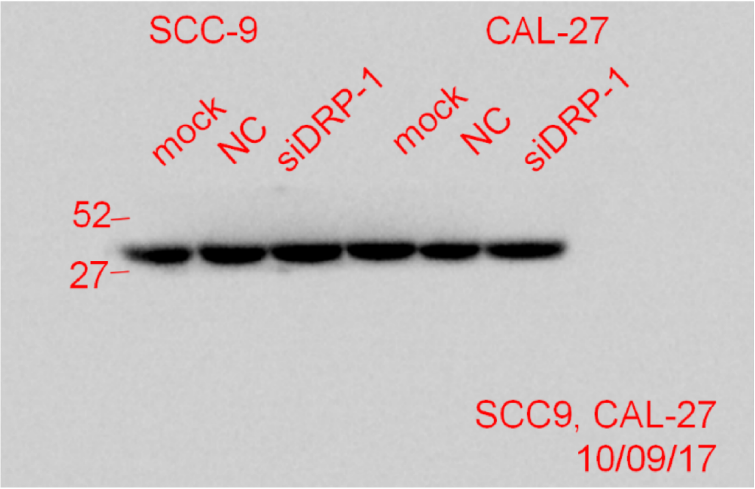

Raw Data Related to Supplementary Figure 6H

Same membranes are indicated by a black dashed box, and the cropped area is indicated by a red box. Membrane was cut.

anti-GRP78

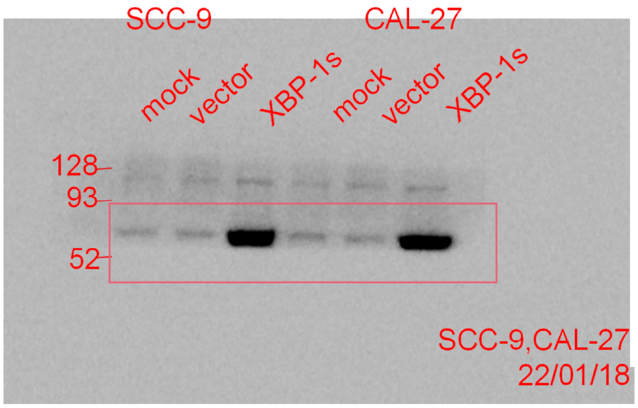

anti-MHC-I

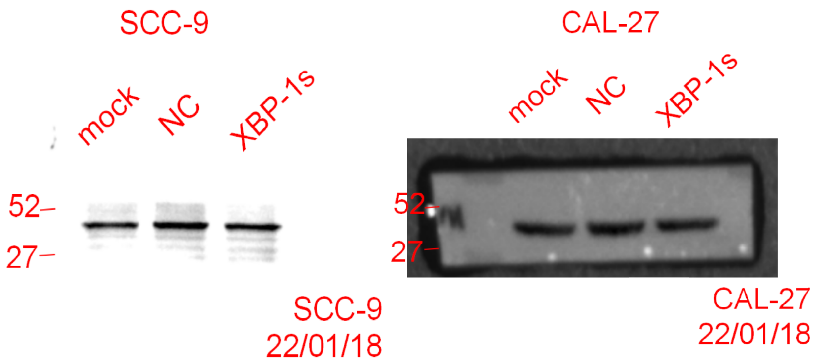

anti-XBP-1s

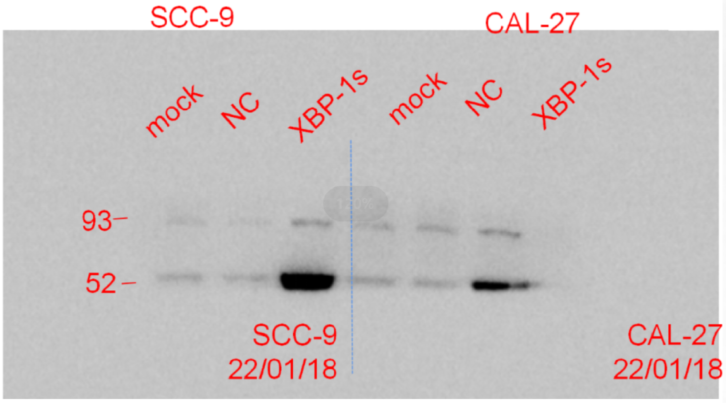

anti-CHOP

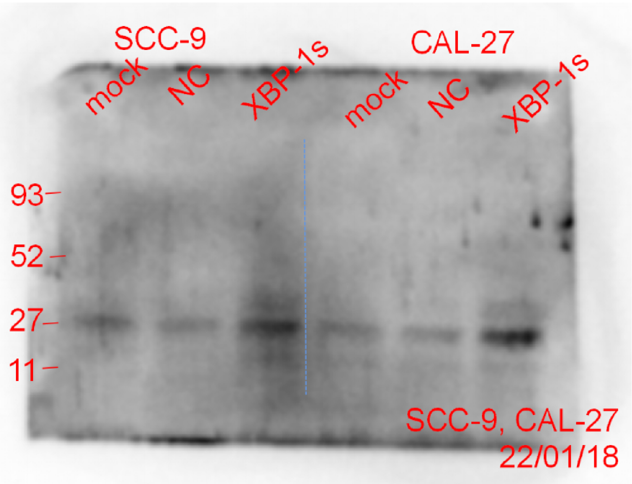

anti-GAPDH

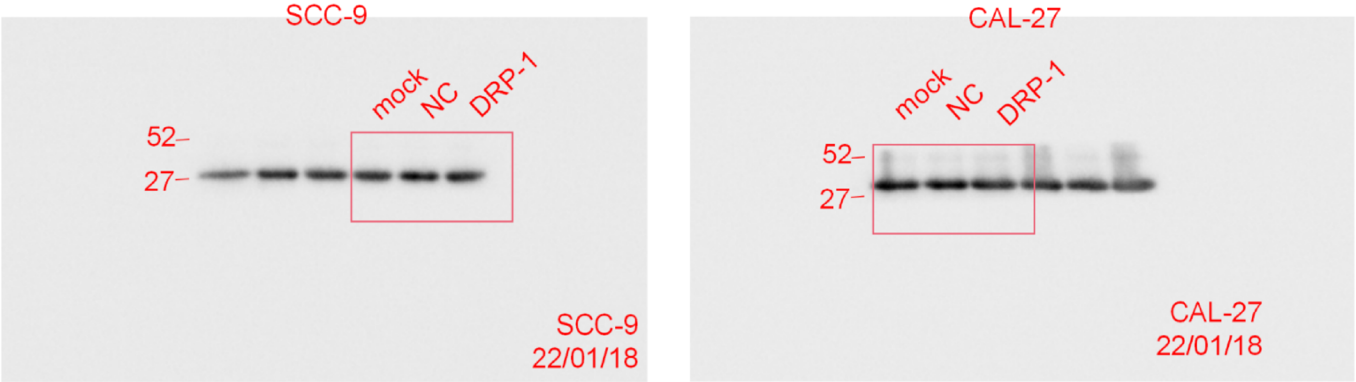

Gating strategy used for flow cytometry analysis

Gating strategies for flow cytometry analysis of tumours used in this study. Related to Figure 4H, 5C, 5F, 5H and supplementary Figure 2D.

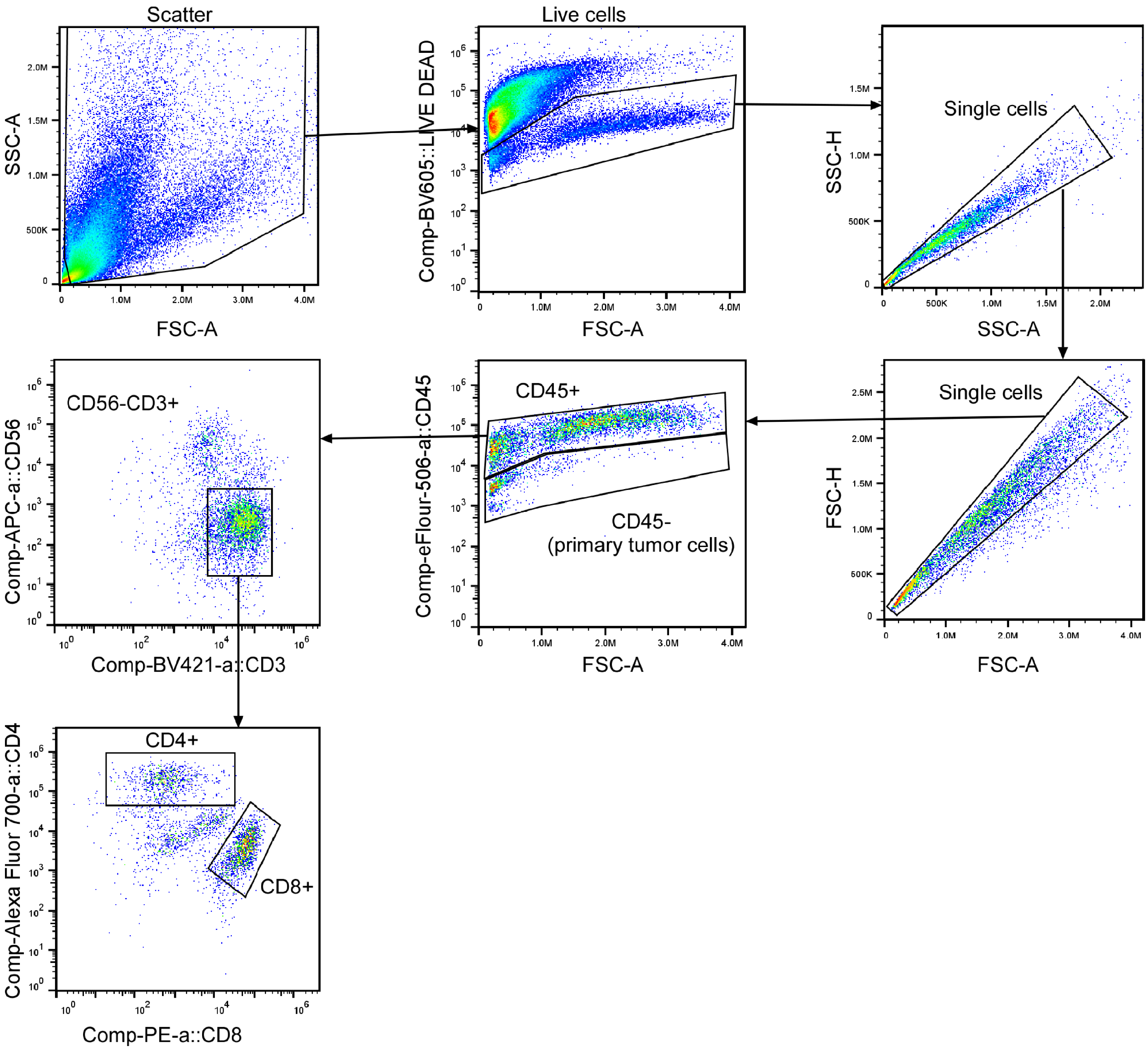

Supplement: Supplementary file 1 — Supplementary Information [file 41467_2022_31417_MOESM1_ESM.pdf]
