# Supplementary material for: Mitochondrial fission induces immunoescape in solid tumors through decreasing MHC-I surface expression
Source: Nat Commun. 2022 Jul 6;13:3882. doi: 10.1038/s41467-022-31417-x (PMC9259736; doi:10.1038/s41467-022-31417-x)
Supplement: Supplementary file 2 — Description of Additional Supplementary Files [file 41467_2022_31417_MOESM2_ESM.pdf]

Description of Additional Supplementary Files

File Name: Supplementary Data 1
Description: Information for the patients from whom the PDXs used in this study were generated

File Name: Supplementary Data 2
Description: Information for the patients from whom the Co-culture used in this study were generated
