# Supplementary material for: Mitochondrial fission induces immunoescape in solid tumors through decreasing MHC-I surface expression
Source: Nat Commun. 2022 Jul 6;13:3882. doi: 10.1038/s41467-022-31417-x (PMC9259736; doi:10.1038/s41467-022-31417-x)
Supplement: Supplementary file 5 — Reporting Summary [file 41467_2022_31417_MOESM5_ESM.pdf]

nature portfolio

Corresponding author(s): Song Fan,Soldano Ferrone,Jinsong Li

Last updated by author(s): Apr 15, 2022

Reporting Summary

Nature Portfolio wishes to improve the reproducibility of the work that we publish. This form provides structure for consistency and transparency in reporting. For further information on Nature Portfolio policies, see our Editorial Policies and the Editorial Policy Checklist.

Statistics

For all statistical analyses, confirm that the following items are present in the figure legend, table legend, main text, or Methods section.

n/a	Confirmed	
☐	☒	The exact sample size (n) for each experimental group/condition, given as a discrete number and unit of measurement
☐	☒	A statement on whether measurements were taken from distinct samples or whether the same sample was measured repeatedly
☐	☒	The statistical test(s) used AND whether they are one- or two-sided<br>*Only common tests should be described solely by name; describe more complex techniques in the Methods section.*
☐	☒	A description of all covariates tested
☐	☒	A description of any assumptions or corrections, such as tests of normality and adjustment for multiple comparisons
☐	☒	A full description of the statistical parameters including central tendency (e.g. means) or other basic estimates (e.g. regression coefficient) AND variation (e.g. standard deviation) or associated estimates of uncertainty (e.g. confidence intervals)
☐	☒	For null hypothesis testing, the test statistic (e.g. F, t, r) with confidence intervals, effect sizes, degrees of freedom and P value noted<br>*Give P values as exact values whenever suitable.*
☒	☐	For Bayesian analysis, information on the choice of priors and Markov chain Monte Carlo settings
☒	☐	For hierarchical and complex designs, identification of the appropriate level for tests and full reporting of outcomes
☐	☒	Estimates of effect sizes (e.g. Cohen's d, Pearson's r), indicating how they were calculated

Our web collection on statistics for biologists contains articles on many of the points above.

Software and code

Policy information about availability of computer code

Data collection	For most experiments, the person who performed the experiments did not know the identity of the specific samples until after data were analyzed. For patients' information, the data were downloaded from the hospital database and analyzed by the person who were blinded to the study design .
Data analysis	All data are expressed as the mean ± standard error of mean (s.e.m.). All statistical analyses were performed in SPSS Windows version 13.0. Spearman correlation analysis were used to assess the relationship between HLA-I and pSer616 DRP-1 expression. Kaplan–Meier survival curves were plotted and log-rank tests were performed. All experiments were performed at least in triplicates and the exact numbers of independent experiments with similar results are indicated in the figure legends. All statistical analyses of experiments were performed with two-tailed Student's T tests unless otherwise stated. p < 0.05 was considered statistically significant. Flow cytometry data were analyzed using FlowJo v10.4.0 software.

For manuscripts utilizing custom algorithms or software that are central to the research but not yet described in published literature, software must be made available to editors and reviewers. We strongly encourage code deposition in a community repository (e.g. GitHub). See the Nature Portfolio guidelines for submitting code & software for further information.

Data

Policy information about availability of data

All manuscripts must include a data availability statement. This statement should provide the following information, where applicable:

- Accession codes, unique identifiers, or web links for publicly available datasets
- A description of any restrictions on data availability
- For clinical datasets or third party data, please ensure that the statement adheres to our policy

Data have been deposited in the Gene Expression Omnibus (GEO) DataSets (https://www.ncbi.nlm.nih.gov/gds) under the following accession numbers: GSE135380.To predict the potential XBP-1 binding sites at TPP2 promoter region, we used motif-counter (https://bio.tools/motifcounter) to scan TPP2 promoter region from both strands with XBP-1 motif obtained from JASPAR database.

Field-specific reporting

Please select the one below that is the best fit for your research. If you are not sure, read the appropriate sections before making your selection.

☒ Life sciences ☐ Behavioural & social sciences ☐ Ecological, evolutionary & environmental sciences

For a reference copy of the document with all sections, see nature.com/documents/nr-reporting-summary-flat.pdf

Life sciences study design

All studies must disclose on these points even when the disclosure is negative.

Sample size	No statistical methods were used to predetermine sample size.
Data exclusions	No samples were excluded from analysis.
Replication	Experiments were repeated and our data are based on at least two to three independent experiments with similar results. The precise number of repeats are given in the figure legend.
Randomization	Mice of similar ages were randomly allocated into different groups using a computer-generated random numbers table.
Blinding	The investigator doing the injection was blinded to treatment arm. In addition, the person doing the daily drug treatment were blinded to the experiment design including groups. Tumor staining from ISH and IHC was performed by two blinded person. Data collection and analysis was blinded.

Reporting for specific materials, systems and methods

We require information from authors about some types of materials, experimental systems and methods used in many studies. Here, indicate whether each material, system or method listed is relevant to your study. If you are not sure if a list item applies to your research, read the appropriate section before selecting a response.

Materials & experimental systems

n/a	Involved in the study	
☐	☒	Antibodies
☐	☒	Eukaryotic cell lines
☒	☐	Palaeontology and archaeology
☐	☒	Animals and other organisms
☐	☒	Human research participants
☒	☐	Clinical data
☒	☐	Dual use research of concern

Methods

n/a	Involved in the study	
☒	☐	ChIP-seq
☐	☒	Flow cytometry
☒	☐	MRI-based neuroimaging

Antibodies

Antibodies used

For ChIP assays, we used anti-XBP-1 antibody (Santa cruz, sc-8015, 200 μg/0.1 ml) and anti-RNA polymerase II antibody (Abcam, ab5131, 2 μg for 25 μg of chromatin) .

For Flow cytometry, we used H-2Kb-FITC (Cat. No. MHC2163, JPT), SIINFEKL-H-2Kb-FITC (Cat. No. MA5-17999, Invitrogen),anti-Mouse CD8a-APC (Cat. No. 553035, BD Pharmingen,), anti-OVA-H-2Kb tetramer-BV421 (Cat. No. TB-5001-4, MBL), HLA-ABC-PE (Cat. No. 560168, BD Pharmingen), Perforin-BV421 (Cat. No. 563393, BD Pharmingen), Granzyme B-FITC (Cat. No. 560211, BD Pharmingen), CD8-PE-CF594 (Cat. No. 562282, BD Pharmingen), EpCAM-BV510 (Cat. No. 563181, BD Pharmingen), ITGB1-PE (Cat. No. 555443, BD Pharmingen), TfR1-APC (Cat. No. 561940, BD Pharmingen), E-Cadherin-BV421 (Cat. No. 743712, BD Pharmingen), CD80-PE (Cat. No.

560925, BD Pharmingen), CD83-BV421 (Cat. No. 562630, BD Pharmingen) and CD86-FITC (Cat. No. 560958, BD Pharmingen). For detection of apoptosis, cells were collected and fixed in 500 μL of Annexin V binding buffer containing 5 μL Annexin V-APC and 5 μL 7-AAD (Cat. No.640930, Biolegend).

For Immunoblotting assays, we used MHC-I (Cat. No. sc-32235, Santa Cruz, 1:200, Reacts with Human, Suitable for: WB, IP, IF,FCM), IRE1α (Cat. No. 3294, Cell Signaling Technology, 1:1000 Reacts with:Mouse, Rat, Human, Suitable for: WB, IP), pIRE1α (Cat. No. NB100-2323, Novus Biologicals, 1:1000, Reacts with: Mouse, Rat, Human,Suitable for: WB, ChIP, ELISA, IB, ICC/IF, IHC, IHC-Fr, IHC-P), XBP-1 (Cat. No. ab37151, Abcam, 1:200, Reacts with: Mouse, Rat, Human, Suitable for: ICC/IF, WB), XBP-1s (Cat. No. 619502, Biolegend, 1:500, Reacts with Human, Suitable for: WB, ChIP, ICC/IF), GRP78 (Cat. No. 3177, Cell Signaling Technology, 1:1000, Reacts with: Human, Mouse, Suitable for: WB,IHC(P), FCM), CHOP (Cat. No. 2895, Cell Signaling Technology, 1:1000, Reacts with: Mouse, Rat, Human,Suitable for: WB, IP, IF, F, ChIP), DRP-1 (Cat. No. 14647, Cell Signaling Technology, 1:1000, Reacts with: Mouse, Rat, Human, Monkey, Suitable for: WB), TPP2 (Cat. No. 66017-1-Ig, Proteintech, 1:1000, Reacts with Human, Suitable for:FCM, IF, IHC, WB, ELISA), GAPDH (Cat. No. 60004-1-Ig, Proteintech, 1:20000, Reacts with: Human, Mouse, Rat, Yeast, Plant, Zebrafish, Suitable for:FC, IF, IP, WB, ELISA), β-actin (Cat. No.20536-1-AP, Proteintech, 1:2000,Reacts with: Human, Mouse, Rat, Monkey, Canine, Suitable for:FCM, IF, IHC, WB, ELISA) , then with a peroxidase-conjugated secondary antibody (Cat. No. SA00001-1, SA00001-2,Proteintech,1:1000).

For Immunofluorescence and immunohistochemistry staining, we used HC-10+HC-A2 (targeting HLA-I, a generous gift from Dr. Koichi Sakakura, Massachusetts General Hospital, Harvard Medical School), pSer616 DRP-1 (Cat. No. bs-12702R-HRP, Bioss, 1:300, Reacts with Mouse, Suitable for:IHC-P, IHC-F), EpCAM (Cat. No. ab213500, Abcam, 1:16000, Reacts with: Mouse, Rat, Human, Suitable for: IHC-P, WB, IP, ICC/IF) and CD4 (Cat. No. 25229s, Cell Signaling Technology, 1:400,Reacts with Mouse Rat,Suitable for: WB,IHC), NK1.1 (Cat. No. WLH4257, Wanleibio, 1:200, Reacts with Mouse, Suitable for: IHC), CD25 (Cat. No. AF7675, Affinity, 1:200, Reacts with: Human, Mouse, Suitable for: WB, IHC, ELISA(peptide)), CD206 (Cat. No. 60143-1-Ig , Proteintech, 1:400, Reacts with: Human, Mouse, Suitable for: FC, IHC, IP, WB,ELISA), CD20 (Cat. No.bs-0080R, BIOSS, 1:100, Reacts with: Mouse, Rat, Human, Suitable for: WB, ELISA, FCM, IHC, IF). For immunofluorescence, specimens were incubated with MHC-I (Cat. No. sc-32235, Santa Cruz, 1:200,Reacts with Human, Suitable for: WB, IP, IF,FCM), pSer616 DRP-1 (Cat. No. 4494, Cell Signaling Technology, 1:3200, Reacts with Mouse, Suitable for:IHC-P, IHC-F), EpCAM (Cat. No. ab213500, Abcam, 1:100, Reacts with: Mouse, Rat, Human, Suitable for: IHC-P, WB, IP, ICC/IF)

Validation

The commercial antibodies have been used in the study and the validation reported on supplier's website.

Eukaryotic cell lines

Policy information about cell lines

Cell line source(s)

Human cancer cell lines SCC-9 (TSCC), CAL-27 (TSCC), A549 (NSCLC), Saos-2 (osteosarcoma) and mouse cancer cell line B16F10 (melanoma) were purchased from American Type Culture Collection (ATCC). Human melanoma cell lines Colo38, FO-1 and M21 were provided by Dr. Soldano Ferrone (Massachusetts General Hospital, Harvard Medical School).

Authentication

No cell lines were authenticated in these studies, but low passage number cell lines were utilized.

Mycoplasma contamination

All eukaryotic cell lines used in this study tested negative by PCR for mycoplasma contamination.

Commonly misidentified lines
(See ICLAC register)

No commonly misidentified cells lines were used in this study.

Animals and other organisms

Policy information about studies involving animals; ARRIVE guidelines recommended for reporting animal research

Laboratory animals

Female C57BL/6 mice of 5-6 weeks old that purchased from Laboratory Animal Center, Sun Yat-sen University were used as syngeneic models, with 6 mice per experimental group. Male Rag1-/- C57BL/6 mice (Cat. No.T004753) of 5-6 weeks were obtained from GemPharmatech, Jiangsu, China. Four-week-old female NOD/SCID mice purchased from Beijing Vital River Laboratory Animal Technology Co., Ltd. (Beijing, China).

Wild animals

This study did not involve the wild animals.

Field-collected samples

This study did not involve the field-collected samples.

Ethics oversight

All mouse experiments were reviewed and approved by the ethics boards and the Clinical Research Committee of Sun Yat-sen Memorial Hospital.

Note that full information on the approval of the study protocol must also be provided in the manuscript.

Human research participants

Policy information about studies involving human research participants

Population characteristics

Each patient has been followed up for at least 60 months. Additionally, tumor samples and peripheral blood samples obtained from 109 patients with HNSCC and 60 patients with NSCLC at Sun Yat-sen Memorial Hospital, Sun Yat-sen University between April 2015 and October 2018 were used for primary cancer cell and T cell isolation and analysis. Peripheral blood (15-20 mL) was obtained from each patient. Primary specimens were collected from patients with HNSCC and NSCLC who underwent tumor resection at Sun Yat-sen Memorial Hospital, Sun Yat-Sen University.

Recruitment

Patients diagnosed with HNSCC and NSCLC at at Sun Yat-sen Memorial Hospital, Sun Yat-sen University and signed the

	consent form.
Ethics oversight	All related procedures were performed with the approval of the Institutional Review Board (IRB) of SYSMH (approved protocol:2016158)

Note that full information on the approval of the study protocol must also be provided in the manuscript.

Flow Cytometry

Plots

Confirm that:

- [x] The axis labels state the marker and fluorochrome used (e.g. CD4-FITC).
- [x] The axis scales are clearly visible. Include numbers along axes only for bottom left plot of group (a 'group' is an analysis of identical markers).
- [x] All plots are contour plots with outliers or pseudocolor plots.
- [x] A numerical value for number of cells or percentage (with statistics) is provided.

Methodology

Sample preparation	Cells were stained with H-2Kb-FITC (Cat. No. MHC2163, JPT), SIINFEKL-H-2Kb-FITC (Cat. No. MA5-17999, Invitrogen), anti-Mouse CD8a-APC (Cat. No. 553035, BD Pharmingen,), anti-OVA-H-2Kb tetramer-BV421 (Cat. No. TB-5001-4, MBL), HLA-ABC-PE (Cat. No. 560168, BD Pharmingen), Perforin-BV421 (Cat. No. 563393, BD Pharmingen), Granzyme B-FITC (Cat. No. 560211, BD Pharmingen), CD8-PE-CF594 (Cat. No. 562282, BD Pharmingen), EpCAM-BV510 (Cat. No. 563181, BD Pharmingen), ITGB1-PE (Cat. No. 555443, BD Pharmingen), TfR1-APC (Cat. No. 561940, BD Pharmingen), E-Cadherin-BV421 (Cat. No. 743712, BD Pharmingen), CD80-PE (Cat. No. 560925, BD Pharmingen), CD83-BV421 (Cat. No. 562630, BD Pharmingen) and CD86-FITC (Cat. No. 560958, BD Pharmingen). For detection of apoptosis, cells were collected and fixed in 500 µL of Annexin V binding buffer containing 5 µL Annexin V-APC and 5 µL 7-AAD (Cat. No.640930, Biolegend). . For intracellular staining, cells were pretreated with the Intracellular Fixation and Permeabilization Kit (Cat. No. 88-8824, eBioscience) according to the manufacturer’s instructions.
Instrument	Cells were subsequently analyzed with multicolor flow cytometry (BD, FACSVerse).
Software	Data was collected using the ulticolor flow cytometry (BD, FACSVerse). Analysis of flow cytometry data was done using FlowJo10.4.0 (Treestar) software.
Cell population abundance	the cell population abundance was analysed within FSC-A and SSC-A plots.
Gating strategy	FACS gating strategy, first plot gating for live cells, then second plot for single and then according to different marker to set the gate.

- [x] Tick this box to confirm that a figure exemplifying the gating strategy is provided in the Supplementary Information.
